# A domain-level DNA strand displacement reaction enumerator allowing arbitrary non-pseudoknotted secondary structures

**DOI:** 10.1098/rsif.2019.0866

**Published:** 2020-06-03

**Authors:** Stefan Badelt, Casey Grun, Karthik V. Sarma, Brian Wolfe, Seung Woo Shin, Erik Winfree

**Affiliations:** 1 California Institute of Technology, Pasadena, CA, USA; 2 Wyss Institute, Harvard University, Boston, MA, USA; 3 David Geffen School of Medicine at UCLA, Los Angeles, CA, USA

**Keywords:** chemical reaction networks, dynamic DNA nanotechnology, molecular programming

## Abstract

Information technologies enable programmers and engineers to design and synthesize systems of startling complexity that nonetheless behave as intended. This mastery of complexity is made possible by a hierarchy of formal abstractions that span from high-level programming languages down to low-level implementation specifications, with rigorous connections between the levels. DNA nanotechnology presents us with a new molecular information technology whose potential has not yet been fully unlocked in this way. Developing an effective hierarchy of abstractions may be critical for increasing the complexity of programmable DNA systems. Here, we build on prior practice to provide a new formalization of ‘domain-level’ representations of DNA strand displacement systems that has a natural connection to nucleic acid biophysics while still being suitable for formal analysis. Enumeration of unimolecular and bimolecular reactions provides a semantics for programmable molecular interactions, with kinetics given by an approximate biophysical model. Reaction condensation provides a tractable simplification of the detailed reactions that respects overall kinetic properties. The applicability and accuracy of the model is evaluated across a wide range of engineered DNA strand displacement systems. Thus, our work can serve as an interface between lower-level DNA models that operate at the nucleotide sequence level, and high-level chemical reaction network models that operate at the level of interactions between abstract species.

## 1. Introduction

**Figure 1.**
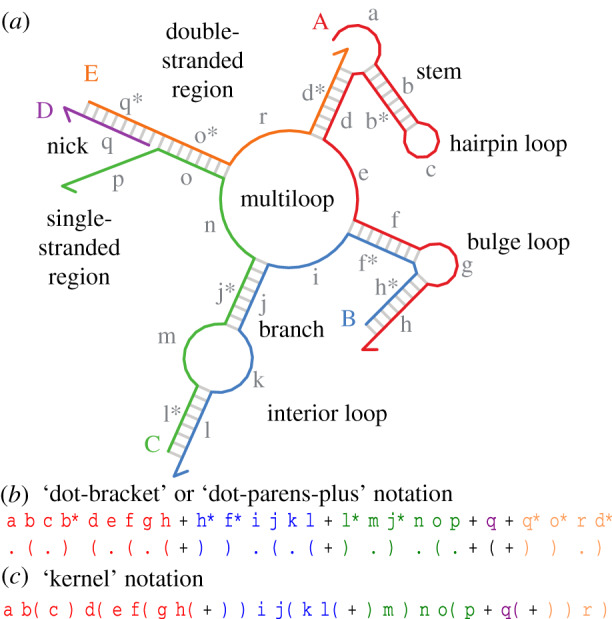
Nucleic acid secondary structure and common terminology (for formal definitions, see §2.1). (*a*) An example secondary structure with five strands (A, B, C, D and E) and lowercase-named domains (a–r), illustrating a variety of structural motifs supported by the enumerator. Arrowheads (⇀) indicate the 3’ end of each strand. (*b*) Multistranded complexes with their domain-level secondary structures are represented as string. The first line is a sequence of domains, the second line the corresponding structure (referred to as either ‘dot-bracket’ or ‘dot-parens-plus’ notation). Matching parentheses denote hybridized domains, ‘dots’ denote unpaired domains and ‘plus’ denotes the concatenation of two strands. (*c*) In this paper, we introduce an equivalent shorthand notation that interleaves domain-level sequence and domain-level structure, called **kernel notation**.

The evolution of DNA nanotechnology during the last few decades has shown DNA to be a robust and versatile substrate for nanoscale construction and computation [1]. It is a common abstraction to describe these DNA systems in terms of **domains**: contiguous sequences of nucleotides that are intended to participate in hybridization as one entity. Complementary domains are able to hybridize, and all other pairs of domains are not (figure 1). Once a system has been described in terms of domains, nucleotide sequences can be designed to optimize for the imposed domain-level complementarity rules [2,3]. However, prior to sequence design, domain-level systems can *and should* be analysed at the domain level. This is particularly relevant for so-called ‘DNA strand displacement’ systems, which have been used to implement digital and analogue computation in a well-mixed solution [4–8], and can be programmed using the formal language of chemical reaction networks (CRNs) [5,7,9]. Here, we call them domain-level strand displacement (DSD) systems, because we treat the domain level as an explicit formal abstraction layer with well defined semantics, which can be rigorously analysed without knowing the specific type of nucleic acid or polymer.

The term **enumeration** refers to the process of generating a CRN, given a finite set of initial complexes and a set of rules for their interactions. The enumerated CRN can then be (i) visually inspected to identify spurious and unintended reaction pathways, (ii) rigorously analysed to verify its correctness with respect to a formal CRN [10,11], or (iii) simulated to track expected species concentrations over time. In this contribution, we introduce the domain-level reaction enumeration software **Peppercorn**, which also provides an approximate rate model for domain-level reactions of DNA molecules.

Several previous efforts have been made to enumerate and simulate reaction networks for DNA nanotechnology at the domain level [12–20] and will be discussed further in §6.1. Among them, the most popular circuit analysis tool is **VisualDSD** [15–17], which supports a limited class of DNA structures (e.g. no hairpin-loops, no branched structures) and a built-in set of common intended reaction rules between those structures. More recently, VisualDSD can interpret a programming language called **LogicDSD** [18,20], which has been used to enumerate and simulate a much wider class of DNA related systems. For example, it supports DNA structures with arbitrary pairing between two complementary domains (including hairpins, branched structures and so-called pseudoknotted conformations; see definition 2.3), it supports enzymatic processes such as DNA degradation, etc. However, LogicDSD may require the user to have extensive prior knowledge about the system, both to formulate appropriate reaction rules for enumeration and to provide the reaction rates for simulation.

Peppercorn provides an out-of-the box domain-level reaction enumeration model that is more general than ‘classic’ VisualDSD, but less permissive than the LogicDSD language. Like other enumerators, Peppercorn provides a single type of bimolecular reaction: hybridization between two unpaired domains. However, in contrast to other enumerators, it provides an exhaustive set of intramolecular domain-level reactions within the space of pseudoknot-free nucleic acid secondary structures (opening and closing of helix domains, as well as three-way and four-way branch migration via proximal and remote toeholds; see §2.2). This class of secondary structures (see definition 2.3) is particularly important, as the vast majority of conformations will be sterically feasible and well modelled by a well-established DNA and RNA thermodynamic energy model [21], which is used by standard nucleic acid structure prediction software [22–24]. Furthermore, the biophysics of conformation changes for this class is well studied, e.g. [25–39], and Peppercorn provides an approximate kinetic model grounded in this understanding.

Thus, Peppercorn is an important step forward to bridge the gap between kinetic analysis of domain-level DNA nanotechnology and well-established nucleic acid sequence-level thermodynamic energy models and kinetic simulators. For example, the stochastic nucleic acid sequence-level reaction simulator **Multistrand** [40] is suitable for estimating the rate of individual strand displacement reactions, but it cannot cope with the massive state space of a complex multistranded DSD system. However, Peppercorn can be used as a preprocessing step to enumerate a domain-level reaction network, and then the individual reaction rates can be calculated using sequence-level simulators [41].

Peppercorn separates enumeration and simulation so that the exhaustive reaction network can be rigorously analysed. Combinatorial explosion due to implausible polymerization (figure 3) is controlled by enforcing a **separation of timescales**: assuming some reactions are much faster than others. This approximation is valid for low species concentrations, and can be performed either with or without reference to specific reaction rates, e.g. those that Peppercorn provides for domain-level DNA systems. Finally, Peppercorn uses this timescale separation to **condense** the detailed enumerated network with fast and slow reactions into a considerably smaller CRN with only overall slow reactions. We prove that those two CRNs are equivalent in terms of overall slow reaction pathways, and we provide a corresponding reaction rate condensation algorithm to simulate DSD systems on the more compact, condensed level.

We have implemented the Peppercorn enumerator in Python, available on GitHub [42], either as a standalone program for domain-level enumeration, or embeddable into other projects using the library interface. The **peppercornenumerator** library is already a central part of the **DyNAMiC Workbench** integrated development environment [43], the automated sequence-level verification software **KinDA** [41], and the ‘CRN-to-DSD’ compiler **Nuskell** [44]. Badelt *et al*. [44] use Nuskell (and thus Peppercorn) to enumerate and compare 13 different DSD systems implementing a DNA-only oscillator [7].

## 2. Reaction enumeration model

The following section introduces the different components of DSD systems, and the **kernel notation** for domain-level complexes and reactions. In §2.1, we introduce this notation as a compact representation for structures, and in §2.2 we use it to express reaction types as string modifications according to pattern-matching rules. §2.3 explains the assumptions that enable us to separate timescales for different reaction types with or without reference to specific reaction rates. In §2.4, we present a rule-based algorithm that supports all presented reaction types, and uses separation of timescales to enumerate the reaction network of a DSD system.

### 2.1. Primitives and definitions

DSD systems are abstract representations of reaction networks of interacting nucleic acid molecules. Intuitively, nucleic acids are represented as sequences of **domains**, as opposed to sequences of nucleotides. If domains are well designed, then each domain can hybridize as one entity only to its respective complementary domain, otherwise remaining unpaired. A **strand** is a sequence of domains that are connected with a covalent backbone, while the corresponding **structure** describes which domains are hybridized and which are unpaired. A **complex** is a structure that can be formed by one or more strands. Two complexes are different if they have either different strands or a different structure.

**Definition 2.1.**

A **domain** *d* = (*r*, *τ*) is a tuple where *r* is the name of the domain and *τ* is its length. A domain *d* = (*r*, *τ*) is *complementary* to domains of the form d* = (r*, *τ*) whose name is r* and type is the same. (We adopt the conventions that (d*)* = *d* ≠ d* and (r*)* = *r* ≠ r* and that there cannot be same-named domains with different lengths.) We distinguish two types of domains: **toehold domains** (or equivalently **short domains**) bind their complementary domain reversibly, i.e. both the hybridization rate and dissociation rate are relevant on the time scale of an experiment. **Branch-migration domains** (or equivalently **long domains**) bind their complementary domain irreversibly, i.e. the dissociation rate is treated as negligible.

Peppercorn considers domains with a length *τ* ≤ *L* nucleotides to be short domains, and all other domains are considered long. (By default *L* = 7 nucleotides.) While not done here, it would be natural to associate each domain with a specific nucleotide sequence so as to introduce a more refined, sequence-dependent kinetic model.

**Definition 2.2.**

A **strand** *s* = [*d*_1_, …, *d*_*m*_] is a sequence of *m* domains in specific order from 5′ to 3′ end.

**Definition 2.3.**

A **secondary structure** *π* for a set of strands *S* = {*s*_1_, …, *s*_*n*_} is a function π : (S×N)→(S×N)∪∅ that specifies, for each domain on each strand, the strand and domain (if any) to which it is bound. $\pi (s_{i},j)=\emptyset$ indicates that domain *d*_*j*_ on strand *s*_*i*_ is unbound. *π*(*s*_*i*_, *j*) = (*s*_*k*_, *l*) indicates that domain *d*_*j*_ on strand *s*_*i*_ is bound to domain *d*_*l*_ on strand *s*_*k*_. The domain and range of *π* must be restricted to only valid domains for each strand. Bindings must be consistent; that is, if *π*(*s*_*i*_, *j*) = (*s*_*k*_, *l*), then *π*(*s*_*k*_, *l*) = (*s*_*i*_, *j*). Furthermore, bindings must only occur between complementary domains; that is, if *π*(*s*_*i*_, *j*) = (*s*_*k*_, *l*), and *s*_*i*_[*j*] = *d*, then *s*_*k*_[*l*] = d*. Finally, a structure *π* is deemed **non-pseudoknotted** if there exists a specific order of strands in *S* (without loss of generality, let the ordering be *s*_1_, …, *s*_*n*_), such that all bindings in *π* are **nested** in the following sense. We say (strand index, domain index) pairs (*i*, *j*) > (*k*, *l*) if *i* > *k* or if *i* = *k* and *j* > *l* (i.e. pairs are compared lexicographically). A structure *π* is non-pseudoknotted if the following nesting condition applies for all pairs in *π*: if *π*(*s*_*i*_, *j*) = (*s*_*k*_, *l*) and *π*(*s*_*p*_, *q*) = (*s*_*u*_, *v*) and (*i*, *j*) < (*p*, *q*) < (*k*, *l*), then (*i*, *j*) < (*u*, *v*) < (*k*, *l*). If a secondary structure *π* is *not* non-pseudoknotted, then *π* is **pseudoknotted**.

**Definition 2.4.**

A **complex** is a specific secondary structure formed either by one strand, or by multiple strands that are **connected** by bound domains. Two strands *s*_*i*_ and *s*_*k*_ are directly bound if there is at least one domain in each strand that is bound to a domain in the other strand; that is, there exist *j* and *l* such that *π*(*s*_*i*_, *j*) = (*s*_*k*_, *l*). Two strands *s*_*i*_ and *s*_*k*_ are connected if either *s*_*i*_ is directly bound to *s*_*k*_ or *s*_*i*_ is directly bound to some other strand that is connected to *s*_*k*_.

This work only considers non-pseudoknotted secondary structures. The primary reason we restrict attention to non-pseudoknotted structures is that, after domains have been given specific nucleotide sequences, they naturally correspond (with a few exceptions^1^) to sterically unconstrained molecular geometries for which the thermodynamic free energy can be evaluated accurately using the standard nearest neighbour energy model [21,23]. In contrast, pseudoknotted secondary structures imply loop constraints and steric conflicts that necessitate incorporating additional (and possibly large) geometry-dependent energy terms [45–47]. Simplified approximations allow sometimes-accurate estimation of energies for certain sub-classes of single-stranded and multi-stranded pseudoknots [45,48–51], and incorporating such sub-classes would be an interesting extension of this work; however, doing so would also have many non-trivial implications for reaction enumeration as discussed below. The following definition 2.5 introduces a convenient string representation for non-pseudoknotted structures as introduced in definition 2.3 above.

**Definition 2.5.**

The pairings of a non-pseudoknotted secondary structure (as in definition 2.3) can always be written as a well-formed string where dots denote unpaired domains and matching parenthesis denote paired domains. When multiple strands are present, then the plus sign marks a strand break (i.e. the 3’ end of one strand and the 5’ end of the next strand; see figure 1*b*), and the strands must be listed in an order consistent with the nesting condition of definition 2.3. The **kernel notation** is a compact representation where domain-level sequence and structure are interleaved such that a domain written by itself is unpaired, while a domain followed by parenthesis is part of a duplex; the matching closing parenthesis indicates the bound complementary domain of the duplex. (The domain name is not written explicitly since it is implied.) A **well-formed substructure** is a subsequence of a kernel string that by itself is a well-formed kernel string, i.e. each opening parenthesis has a unique matching closing parenthesis.

Note that in a kernel string, anything between two matching parenthesis is a well-formed substructure. A kernel string (and thus a substructure) can represent multiple (disconnected) complexes. A well-formed non-pseudoknotted complex composed of *k* strands has exactly *k* equivalent representations where paired elements are properly nested, corresponding to the *k* circular permutations of the strands [23]. As an example, there are five circular permutations of the complex shown in figure 1, shown here with each strand in a different colour as per the figure:


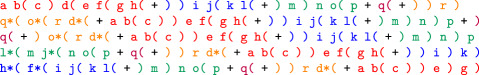


Importantly, since the same complex may be represented in several ways, all operations discussed in later sections are considered to act independent of representation, but may be defined with respect to a convenient representation.

**Definition 2.6.**

A **reaction** *r* = (*A*, *B*) is a tuple where *A* is the multiset of reactants and *B* is the multiset of products. The **arity** *α*(*r*) of a reaction *r* is a pair (|*A*|, |*B*|), where |*A*| denotes the number of molecules in *A*. Any reaction with arity (1, *n*) is **unimolecular**; reactions with arity (2, *n*) are **bimolecular**, and those with other arities are *higher order*. We say a reaction **conserves strands** (and thus conserves mass) if the multiset of strands that appear in reactants equals the multiset that appear in products. Each reaction may be classified as **fast** or **slow**; unimolecular reactions may be either fast or slow, while bimolecular and higher-order reactions must be slow. For a set *R* of reactions, we sometimes write *R*_*f*_ to represent the fast reactions and *R*_*s*_ to represent the slow reactions, such that $R=R_{f}\cup R_{s}$. Finally, it will sometimes be useful to partition *R*_*f*_ into (1, 1) and (1, *n* > 1) reactions, such that $R_{f}=R_{f}^{\left(1,1\right)}\cup R_{f}^{\left(1,n>1\right)}$, where by convention (1, *n* > 1) indicates reactions with any value of *n* greater than 1. A **detailed reaction** is a reaction where reactants and products are complexes. A **condensed reaction** is a reaction between *resting macrostates* that will be introduced in definition 2.7.

All reactions considered in this paper conserve strands; as a consequence, there will be no (0, *n*) or (*n*, 0) reactions, as those would birth new products from no reactants or cause all reactants to disappear. The distinction between fast and slow reactions is motivated by a separation of timescales that occurs in the limit of low concentrations, as will be discussed in §2.3.

**Definition 2.7.**

A **chemical reaction network (CRN)** is a pair *G* = (*C*, *R*) where *C* is a set of species (either complexes or macrostates) and *R* is a set of reactions between those species. For CRNs with reactions labelled as fast or slow, as per definition 2.6, we consider an associated directed graph $\Gamma =(C,R_{f}^{\left(1,1\right)})$ with nodes *C* corresponding to the set of species in the CRN *G* and edges $R_{f}^{\left(1,1\right)}$ being only the set of fast (1, 1) reactions. The strongly connected components (SCCs) of Γ define a set of macrostates: a SCC is called a **transient macrostate** if *G* contains a fast (1, 1) or (1, *n* > 1) reaction leaving the SCC, and is called a **resting macrostate** otherwise. When *C* is a set of complexes, we refer to *G* as a **detailed CRN**, while if *C* is a set of resting macrostates and R is a set of condensed reactions then we call it a **condensed CRN**.

The justification for using only (1, 1) reactions, and not other (1, *n* > 1) reactions, when calculating the SCCs is that reactants and products of fast (1, *n* > 1) reactions cannot both be in the same SCC due to strand conservation.

**Figure 2.**
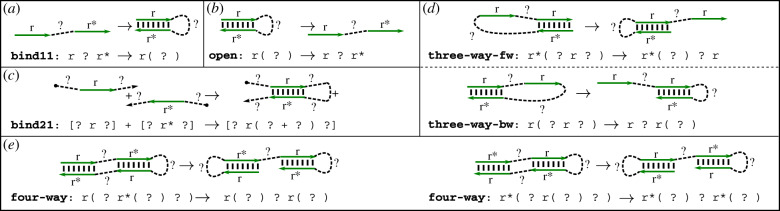
Available reaction types and their corresponding pattern matching rules. The wildcard character ‘?’ is always shown as a connected dotted line, although depending on context it may represent a non-connected substructure. Domains that change their configuration according to the corresponding pattern matching rule are shown as straight, green, directed arrows. (*a*) Unimolecular binding. (*b*) Unimolecular opening. The pattern matching rule is the exact reverse of unimolecular binding. (*c*) Bimolecular binding. The pattern requires a more explicit formulation than unimolecular binding to ensure a well-formed kernel representation of the product complex. (*d*) Three-way branch migration. Two distinct rules are necessary to describe the forward and reverse reaction. The patterns for the product of the forward reaction and for the reactant of the reverse reaction are circular permutations. (*e*) Four-way branch migration. One rule describes both forward and backward reactions, as the reactant pattern is a circular permutation of the product pattern.

*Kernel notation for reactions.* Reaction mechanisms can be specified with kernel notation; for example: a( b + b( c + )) -> b c + a( b( + )) is a unimolecular three-way branch migration reaction with two products. The next section (and figure 2) will explain all reaction types in detail, but it is worth drawing the corresponding complexes of this reaction to get familiar with kernel representations.

### 2.2. Reaction types

Imagine an experimental setting with a test tube of complexes as the initial state of a CRN. This section defines an exhaustive set of *intended* reaction types that can occur under the assumption that the nucleotide-level sequences are designed to implement the domain-level logic. Hence, a reaction type represents a distinct molecular mechanism for intramolecular or intermolecular configuration changes, also shown in figure 2. Using our previously introduced kernel notation, we can formulate this set of reactions as string modification rules.

*Pattern-matching rules.* A reaction matching rule is a pair of patterns separated by an irreversible reaction arrow, ‘->’. The rules describe permitted reactions, which transform one multiset of complex(es) (matched by the reactant pattern) into another (matching the product pattern). Patterns are written in a generalized version of the kernel notation (figure 2). In addition to named domains (e.g. ‘r’), their complements (e.g. ‘r*’), and hybridization markings (e.g. ‘r(’ and ‘)’), here we introduce the wildcard ‘?’ to match a well-formed substructure of a complex (which may or may not in and of itself be connected). Additionally, the brackets ‘[’ and ‘]’ denote a 5′ or 3′ end of a complex, respectively. As usual, ‘+’ is a strand break. We distinguish two types of pattern-matching rules: unimolecular, in which the brackets cannot be used, and bimolecular, in which each reacting complex must appear inside brackets. Pattern-matching rules must satisfy the following criteria

(i) Both patterns are linear sequences that start and end with a non-wildcard character.
(ii) Both patterns have the same total number of domains and wildcards.
(iii) The domains and wildcards in both patterns occur in the same order (including implicit domains at closing parentheses).
(iv) Wildcards cannot have associated parentheses; only named domains indicate structure.
(v) Both patterns (for unimolecular rules), and each pattern enclosed in brackets (for bimolecular rules), must represent well-formed structures when the wildcards are removed.

To apply a pattern-matching rule to a complex containing *k* strands, we must test to see if the pattern matches *any* of the *k* representations that are equivalent up to circular permutation. For unimolecular rules, the pattern may appear anywhere *within* the complex, while for bimolecular rules, each complex must fully match its respective pattern within brackets. To match, each named domain in the pattern must be assigned to a single domain (or domain complement) from the complex, consistently for each occurrence of the domain in the pattern, while each wildcard must be assigned to a well-formed substructure from the complex, independently for each occurrence of a wildcard in the pattern. Thus, for each representation, there may be zero, one, or more ways to match the left-hand side pattern to the complex(es). Each such match represents a possible reaction; to obtain the reaction, the enumerator can instantiate the right-hand side pattern by substituting corresponding wildcards, then separating the right-hand side into multiple connected complexes if that is possible. There may be multiple ways to get the same reaction via different representations; only one copy of each distinct reaction is kept.^2^

For example, applying the pattern matching rule r*( ? r ? ) -> r*( ? ) ? r to the complex a( b + b( c( + ) b*+ )) yields two reactions. Matching r*( ? r ? ) = b( c( + ) b* + ) with, respectively, r = b*, ? = c( + ), ? = + yields a( b + b( c( + ) ) + b* ), which separates into a( b + b* ) and b( c( + )). The second reaction derivation begins with the circularly permuted c*( b* + b*( a*( + ) b + ) ), matches r*( ? r ?) = b*( a*( + ) b +) with, respectively, r = b, ? = a*( + ), ? = +, and yields c*( b* + b*( a*( +) ) + b ), which separates into c*( b* + b ) and b*( a*( + ) ).

*Bind reactions.* Two complementary, unpaired domains hybridize to form a duplex. We distinguish two types of bind reactions: bind11 is a bind reaction between two domains on the same complex, i.e. a reaction with arity (1, 1), while bind21 is a bimolecular bind reaction with one product (figure 2*a*,*c*).

— bind11: r ? r* -> r( ? )
— bind21: [? r ?] + [? r* ?] -> [? r( ? + ? ) ?]

We employ explicit end-of-strand markers for bind21 reactions to enforce that the left-hand side must match two separate complexes; this ensures that the kernel representations of those complexes will be rotated individually to ensure that domains r and r* are not enclosed by paired domains, such that the representation of the product complex is well formed.

*Open reactions.* Two paired domains detach (figure 2*b*). Open reactions are the reverse of previously discussed bind reactions, which means there are situations where opening happens without changing the arity of a reaction and other situations where opening results in the dissociation of two complexes.

— open: r( ? ) -> r ? r*

The enumeration semantics (discussed in more detail in §2.4) determine when the open reaction rule applies. In the default rate-independent semantics, open applies only when r is a short domain and thus is a fast reaction; in §2.3, we introduce rate-dependent semantics, where open reactions are enumerated for domains of any length, but reactions with a too-slow rate constant are discarded.

*Branch-migration reactions.* We distinguish two branch migration reaction types [28]. In a three-way branch migration, an unpaired domain displaces another instance of the same domain that is bound in a duplex (figure 2*d*). A four-way branch migration is a rearrangement where two hybridized domains exchange their binding partners at the same time (figure 2*e*), i.e. a reaction that proceeds via a four-way junction [52]. Branch migration reactions can result in a complex that remains connected, with reaction arity (1, 1), or they can disconnect into two complexes, with arity (1, 2). The latter are inherently irreversible.

— three-way-fw: r ? r( ? ) -> r( ? r ? )
— three-way-bw: r( ? r ? ) -> r ? r( ? )
— four-way: r( ? ) ? r( ? ) -> r( ? r*( ? ) ? )

Note that both branch migration reactions are reversible for reaction arity (1, 1), but the four-way branch migration reaction is symmetric, i.e. a circular permutation can transform the reactant pattern into the product pattern. This transformation is not possible for three-way branch migration reactions, which is why we distinguish the three-way-fw and three-way-bw patterns (figure 2*d*,*e*).

### 2.3. Separation of timescales

**Figure 3.**
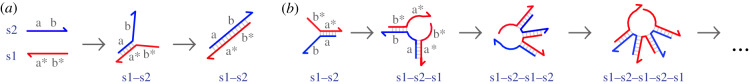
Enumeration with and without timescale separation. (*a*) Intended behaviour of two complementary strands s1 and s2. Either domains a and $a^{*}$ (as shown) or b and $b^{*}$ bind via a slow bimolecular reaction, followed by a fast unimolecular hybridization reaction of the respective other domain. (*b*) Pathological enumeration behaviour without a separation of timescales. Repeated bimolecular association reactions occur before the unimolecular bind reaction, generating implausibly long polymers. The kinetically and thermodynamically favoured simple duplex may never be found.

We use a timescale separation principle to avoid combinatorial explosion of the reaction network enumeration (figure 3) while maintaining the generality of reaction types and secondary structures considered. Intuitively, complexes have infinite time to engage in fast reactions, before they engage in a slow reaction. In order to increase the applicability of the model, we present reaction enumeration semantics that can be justified *with or without* reference to specific values for specific reaction rates.

*Rate-independent model.* Our default model declares unimolecular reactions to be fast, while bimolecular reactions are slow (see definition 2.6). Note that unimolecular open reactions for long domains (as defined by the threshold parameter *L*) are excluded from enumeration. This simple model avoids consideration of a large number of unlikely reaction pathways that involve biomolecular reactions between transient intermediate complexes. For instance, it significantly reduces the problem of potentially infinite polymerization. Also, since the enumeration of unimolecular reactions is linear in the number of species, while enumeration of bimolecular reactions is quadratic, eliminating the consideration of bimolecular reactions between selected complexes effectively reduces the complexity of the enumeration problem.

We can justify this classification of unimolecular reactions as fast compared to bimolecular reactions in the limit of low concentrations. Consider a standard mass action model of chemical kinetics with fixed rate constant $k_{\alpha }$ for each reaction *α*, wherein bulk concentrations are continuous variables whose evolution proceeds according to ordinary differential equations (ODEs). Then the rate of a unimolecular reaction *α* with reactant *X* will be $k_{\alpha }[X]$, while the rate of a bimolecular reaction *β* with reactants *X* and *Y* will be $k_{\beta }[X][Y]$, where [*X*] and [*Y*] are the respective concentrations of *X* and *Y*. Consequently, as the concentration of all species decreases, the rates of bimolecular reactions decreases more quickly than the rates of unimolecular reactions, and eventually

$$k_{\beta }[X][Y]\ll k_{\alpha }[X].$$

Thus, in the limit of low concentrations, the bimolecular reaction will be much slower than the unimolecular reaction. A similar argument can be made for stochastic dynamics in the discrete regime [53]. As an example, consider typical rate constants for binding and dissociation of short domains at 25°C as estimated using the approximate formulae *k*_*β*_ = 3ℓ × 10^5^ M^−1^ s^−1^, and $k_{\alpha }=k_{\beta }\times e^{\frac{\Delta G}{RT}}\text{M}\approx 7.41\ell \times 10^{6}\times {\text{e}}^{-2.86\ell }\;{\text{s}}^{-1}$, where ℓ is the length of the domain (see appendix §C.1). For this rate model, the bimolecular reactions are more than an order of magnitude slower than the unimolecular reactions when all concentrations are less than 10^−1.24ℓ^ M. Consequently, for typical toehold lengths ℓ ≤ 7, our assumptions are valid in the low nanomolar concentration regime, e.g. with [*X*] = [*Y*] = 10^−8^ M, we have *k*_*β*_ (10^−8^ M)^2^ ≪ *k*_*α*_ (10^−8^ M).

*Rate-dependent model.* In this alternative model, the user provides two threshold values *k*_slow_ and *k*_fast_ to separate timescales based on unimolecular rate constants *k*_uni_ (as estimated e.g. by the model in appendix §C.1). As before, all bimolecular reactions are slow, but now unimolecular reactions fall into three categories: negligible (*k*_uni_ < *k*_slow_), slow (*k*_slow_ ≤ *k*_uni_ < *k*_fast_) and fast (*k*_fast_ ≤ *k*_uni_). In particular, *k*_slow_ is a threshold to exclude unimolecular reactions based on their rate constants (as opposed to the threshold parameter *L* in the rate-independent model), while *k*_fast_ separates slow unimolecular reactions from fast unimolecular reactions. That is, *k*_slow_ and *k*_fast_ give the lowest acceptable rate constant for unimolecular reactions in their respective category. Importantly, threshold *L* in the rate-independent model only applies to the open reaction, while *k*_slow_ and *k*_fast_ apply to all unimolecular reactions in the rate-dependent model.

In effect, by categorizing some unimolecular reactions as slow, the rate-dependent model enables us to model systems that would not work in a low concentration regime. For example, programmable hairpin systems such as the hybridization chain reaction [54] and catalysed three-arm junction formation [55] (see §5.3), as well as cooperative hybridization and strand displacement [56,57] involve two independent bimolecular interactions that are fully reversible by one or more unimolecular steps. But if all unimolecular reactions are fast, and if all fast reactions occur before any slow reactions, then such two-step reactions will always revert before the second step can take place. Thus, only if at least one of the unimolecular reverse reactions is considered slow, with *k*_slow_ ≤ *k*_uni_ < *k*_fast_, then both bimolecular reactions can happen sequentially in the model.

### 2.4. Reaction enumeration algorithm

A reaction enumerator can be considered to be a function that maps a set of initial complexes *C*_0_ to a CRN *G* = (*C*, *R*), where *C* is the set of complexes that appear in the network and *R* is the set of reactions between those complexes. Recall definitions 2.6 and 2.7 from §2.1, which state how to coarse-grain a CRN *G* = (*C*, *R*) with species (microstates) *C* and reactions *R* into transient macrostates and resting macrostates. A complex is a **resting complex** if it is in a resting macrostate, or a **transient complex** otherwise. Our reaction enumeration algorithm returns a unique CRN with the following properties:

(i) every complex has all valid fast reactions enumerated,
(ii) transient complexes have no slow reactions enumerated,
(iii) resting complexes have all valid slow reactions enumerated, and
(iv) all initial complexes are included,

where the notion of valid, fast, and slow reactions is referred to as **enumeration semantics**. The implementation is a rule-based approach, where fast reactions are always enumerated exhaustively from every species in the system, then SCCs are identified using Tarjan’s algorithm [58], resting complexes are identified, and all slow reactions between resting complexes are enumerated. New products have their fast neighbourhood enumerated first, and if new resting complexes have been found, new slow reactions are enumerated.

*Limitations.* Unfortunately, in this most general enumeration model, enumeration is critically dependent on the domain-level representation of the real system. For example, a long domain cannot engage in an open reaction, but if it is represented as a sequence of consecutive short domains then they can all open via a sequence of fast reactions. This is problematic, because now an actually stable complex will be classified as transient and cannot engage in slow reactions. We provide a solution called max-helix semantics for this example, but not for other more ambiguous cases where related problems still exist (see below).

#### 2.4.1. Enumeration semantics

Different reaction enumeration semantics enable us to vary the size of the state space in a controlled manner and are available as model parameters. For example, one can vary the release-cutoff, i.e. the threshold for open reactions *L*, or exclude a branch-migration reaction type, e.g. with ignore-branch-4way. Other systems might require the user to choose a max-complex-size, such that all reactions producing larger complexes are ignored. The reject-remote semantics excludes so-called remote-toehold branch migration [35], where the invading domain and its complement are not directly adjacent to already bound domains (figure 7). Thus with reject-remote semantics, only ‘proximal’ branch migration, i.e. adjacent to a bound domain, is permitted. This option may exclude states and reactions of biophysical importance, but can be especially useful for debugging unintended behaviour of a DSD system as many systems are designed without remote-toehold interactions in mind. See appendix §A on how reject-remote semantics affects enumeration. Using k-slow and k-fast switches to rate-dependent semantics, where unimolecular transition rates are classified into negligible, slow and fast reactions based on their estimated rate constants.

The **max-helix** notion extends every reaction of a single domain to propagate through all neighbouring domains compatible with that same reaction type. In other words, the pattern matching rules for reaction types remain the same, but the characters r, r*, r(,) do not match single domains, but instead match maximal sequences of consecutive domains. There are several advantages to using max-helix semantics: (i) enumeration is faster, as fewer states are explored and therefore fewer reactions are enumerated, (ii) any system enumerated using max-helix semantics can be modified by dividing its domains into subdomains (e.g. d → d_1_d_2_d_3_), while the state space and enumerated reactions remain the same, (iii) as a consequence of (ii), max-helix semantics yields a biophysically reasonable resting complex assignment whenever a sequence of fast reactions can be combined into one slow reaction.

However, max-helix does not solve the following ambiguous problems: (i) the same system can have an unexpectedly different state space when enumerated with or without max-helix semantics. (ii) Max-helix semantics is not a guarantee that toehold domains remain bound, if they are part of a long stem, as it only excludes the specific reaction opening that toehold. (iii) It is still possible to design complexes that in reality would be stable, but which would be considered transient in the max-helix model—for example, multiple short domains that are not adjacent can open as individual steps. Note, that cases (ii) and (iii) might actually cause troubles in a physical DSD system, so it is perhaps fortunate that our enumerator points out these issues. These and other details concerning max-helix semantics are discussed further in appendix §A and figure 12. The option no-max-helix turns max-helix semantics off, and can be especially useful to investigate partial unbinding of long domains.

#### 2.4.2. Premature termination

The enumerator provides a threshold to limit the maximum complex size of products, in order to (at least partially) enumerate systems which result in genuine polymerization, such as the hybridization chain reaction [54] and insertional polymerization [59]. However, some systems might simply get too big in the number of reactions and products. In order to detect and report such behaviour, the enumerator places a soft limit on the maximum number of complexes and the maximum number of reactions that can be enumerated before the enumerator will exit. These limits are checked before the neighbourhood of fast reactions is enumerated, which ensures that the CRN enumerated up to that point can still be investigated, for example by reaction condensation (as discussed in §3). However, when the maximum number of complexes or reactions is reached, there is no longer a reliable notion of *completeness* of the enumeration, which can be problematic.

## 3. CRN condensation

Consider a coarse-grained representation of a CRN, where we distinguish transient macrostates and resting macrostates. The condensed CRN is a projection of the original CRN that describes the overall reactions between resting macrostates. A condensed CRN is an intuitive way to formulate DSD systems, either for compact visualization or as a basis to prove/disprove the equivalence of CRN [10,11,44,60]. We present a rigorous self-contained theory that is independent of DSD enumeration, but requires certain properties of the original, detailed CRN to which the coarse-graining and condensation algorithm is applied:

(i) Reactions can have any arity (*n*, *m*), as long as 1 ≤ *n* ≤ 2 and *m* > 0.
(ii) All fast reactions are unimolecular.
(iii) Reactants of slow (unimolecular and bimolecular) reactions must be resting complexes.
(iv) For any sequence of unimolecular reactions, where each reaction consumes a product of the previous reaction and the last reaction produces the original species, the sequence must consist only of 1-1 reactions.^3^

**Figure 4.**
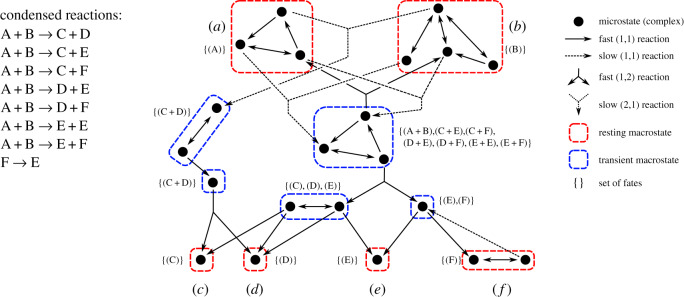
Trajectory-based CRN condensation. Fast reactions between microstates (nucleic acid complexes) determine the SCCs of a graph; terminal SCCs are resting macrostates. The result is a directed acyclic graph, where the set of fates for each complex can be calculated recursively for every macrostate. A condensed reaction exists for every slow reaction and every fate of the product of a slow reaction. Pathways of reactions that have the same reactants and products, such as {A + B → A + B, F → F}, are not included in the condensed CRN, but they are important for the calculation of condensed reaction rates.

**Figure 5.**
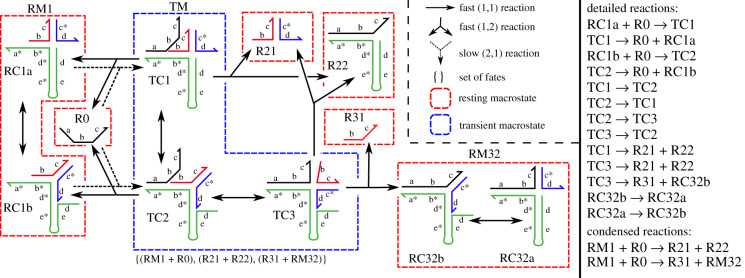
Condensation of a small DSD system with two alternative condensed reactions. Resting macrostates can contain more than one complex, e.g. RM1 contains two resting complexes: {RC1a, RC1b}. Whenever a resting macrostate contains only one complex, we use the same name for the macrostate and the complex. As discussed in the main text, the fate of a resting macrostate is the resting macrostate itself, while the fate of a transient macrostate (here: TM) is the set of resting macrostate combinations reachable via fast reactions. After RM1 and R0 react in the detailed network, the product of the condensed reaction is not yet determined, a phenomenon sometimes referred to as ‘delayed choice’.

The reaction enumeration algorithm presented in §2.4 yields a detailed CRN that satisfies these properties, even when the enumeration terminates prematurely. This section explains a rate-independent, trajectory-based projection of a detailed CRN into a condensed CRN, which is also illustrated in figures 4 and 5; the calculation of condensed reaction rates is discussed separately in appendix §C.2. For a formal correspondence between trajectories in the detailed CRN and its condensed representation see appendix §B.

*Coarse-graining of CRN.* The definitions 2.6 and 2.7 in §2.1 state how to coarse-grain a CRN *G* = (*C*, *R*) with species *C* and reactions *R* into transient macrostates and resting macrostates. We define the corresponding condensed CRN to be $\hat{G}=(\hat{C},\hat{R})$, where $\hat{C}$ is the set of resting macrostates and $\hat{R}$ is the set of condensed reactions. Recall that $R=R_{f}\cup R_{s}$, where *R*_*f*_ are fast reactions and *R*_*s*_ are slow reactions. We write fast reactions with arity (1,1) as $R_{f}^{\left(1,1\right)}$ and $\Gamma =(C,R_{f}^{\left(1,1\right)})$ for a directed graph that contains only the subset of $R_{f}^{\left(1,1\right)}$ reactions. Additionally, denote S(x) as the SCC of *Γ* containing some complex *x*. Hence, complex *x* is either a **resting complex** if S(x) is a resting macrostate, or a **transient complex** if S(x) is a transient macrostate.

*CRN condensation algorithm.* While coarse-graining yields the species $\hat{C}$ of a condensed CRN, we still need to find condensed reactions $\hat{R}$ between them. For each slow reaction in the detailed CRN, and for each way that the (often transient) products could reach resting states again by following fast reactions, we will introduce a condensed reaction between the resting macrostates corresponding to the reactants and the resting macrostates that were eventually reached (even if the resulting resting states are no different from the starting ones). There may be several distinct slow reactions in the detailed CRN that correspond to interactions between the same resting macrostates (just different microstates) and yield the same eventual products.

To make this construction precise, we introduce the **set of fates** of a single complex F(x), which, intuitively, describes all combinations of resting complexes that could emerge from the complex *x* after all fast reactions have gone to completion. (There may be more than one possibility, depending on which reactions take place first, see figure 5.) We can also calculate the set of fates of a multiset of complexes, which can be used to define the set of fates of a reaction R(r)=F(X), where *X* are the products of a reaction *r* and the extension of F to multisets is as defined below. The set of condensed reactions can now be computed. For each slow reaction in the detailed CRN, we convert each reactant (which will be a resting complex) to its corresponding resting macrostate, and then we produce a condensed reaction for each possible fate. The formal derivation is shown below.

*Cartesian sum.* We will use blackboard-bold braces ⦃⦄ to represent multisets and normal braces { } to represent sets. Let *A* and *B* be sets of multisets; then we write the **Cartesian product** as *A* × *B* = {(*a*, *b*) : *a* ∈ *A*, *b* ∈ *B*}. The **Cartesian sum**, by contrast, is an operation that sums each of the individual pairs of the Cartesian product, and returns a set of all the sums i.e.

3.1 A⊕B={a+b : a∈A,b∈B}.

The result is, therefore, a *set of multisets*. The Cartesian sum is associative and commutative, such that we can write ${⨁}_{B_{i}\in B}B_{i}$ to represent $B_{1}⊕B_{2}⊕\cdots$ for all $B_{i}\in B$.

**Definition 3.1.**

A **fate** *F* of a complex *x* is a multiset of possible resting macrostates, reachable from *x* by fast reactions.

For example, if complex *x* is a dimer that can decompose into two identical *resting* complexes: *x* → *y* + *y*, then ⦃S(y),S(y)⦄ is a fate of *x*. A complex *x* may have many fates, and all complexes must have at least one fate. We will denote the **set of fates** by F(x). For example, F(x)={⦃S(x)⦄} if and only if S(x) is a resting macrostate. Fates for different complexes are independent, hence, the set of fates of multiple complexes X=⦃$x_{1},x_{2},\ldots \;$⦄, is the set of all possible combinations of the fates of *x*_1_, *x*_2_, etc. Therefore, F(X) is given by the Cartesian sum

3.2 $$F(X)=\underset{x\in X}{⨁}F(x)=F(x_{1})⊕F(x_{2})⊕\cdots$$

We now define the set of fates for a detailed reaction *r* = (*A*, *B*) as R(r)=F(B), where B=⦃$b_{1},b_{2},\ldots b_{n}$⦄ are the products of the reaction *r*

3.3 $$R(r)=F(B)=\underset{b\in B}{⨁}F(b)=F(b_{1})⊕F(b_{2})⊕\cdots ⊕F(b_{n}).$$

Finally, let *R*_out_(*S*) be the set of fast reactions leaving some transient macrostate *S*, then we can provide an expression for F(x) in terms of a recursion

3.4 $$F(x)=\left\{ \begin{array}{cc} \{\{\;|\;S(x)\;|\;\}\}\; & \text{if}\;S(x)\;\text{is a resting macrostate}\;\\ \underset{r\in R_{\mathrm{out}}(S(x))}{⋃}R(r)\; & \text{otherwise.}\;\\ \; \end{array}\right.$$

Equation (3.4) can be evaluated in finite time: consider the graph *Γ*′, where the nodes are SCC of *Γ*, and there is a directed edge between nodes if there is a reaction with arity (1, *n* > 1). *Γ*′ is a directed acyclic graph, as all cycles were condensed into single nodes. That means, if we start with some arbitrary transient complex *x*, the recursion can be evaluated by a depth-first traversal of *Γ*′, starting from *x*; since *Γ*′ is acyclic, each branch of the depth-first traversal will terminate at a leaf of *Γ*′, i.e. a resting complex for which F(x) is trivial.

With F(x) capturing all of the information about the fast reactions in which *x* participates, we can easily calculate the set of condensed reactions. The condensed reaction network $\hat{G}=(\hat{C},\hat{R})$ has $\hat{C}$ being the set of resting macrostates; we build $\hat{R}$ as follows: for each slow reaction *r* = (*A*, *B*) ∈ *R*_*s*_, with S(A)=⦃$S(a_{i})\;:\;a_{i}\in A$⦄, then for each fate F∈R(r), we add a condensed reaction (S(A),F) to $\hat{R}$. Some of the condensed reactions constructed this way may be **unproductive**, in the sense that the multiset of products is identical to the multiset of reactants. Such unproductive reactions are omitted from $\hat{R}$; the detailed CRN reactions that gave rise to the unproductive reactions will, however, be considered when rates are assigned to condensed reactions. Pseudocode for the CRN condensation algorithm can be found in electronic supplementary material, §1.2. In appendix §B, we present theorems justifying the choice of this algorithm.

## 4. Approximate kinetics

To support simulation and rate-dependent separation of timescales, we introduce a model for approximating the rate constant for all detailed reactions supported by Peppercorn. We also present a model for approximating the rate of each condensed reaction to accompany our algorithm for reaction condensation. Thus, all CRN generated by Peppercorn can be transferred directly to ODE or stochastic kinetic simulation packages for further analysis. In §5, we use the script **Pilsimulator**, which is also provided with the peppercornenumerator library to simulate Peppercorn’s standard output format (PIL) using the ODE solver from **SciPy** [61]. We provide a brief sketch of the detailed and condensed reaction kinetic models here; see appendix §C for details.

### 4.1. Approximate detailed reaction kinetics

Peppercorn uses empirical domain-level reaction rates derived from selected DNA strand displacement and other DNA biophysics experiments. The bind21 and open reaction formulae are based on studies of the kinetics and thermodynamics of duplex hybridization and dissociation [25–27,62]; the bind11 reaction formulae are based on studies of zipping [26,63,64], bubble closing [32], and hairpin loop closing [31,33,36,39,65]; the three-way-fw and three-way-bw reaction formulae are based on studies of toehold-mediated three-way strand displacement [34,38]; the four-way reaction formulae are based studies of toehold-mediated four-way branch migration [28,29,37]; and the treatment of remote toeholds [35] is based on the loop closing probabilities from the biophysics of hairpin closing. The domain-level reaction rate constants used here assume perfect Watson–Crick complementarity of domains, experimental conditions at 25°C and 10 mM Mg^2+^, as well as ‘well designed’ sequences that minimize unintended internal secondary structure and minimize unintended base-pairing interactions between non-complementary domains. This is often achieved by using a three-letter nucleotide alphabet (A, T, C) or (A, T, G) for domains and their complements, therefore avoiding unintended, stable G·C pairs within a domain. Under these assumptions, we calculate rates that only depend on the length of involved domains and the type of a reaction: unimolecular binding, bimolecular binding, opening, as well as proximal or remote three-way or four-way branch migration. See appendix §C.1 for details.

### 4.2. Derivation of condensed reaction kinetics

The rate of each condensed reaction is derived from the detailed reaction rates. In simple cases, for example when resting macrostates each consist of a unique resting complex, each condensed reaction $\hat{r}=(\;\hat{A},\hat{B})$ is derived from one slow reaction *r* = (*A*, *B*) in the detailed CRN and one of its fates. Reactant resting complexes in *A* are the reactant resting macrostates in $\;\hat{A}$, and $\hat{B}$ is a multiset of resting macrostates reachable from the products *B* of the detailed reaction *r* via fast reactions. However, in the general case, a single condensed reaction may correspond to multiple pathways in the detailed network that have an equivalent end result. For example, in figure 4, there are two detailed bimolecular reactions that involve reactants from resting macrostates A and B and produce a transient complex in the central transient macrostate, which may then break down into some combination of C, D, E and F. The overall rate of e.g. A + B → E + F must sum the rates for all the possible detailed pathways. Thus, in general, each condensed reaction $\hat{r}=(\;\hat{A},\hat{B})$ between multisets of resting macrostates $\;\hat{A}$ and $\hat{B}$, is derived from all *slow* reactions *r* = (*A*, *B*) between multisets of complexes *A* and *B*, where *A* contains one resting complex from each resting macrostate in $\;\hat{A}$, and *B* are product complexes that can reach a multiset of resting complexes *X* via *fast* reactions, where *X* contains one resting complex from each resting macrostate in $\hat{B}$.

The rate of a condensed reaction depends on three quantities: (1) The probability that each resting macrostate is in a configuration that permits the underlying slow reaction *r* to occur; that is, the probability that each resting macrostate in $\;\hat{A}$ is transiently in the *microstate* corresponding to the reactant in *A* of *r*. (2) The rate of the underlying slow detailed reaction *r*. (3) The probability that the products *B* of *r* decay to the multiset of resting macrostates described by $\hat{B}$. This resulting rate is summed over all detailed reactions *r* that correspond to the given condensed reaction. We model each resting and transient macrostate as a continuous-time Markov chain (CTMC) between microstates, with detailed reactions representing possible transitions between microstates and transition probabilities given by the detailed reaction rates. From here, the stationary distribution of each resting macrostate can be calculated to give (1), the detailed CRN directly gives (2), and the decay probabilities of each transient macrostate (treating outgoing fast reactions as absorbing states) can be calculated to give (3). Our algorithm to calculate decay probabilities mirrors the algorithm for CRN condensation, so that the condensed reaction rates can be calculated alongside the condensed reactions. See appendix §C.2 for details.

### 4.3. Comparing detailed and condensed reaction kinetics

**Figure 6.**
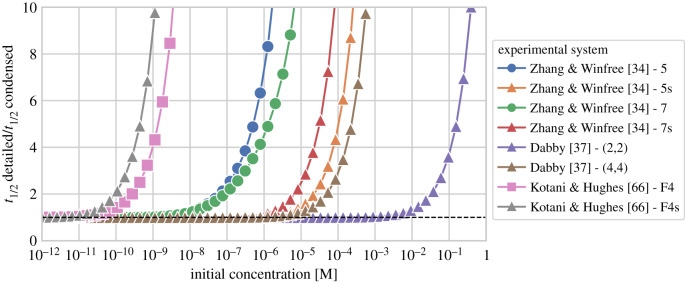
Comparison of simulation results for detailed and condensed domain-level reaction networks at increasing initial concentrations. We calculate the time point when a product species reaches 50% of its final concentration in the detailed and condensed network, and plot the ratio of *t*_1/2_ for detailed and condensed systems. At low initial concentrations (all initial complexes less than or equal to 10 pM), this ratio is close to 1 in all our examples, which confirms that bimolecular steps are rate-limiting. All examples shown here are taken from literature [34,37,66] and will be explained in more detail in §5 (figures 7 and 8). Triangles denote single condensed reactions: Zhang & Winfree [34] - 5s and - 7s are single condensed three-way strand displacement reactions with a 5 nt and 7 nt toehold, respectively. Dabby [37] - (2,2) and - (4,4) are each single condensed four-way strand displacement reactions with two 2 nt and 4 nt toeholds. Simulations start to differ between 1 μM and 1 mM initial concentrations of complexes. Kotani & Hughes [66] - F4s is a slow condensed reaction isolated from a complex autocatalytic DSD system ({I5 + S6 → P2 + P8 + P9 + C} cf. figure 8). The detailed reaction pathway requires multiple four-way branch migration reactions to succeed, and can only be considered fast at concentrations below 10 pM. Circles denote two condensed reactions: Zhang & Winfree [34] - 5 and - 7 show the original experimental setup to measure reaction rates, which involves a separate reporter reaction. The full detailed network contains an unproductive toehold interaction between substrate and reporter that slows down the system at concentrations above 10 nM. When using rate-independent enumeration, this effect (called toehold occlusion) can only be observed in the detailed CRN. Squares show a complex system of many reactions: Kotani & Hughes [66] - F4 is an autocatalytic system, which contains slow four-way branch migration reactions (such as in the single condensed reaction discussed earlier). As a consequence, already at low concentrations the rate-limiting steps are not always bimolecular, and we will use rate-dependent enumeration and condensation when analysing this system.

Condensation allows for analysis of some CRNs for which the detailed representation is too large. For example, ODE simulations of the Seesaw square-root circuit shown in figure 10 are only feasible using the condensed reaction network. The theorems in appendix §B state that all transition pathways between resting complexes in the detailed CRN are preserved as condensed reactions between resting macrostates in the condensed CRN. Thus, how well the dynamics of a condensed reaction network approximates the detailed network (our ground truth) ultimately depends on the timescale separation argument. Because all fast reactions are unimolecular, the mean residence time in a transient state, which is missing in the condensed model, is a (concentration independent) constant given by all outgoing rates. The derivations in appendix §C provide rates for condensed reactions that guarantee simulations of detailed and condensed networks to match exactly in the limit of low concentrations, where bimolecular reactions are always much slower than unimolecular reactions. Simulations of detailed and condensed networks confirm this expectation, and further illustrate that the point at which deviations arise, as the concentrations increase, may vary considerably from system to system (figure 6). Intuitively, the condensed reaction rates may fail to accurately represent the detailed system when, in the detailed system, the rates of bimolecular reactions approach those of rate-limiting unimolecular reactions. Using rate-dependent semantics, which include additional unimolecular reactions based on *k*_fast_ and *k*_slow_, may therefore extend the range of concentrations for which the condensed network is accurate. Of particular importance in some systems are the unproductive reactions, such as ‘toehold occlusion’ [4,7], where two species bind temporarily before falling apart again into the original species. Without rate-dependent semantics, such reactions will be omitted from the condensed network, but at high enough concentrations, they will sequester a substantial fraction of molecules in the detailed network. When examining the subnetwork of the detailed system that corresponds to just a *single* condensed reaction, accuracy may be preserved to higher concentrations. The comparison of detailed and condensed semantics will be explored more in figure 8.

**Figure 7.**
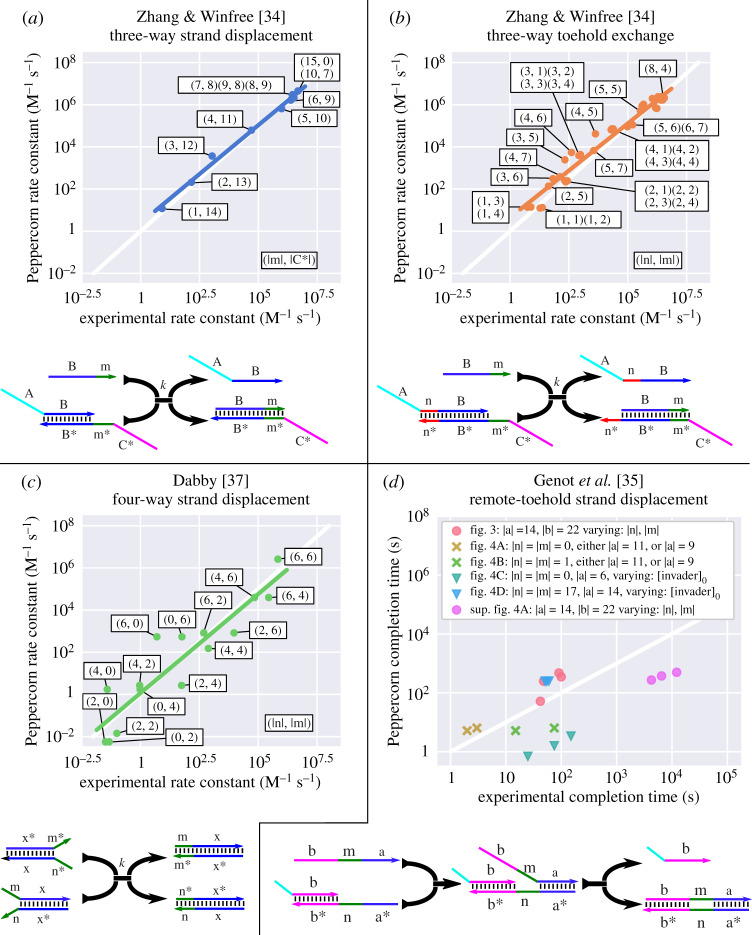
Comparison of Peppercorn’s condensed reaction rates with experimentally derived reaction rate constants; corresponding drawings below illustrate the design of these investigated systems. (*a*–*c*) Plots compare a range of different toehold lengths and branch-migration domain lengths for classic three-way strand displacement [34], three-way toehold exchange [34], and four-way strand displacement [37], respectively. Regression lines show the correspondence of model and experimental data over approximately nine orders of magnitude. An annotation is provided for selected points, corresponding to the lengths of relevant domains in the drawings below. (*d*) Comparing 30% system completion times for proximal and remote toehold experiments measured by Genot *et al*. [35] with Peppercorn predictions. Experimental data are taken from six figures in [35] (fig. 3, fig. 4A, fig. 4B, fig. 4C, fig. 4D and sup. fig. 4A). There are three types of experiments (indicated by different marker shapes): fig. 3, sup. fig. 4A [35] (circles) compare remote-toehold three-way strand displacement with variable length of the spacer region between toehold and branch-migration domain, using slightly different experimental set-ups; in fig. 3 [35], a fluorophore/quencher pair is attached directly to the displaced strand, while in sup. fig. 4A [35] a separate reporter reaction occurs. fig. 4A [35] measures kinetics of a 9 or 11 nt proximal toehold (where |*n*| = |*m*| = 0), compared to a remote toehold in fig. 4B [35] (where a 1 nt spacer region was introduced; crosses). fig. 4C [35] analyses the sensitivity of proximal 6 nt toehold to different initial concentrations of the invader strand ([invader]_0_ = 66 nM, 145 nM, 330 nM), compared to a remote setting (fig. 4D [35]), where a 14 nt toehold combined with a 17 nt spacer was used (triangles). Peppercorn’s model confirms the observation that the remote toehold makes the overall reaction rate insensitive to concentration changes; the three data points overlap. Runtime for enumeration (*T*_*E*_) and condensation (*T*_*C*_) on a PC (i5-4300U CPU @ 1.90 GHz): (*a*,*b*) *T*_*E*_ + *T*_*C*_ = 3.86 s, (*c*) *T*_*E*_ + *T*_*C*_ = 878 ms, (*d*) *T*_*E*_ + *T*_*C*_ = 347 ms.

**Figure 8.**
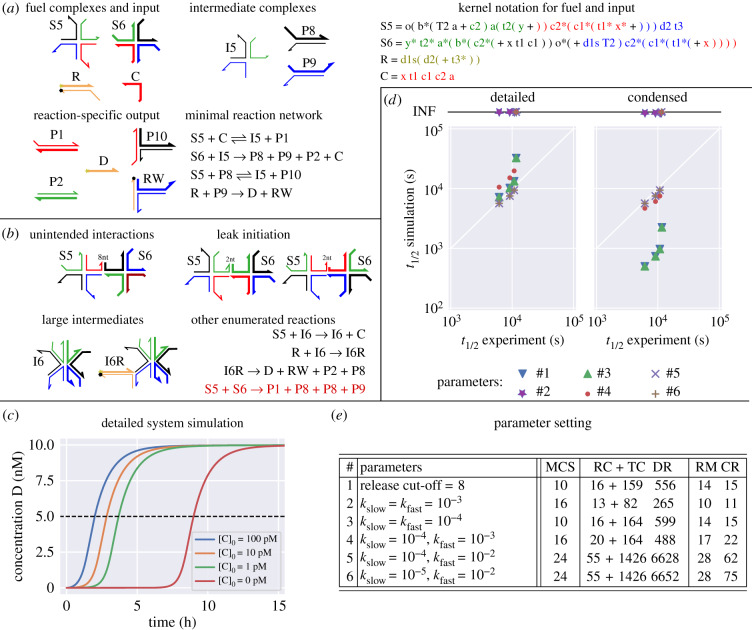
Autocatalytic DNA strand displacement from Kotani & Hughes [66]. A system with diverse reaction pathways involving three-way, four-way, and remote-toehold branch migration. (*a*) An overview of the intended system. Six reactions (two reversible, two irreversible) perform autocatalytic amplification of C. The colours of complexes are chosen to indicate which strands of the fuel complexes will eventually hybridize. R is the reporter complex with a fluorophore (yellow star) and quencher (black dot) on one side. The top strand of the reporter is called Dye (D) and used to track the production of catalyst C. Kernel strings using the same colour scheme are shown for all initially present complexes. Despite a difference in colour the unpaired part of P8 has the same sequence as C and thus can act as catalyst. (*b*) Examples of unintended reactions, large intermediate complexes, and leak reactions. The leak pathway (bottom, red) produces products without presence of the catalyst. (*c*) A simulation of the experimental setup with initial conditions ${\left[S5\right]}_{0}={\left[S6\right]}_{0}=10$ nM and $[{R]}_{0}=20\;\text{nM}$ shows trajectories of the Dye species D for four initial conditions of catalyst C. The system was enumerated using rate-independent semantics (i.e. parameter setting **#1** in the table). Colour scheme corresponds to fig. 4 in Kotani & Hughes [66], which shows experimental data. Note that this enumeration semantics includes the leak reaction, triggered without the presence of C. The dashed line marks the threshold to calculate the **50%-completion time** for comparison with experimental data. (*d*) The plot compares 50%-completion times (*t*_1/2_) for six different enumeration semantics shown in (*e*), each with the four initial conditions simulated in (*c*). Enumeration using setting **#2** does not yield the fluorescent product species, settings **#4, #5, #6** do not return the leak pathway; the corresponding simulation trajectories (with [C]_0_ = 0) never reach 50%-completion time (*INF*). (*e*) The table gives different enumeration parameters that have been tested: release cut-off, *k*_slow_ and *k*_fast_, and their effects in terms of **maximum complex size (MCS)**, numbers of **resting complexes (RC)**, **transient complexes (TC)** and **detailed reactions (DR)** for the detailed CRN and numbers of **resting macrostates (RM)** and **condensed reactions (CR)** for the condensed model. Runtime for enumeration (*T*_*E*_) and condensation (*T*_*C*_) on a PC (i5-4300U CPU @ 1.90 GHz): (*d*) detailed enumeration: *T*_*E*_ = 1 min 19 s, (*d*) condensed enumeration: *T*_*E*_ + *T*_*C*_ = 1 min 59 s.

## 5. Case studies

We now compare Peppercorn’s rate model with experimental data. First, we present the correspondence to data that were used to parameterize our present rate model (basic three-way and four-way strand displacement reaction pathways, figure 7*a*–*c*), then we compare our simulations against a broad range of different experimental case studies. We consider a less than 10-fold difference in reaction rates or completion times to be satisfactory, given the limitations and simplifying assumptions of our model. This is achieved for most of the simple cases and some of the more complex cases studied, but may be dependent upon choices for the enumerator parameters and settings, as discussed below. Code to reproduce the following plots is available on the peppercornenumerator Git repository [42], and raw data are given in electronic supplementary material, §2.

**Figure 9.**
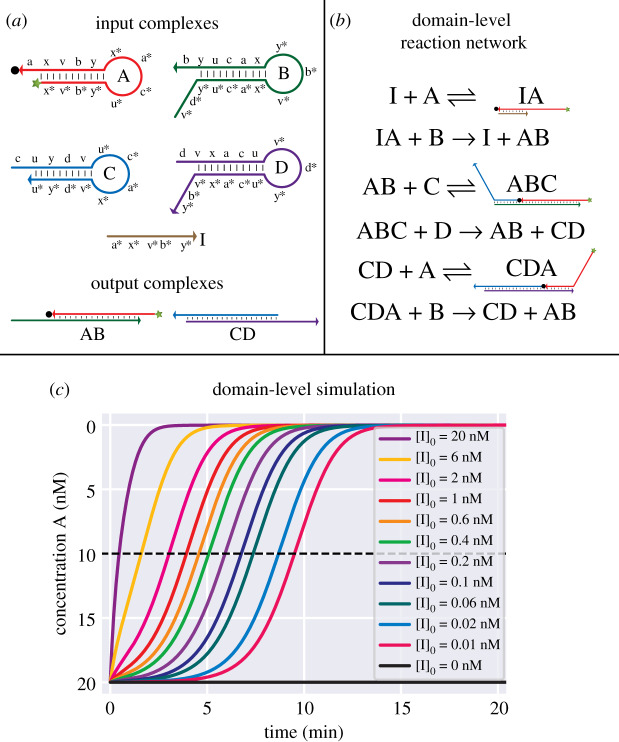
Cross-catalytic DNA hairpin system [55]. (*a*) The input complexes A, B, C, D, I, and output complexes AB, CD. Species A has a fluorophore/quencher pair attached (shown as star and dot in the figure), which is initially separated by about 6 nt (quenched), and presumably much further apart (not quenched) after the initiator starts invading at the helix end. (*b*) Peppercorn enumerates nine condensed reactions: three reversible and three irreversible. The initiator I starts a catalytic process where A and B are consumed to form AB; AB itself is a catalyst to produce CD, and CD is a catalyst to produce AB. The three ‘reverse’ reactions in this system are unimolecular remote-toehold interactions. (*c*) Simulations of the enumerated system at different concentrations of the initiator I. The trajectories start at [A]_0_ = [B]_0_ = [C]_0_ = [D]_0_ = 20 nM and show the decrease of species A over time. The dashed black line marks the 50%-completion time, which was used to compare with experimental data in figure 11. Colour scheme corresponds to fig. 3 in Yin *et al*. [55], which shows experimental data. Runtime for enumeration (*T*_*E*_) and condensation (*T*_*C*_) on a PC (i5-4300U CPU @ 1.90GHz): (*c*) *T*_*E*_ + *T*_*C*_ = 274 ms.

**Figure 10.**
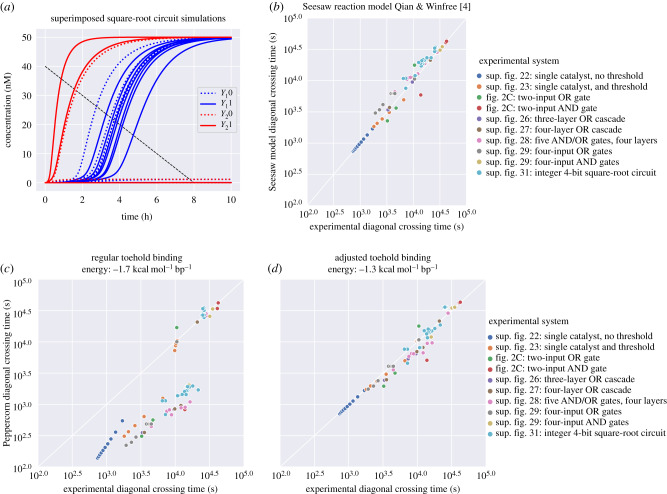
Enumeration and simulation of DNA strand displacement circuits using the Seesaw architecture [4]. (*a*) Superimposed simulations of the largest circuit, which computes the floor of the square root of a 4-bit binary number. *Y*_1_0, *Y*_1_1, *Y*_2_0, *Y*_2_1 represent the dual-rail implementation of the 2-bit binary output. The full trajectory for each of the 16 possible input combinations is shown. The **diagonal-crossing time** metric used in this and subsequent plots enables us to compare OFF signals, which remain at low concentration and are inherently relevant for dual-rail systems. We choose the endpoints of the diagonal at the maximum clearly visible reference point of experimental data plots (see electronic supplementary material, §2). Enumeration and simulation settings correspond to those explained for (d). (*b*) Comparison of the Seesaw compiler simulation model with experimental data, both derived from Qian & Winfree [4] and using the diagonal-crossing time metric described above. (*c*) Comparison of Peppercorn enumeration with the same experimental results using condensed, rate-dependent semantics (*k*_slow_ = 0.01 s^−1^, *k*_fast_ = 1 s^−1^). Differences between OFF trajectories (comparatively slower) are small, but differences on ON trajectories can be strong, as simulated systems are much faster than the experimental results. (*d*) Comparison of Peppercorn enumeration with experimental results after the toehold binding free energy has been changed to −1.3 kcal mol^−1^ bp^−1^. This slows down ON trajectories sufficiently to provide similar-quality predictions of completion time as the Seesaw compiler itself. Runtime for enumeration (*T*_*E*_) and condensation (*T*_*C*_) on a PC (i5-4300U CPU @ 1.90GHz): For of all systems except square-root circuit: *T*_*E*_ = 7.42 s, *T*_*E*_ + *T*_*C*_ = 19 s. Square-root circuit: *T*_*E*_ = 1 min 15 s, *T*_*E*_ + *T*_*C*_ = 8 min 1 s.

Where possible (e.g. when only a single condensed reaction is involved) we compare experimentally derived reaction rate constants directly (see figure 7*a*–*c*); otherwise (e.g. when the behaviour of a system of reactions is measured) we switch between two different metrics, both of which compare experimentally observed strand displacement dynamics against enumeration and ODE system simulation using a single time point, rather than the full course of the trajectory. The first metric is called **50%-completion time**; it compares the time where the reporter species of experiment and simulation reach 50% of total concentration (e.g. figure 9*c*). This measurement provides qualitative feedback only for ‘fast’ systems that actually reach 50% on the timescale of an experiment. In order to capture both fast and slow systems, we use a second metric, called **diagonal-crossing time**, which compares the time points where experiment and simulation cross a chosen diagonal line that connects the *x* and *y* axes at the maximum clearly visible ticks from experimental data plots (e.g. figure 10*a*). Data points were extracted using the **WebPlotDigitizer** tool [67]; details on which points of reference were used can be found in electronic supplementary material, §2. In §5.5, we show that the choice of metric does not influence the qualitative correspondence between Peppercorn’s predictions and the experimental data.

In the following section, we say a reaction is *intended* if the authors used this reaction to describe a strand displacement system, or it is clear from context that the reaction is part of the desired circuit behaviour. By contrast, a reaction is *unintended* if it was not explicitly presented by the authors of a system and it is not essential for the function of the system, but it does not change the logic of the system. We use the term *leak* to denote a reaction that changes the logical behaviour of the system, e.g. by producing output without the presence of input signals, or by taking shortcuts from input to output without producing the proper intermediate signals.

### 5.1. Condensed reaction rates of basic strand displacement

As mentioned above, our rate model for detailed reactions was developed based on studies of basic biophysical processes, with some parameters tuned to match phenomena that are especially important for dynamic DNA nanotechnology. We therefore begin our assessment of the kinetics model by examining experimental systems that correspond to a single condensed reaction, such that the rate constants can be directly compared. In figure 7*a*–*c*, we compare condensed reaction rates for **toehold-mediated three-way strand displacement** [34], **three-way toehold exchange** [34] and **toehold-mediated four-way strand displacement** [37] with experimental data. Rate constants predicted by our model correspond to experimentally observed rate constants over nine orders of magnitude. Among these experiments, four-way strand displacement reaction rates show the largest variability. The data from figure 7*a*–*c* were used during the development of the model, and therefore the prediction errors are best considered as part of the ‘training error’, in the parlance of machine learning. See electronic supplementary material, §2 for raw data of reaction rates.

A comparison of our rate model with reaction times for **proximal and remote toehold-mediated three-way strand displacement** [35] can be seen in figure 7*d*. Some experiments here use long toeholds and slow remote branch-migration reactions, which are incompatible with the timescale separation of the rate-independent model. Thus, we use rate-dependent enumeration with $k_{\mathrm{slow}}=10^{-6}\;{\text{s}}^{-1}$ and *k*_fast_ = 20 s^−1^ such that all relevant detailed reactions are considered slow (e.g. dissociation of 14 nt toeholds). These data were not considered during the development of the kinetic model, and therefore may be considered as our first assessment of the ‘testing error’. For example, experiments suggest a much larger difference in remote branch migration reaction rates when a 1 nt spacer is introduced, which Peppercorn’s current rate model does not predict.

### 5.2. Autocatalytic DNA strand displacement system

Autocatalytic feedback systems are particularly difficult to control and to simulate reliably, as small errors may be exponentially amplified. This can be seen in a system presented by Kotani & Hughes [66]. It involves large complexes with up to 24 individual strands and a diverse set of detailed reactions: three-way branch migration, four-way branch migration and remote-toehold three-way branch migration. Figure 8 provides an overview of the system. There are six intended reactions (two reversible, two irreversible), which perform autocatalytic amplification of catalyst complex C. We depict a simplified condensed CRN in figure 8*a*, which differs from the actual enumeration results (figure 8*b*,*e*), as discussed below.

*Enumeration semantics.* The choice of enumeration semantics for this system has interesting effects on predicted dynamics. The analysis is tedious, but understanding the subtle consequences of different semantics can be rewarding when newly designed systems are evaluated prior to experimental testing. In figure 8*e*, we summarize the different enumeration parameters and compare properties of the enumerated detailed and condensed reaction networks. figure 8*d* shows corresponding simulated 50%-completion times in comparison with experimental results.

**Setting #1:** A rate-independent enumeration. We have to set the option release-cutoff to 8 nt or higher, as there is at least one unintended 8 nt bind reaction (see figure 8*b*), and this binding has to be reversible in order to avoid predicting implausible polymers. The 50%-completion time of the condensed reaction network is orders of magnitude less than that of the detailed reaction network (figure 8*c*), indicating that there are time-consuming reaction pathways in the detailed network that had been assumed to be instantaneous during rate condensation.

**Setting #2:** A rate-dependent enumeration with *k*_slow_ = *k*_fast_ = 10^−3^ s^−1^. In comparison, the rate of an 8 nt open reaction has *k*_uni_ ≈ 6 × 10^−3^ s^−1^, and thus all previously mentioned unintended 8 nt bindings are reversible by fast opening reactions. It turns out that starting enumeration with species S5, S6 and C does not yield the product D, as important four-way branch migration reactions are slower than *k*_slow_ = 0.001 s^−1^, and therefore considered negligible.

**Setting #3:** A rate-dependent enumeration with *k*_slow_ = *k*_fast_ = 10^−4^ s^−1^ (corresponding to a release cut-off between 9 and 10 nt) includes all important four-way branch migration reactions and yields the same detailed and condensed simulation results as in the rate-independent model.

**Setting #4:** A rate-dependent enumeration with *k*_slow_ = 10^−4^ s^−1^ and *k*_fast_ = 10^−3^ s^−1^. 50%-completion times of the condensed network are longer due to the rate constants of slow unimolecular reactions. This results in similar predictions for condensed and detailed networks, and a better fit with experimental results. The maximum observed complex size increases, as slow unimolecular reactions cause an additional stable intermediate state I6R (figure 8*b*). Two copies of I6R can engage in a transient interaction of 16 strands. Interestingly, with four-way branch migration being a slow reaction, we do not observe the *leak* pathway {S5 + S5 → P1 + P2 + P8 + P9} (figure 8*b*) because dissociation of a fast 2-nt toehold will always occur before the slow branch migration step.

**Setting #5:** A rate-dependent enumeration with *k*_slow_ = 10^−4^ s^−1^ and *k*_fast_ = 10^−2^ s^−1^. This assigns more unimolecular reactions into the slow regime, pushing results closer to the experimentally observed results. However, this also increases the size of detailed reaction network more than 10-fold.

**Setting #6:** A rate-dependent enumeration with *k*_slow_ = 10^−5^ s^−1^ and *k*_fast_ = 10^−2^ s^−1^. More low-probability reaction pathways are included in the detailed and condensed reaction network, but have no observable effect on 50%-completion times.

The bottom line here is that it remains important to explore different semantics and parameter settings in order to understand and appreciate the possible behaviours of a system, since we cannot at this time recommend a universally ‘best’ setting. Here, we might consider setting #4 to be a good compromise of simplicity and accuracy. Note that while it was nice that Peppercorn identified a (real) leak pathway with settings #1 and #3, in general we do not expect Peppercorn to automatically detect leak pathways because in most real systems these appear via zero-toehold strand displacement, which is not a part of the current Peppercorn enumeration semantics.

### 5.3. Cross-catalytic hairpin system

This case study analyses a cross-catalytic system that uses only DNA hairpin structures [55] and a single stranded initiator. The system is designed on the domain level using typical domain-level reaction pathways, but it is not a ‘classic’ DNA strand displacement system. For example, it does not use dedicated fuel complexes to translate input to output, and there are no condensed toehold-mediated strand displacement reactions. Instead, there are reaction pathways that require cooperative binding to form product complexes. Two properties of this system require specific enumeration semantics: first, all domains are toehold length, so the system can only be enumerated using max-helix semantics. Second, all bimolecular reactions are fully reversible via unimolecular steps, so we need to use the rate-dependent model to classify critical unimolecular reactions as slow.

**Figure 11.**
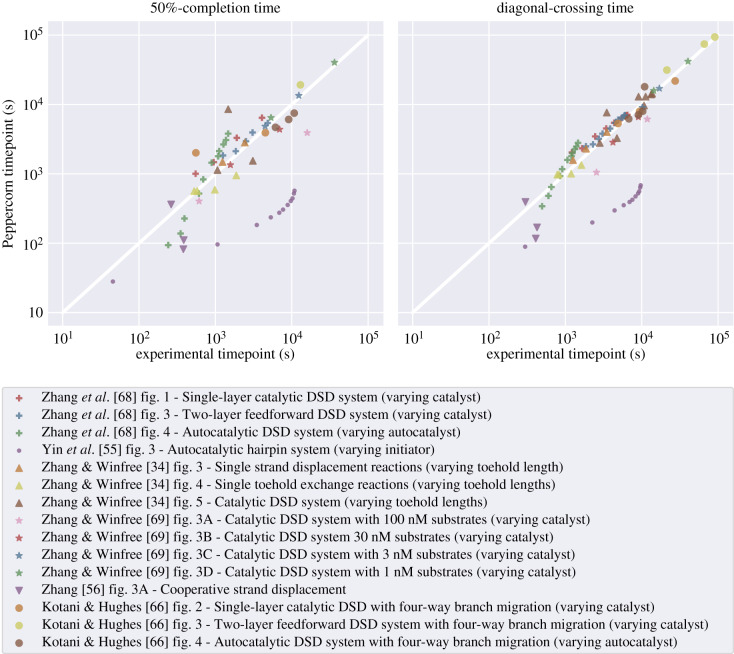
Simulated condensed DNA systems in comparison with fluorescence measurements from experimental data. We simulate data from six selected publications with a variety of DNA reaction networks [34,55,56,66,68,69], and compare both 50%-completion time (58 data points), and diagonal-crossing time (77 data points). Yin *et al*. [55] fig. 3, Zhang [56] fig. 3A and Kotani & Hughes [66] fig. 4 use rate-dependent enumeration, all others use rate-independent enumeration. Runtime for enumeration (*T*_*E*_) and condensation (*T*_*C*_) on a PC (i5-4300U CPU @ 1.90GHz) for all systems: *T*_*E*_ + *T*_*C*_ = 3.17 s.

**Figure 12.**
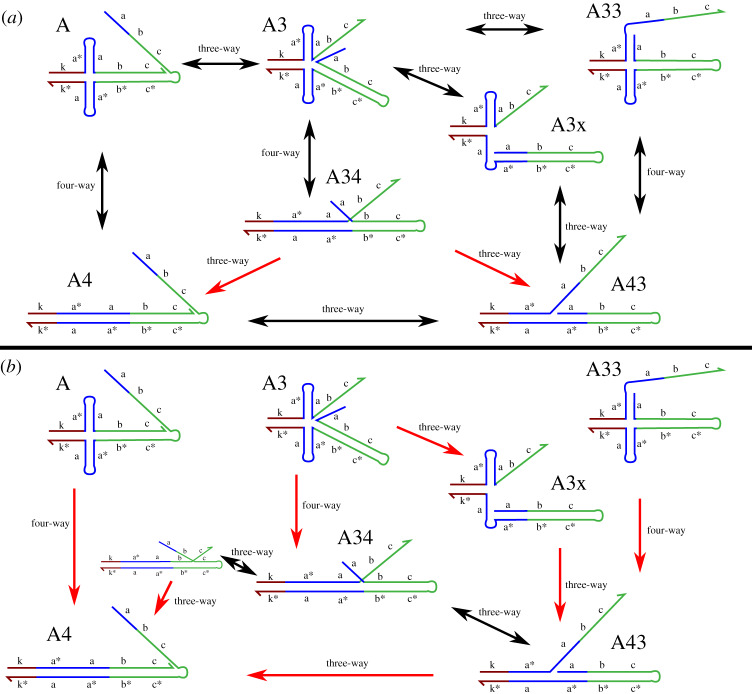
Irreversible reactions as a consequence of max-helix and reject-remote semantics. (*a*) Max-helix semantics. Domains b and c are always part of the same max-helix move, domain a can sometimes, but not always be combined with b and c. This competition of different rules can lead to irreversible reactions, e.g. {A34→A43,A34→A4}. However, note that the every single complex is still part of the same SCC, where every state is reachable from every other state. Also, if k were a toehold domain (not shown), then only complexes A, A3, A33 and A3x would have an outgoing reaction opening that toehold, but the SCC would contain a copy of every complex with an open toehold, connected via the shown three-way and four-way branch migration reactions. Also, if k is a toehold, A3 and A3x would split into two complexes, and the shown SCC would become a transient macrostate. (*b*) Reject-remote semantics. Only proximal toehold branch migration reactions are allowed, which leads to a highly disconnected state space. Note an extra unnamed intermediate state on the path {A34→A4} as we do not use max-helix semantics here.

The enumerated CRN using *k*_slow_ = 10^−5^ s^−1^ and *k*_fast_ = 0.1 s^−1^ is shown in figure 9. It has nine reactions, three of which are the catalytic formation of AB using the catalyst (or initiator) I. AB then can catalyse the formation of CD, which itself catalyses the reaction of AB. While Peppercorn predicts the correct CRN underlying the system, the qualitative fit of 50%-completion times is the worst across all case studies. A comparison of many case studies can be found in §5.5 (see figure 11). Presumably, the remote-toehold strand displacement mechanism to reverse dimerization is highly sequence dependent. Note that it is also possible to enumerate the system with the rate-independent model when disabling remote-toehold interactions. In that case, the enumerated CRN would be the same as the intended network presented by Yin *et al*. [55] or the one enumerated by VisualDSD as shown in Petersen *et al*. [18].

### 5.4. Seesaw DNA strand displacement architecture

The **Seesaw** architecture [4] was developed to implement scalable, multilayer, digital DNA circuits. Every DNA gate is equipped with threshold complexes (to filter low-concentration, unintended DNA input) and signal amplification complexes (to release the full amount of output, if the input signal exceeds the threshold). The systems can be designed using a compiler that translates a digital circuit into a specific set of DNA sequences, for which individual reaction rates have been measured experimentally. A strength of the Seesaw approach is that it provides rates for *intended* reaction pathways, as well as for selected *unintended* and *leak* pathways. All those reactions are taken into consideration when simulating Seesaw systems using the Seesaw compiler. See Qian & Winfree [4] or electronic supplementary material, §3 for details on Seesaw reaction semantics.

Peppercorn’s enumeration model is an independent approach that does not consider architecture-specific reaction pathways. After enumeration, Peppercorn’s detailed reaction network contains all the intended reactions and is generally consistent with the Seesaw compiler’s model (see electronic supplementary material, §3), with a few notable differences. (i) While the Seesaw compiler includes zero-toehold leaks, Peppercorn does not enumerate these reactions. Because the Seesaw experiments used a sequence design method that reduced leak rates, inclusion of these leak reactions with experimentally appropriate rate constants would not significantly change simulation results for the cases studies. (ii) Both models include unintended side reactions in which the universal toehold allows temporary binding of signal strands to unrelated gates, briefly preventing the toehold from being accessible for intended reactions (toehold occlusion). However, while the Seesaw compiler’s model makes an approximation to lump many of these unproductive reactions together for efficiency, the Peppercorn enumeration explicitly enumerates each, making simulation of large Seesaw networks infeasible. (iii) For the same reactions, rate constants in Peppercorn’s model, which uses generic biophysics, differ from those the Seesaw model, which was calibrated to experimental results. Most notable are the toehold occlusion reactions involving threshold gates: the Seesaw compiler uses a slower unbinding rate to account for additional spurious sequence-level interactions with domains neighbouring the universal toehold. (iv) Peppercorn identifies a gate–gate leak that acts via four-way branch migration, and which is not part of the Seesaw compiler model. This leak rate is so slow that it does not noticeably affect simulation results.

Figure 10 compares the Peppercorn and Seesaw models against experimental results for a variety of circuits from ref. [4]. Because for the largest of these circuits, the Peppercorn model can only be simulated after condensation, we use the condensed CRN for all simulations. Notably, the unproductive toehold occlusion reactions are pruned by condensation; these reactions slow down circuit function at high total strand concentrations, as occurs in large circuits—an effect that is therefore missing in the Peppercorn model. However, the Peppercorn model is faster than the Seesaw model even for small circuits (figure 10*b*,*c*), as a consequence of the different rate constants for the intended reactions. To demonstrate how slight modifications at the rate model can change the predictions, we provide a parameter (--dG-bp) to alter the binding free energy of an average base-pair, which affects the dependence of strand displacement reactions on the toehold length. After changing the sequence independent estimate for the toehold binding free energy from −1.7 kcal mol^−1^ bp^−1^ to −1.3 kcal mol^−1^ bp^−1^, the rates of intended reaction pathways are more comparable between the two models, and thus diagonal-crossing times in the simulations agree better with experimental results (figure 10*d*). Nonetheless, this adjustment should be understood as a phenomenological fit that is accounting for multiple factors, as the experimental slowdown is at least partially due to toehold occlusion [4], which is not being modelled in the condensed CRN. The individual trajectories predicted by Peppercorn and shown in figure 10*a* are therefore different than those provided by the Seesaw compiler (see electronic supplementary material, §3), even though the diagonal-crossing times are similar.

### 5.5. More systems

Figure 11 provides an overview of Peppercorn’s model performance using DNA strand displacement systems from a selection of additional publications [34,55,56,66,68,69]. All systems have been enumerated starting with the initially present complexes and then the condensed CRNs were simulated. We use both metrics: **50%-completion time** (e.g. figure 9*c*) and **diagonal-crossing time** (e.g. figure 10*a*) for comparison. The choice of metric can have an effect when comparing individual case studies, but does not qualitatively change the overall performance of Peppercorn’s predictions.

The autocatalytic hairpin system using remote toehold branch migration [55] is particularly difficult to simulate accurately. The experiment requires remote toehold strand displacement, for which the model is not sufficiently trained (see §5.3). On the other hand, both a paper where cooperative hybridization effects were investigated [56] and experiments that tested robustness of strand displacement using different nucleotide sequence designs are approximated reasonably well [69].

### 5.6. Conclusion

Taken together, the results demonstrate that (i) Peppercorn can enumerate a valid reaction network for all these systems, (ii) the enumerated reactions can provide a qualitatively good estimate for the experimental results, indicating that we choose individual reaction pathways with reasonable probabilities, (iii) the rate model cannot calculate the exact completion times for individual systems, as it does not compensate for the expected time spent before a reaction completes (see appendix §C), and (iv) obtaining useful results in some cases may require the user to select among the available enumeration semantics and parameters. For example, the default toehold length threshold for the rate-independent model (7 nt) is too stringent for many systems, and a first step would be to increase this parameter before exploring the rate-dependent model parameters that often provide more nuanced insights into modelling the system.

## 6. Discussion

We have presented (i) an enumeration algorithm for DSD reaction networks, (ii) a condensation algorithm to express a given CRN in terms of its overall slow reactions, (iii) an approximate rate model for DNA domain-level systems, and (iv) multiple case studies comparing our model against experimentally observed system dynamics. We have proven that condensation preserves the relevant properties of the detailed CRN—namely, that all transitions between resting sets are possible in the condensed CRN—and that the condensed CRN does not introduce spurious transitions, i.e. transitions that were not possible in the detailed CRN.

### 6.1. Related work

Early work from Nishikawa *et al*. [12] presents a DNA simulator that includes a joint enumeration and simulation model that uses ‘abstract bases’ analogous to our ‘domains.’ The six supported reaction types are hybridization, self-hybridization, denaturation, digestion, extension and ligation. Notably, the first three are a subset of Peppercorn’s supported reactions (bind21, bind11, and open), whereas the other three are enzymatic reactions to simulate a different class of experimental systems. Using tentative rate parameters for each reaction type, combinatorial explosion is controlled by only enumerating interactions between complexes during ODE simulation after they have exceeded some threshold concentration. An alternative strategy to cope with combinatorial explosion has been demonstrated by Kawamata *et al*. [13,14]. Their model enumerates and simulates a reaction network between *local structures*, not complexes, considering three types of reactions: bimolecular binding, dissociation and three-way branch migration. Enumeration of local structures corresponds to finding possible configurations of a single strand within different complexes. The state space of local structures can be finite for systems exhibiting genuine polymerization, such as hairpin chain reaction (HCR) [54], although the number of local structures still increases exponentially with the number of distinct strands in a system.

VisualDSD is well-known and well-developed software for enumeration and simulation of DNA strand displacement systems [15–18,20]. The enumeration semantics is based on a process calculus for modelling DNA strand displacement, originally allowing a restrictive class of secondary structures, e.g. no four-way junctions, hairpins, internal loops, or non-toehold single-stranded domains [15]. In the more recent versions (**LogicDSD**) [18,20], the language to express a ‘process’ is conceptually related to kernel notation for complexes, but does not prohibit pseudoknots. For example

x( u( y + ) ) t* = [< x!j u!k y > | < u*!k x*!j t* > ]

where the indices after ‘!’ (here j, k) indicate arbitrary paired domains, not restricted to nested structures. The rules as programmed by the user determine whether reactions involving pseudoknots, remote-toeholds or specific branch migrations are allowed, and these rules may be very specific to the investigated system. Rules are thereby often composed of multiple steps and can be conditional on yielding a specific product. By contrast, Peppercorn provides a modular set of single-step reaction semantics which are never conditional on following a desired reaction pathway. VisualDSD’s infinite reaction semantics treats all branch migration and open reactions as infinitely fast. This is similar to Peppercorn’s condensed semantics applied to a rate-independent enumeration; however VisualDSD does not provide an analogous formulation of rate-dependent condensation.

The default enumeration semantics of both VisualDSD and Peppercorn may be inappropriate for some systems; for instance, the enumeration may not yield the expected products, or expected reactions may be missed. However, the approach a user would take to address this behaviour is different for the two packages. In VisualDSD, to model systems that do not perform as expected, the user has to formulate additional abstract rules using the modelling language LogicDSD. By contrast, Peppercorn requires the user to adjust aspects of the biophysical model. This can be done via adjustments to domain lengths or toehold binding strength, or the system may require rate-dependent enumeration semantics. For example, in Petersen *et al*. [18], VisualDSD is used to enumerate the auto-catalytic hairpin system from Yin *et al*. [55] (see §5.3). Enumeration produces the expected reactions and complexes. However, additional reverse reactions—in which multistranded products dissociate and re-form the starting complexes—are not predicted, because they require a remote toehold. To find these reactions in VisualDSD, the user would need to write a rule in LogicDSD for remote toehold-mediated branch migration. Peppercorn identifies these reactions using default semantics. However, by default, these unimolecular reactions are expected to occur faster than downstream bimolecular reactions, so the expected final products are not found. In order to enumerate the expected complexes, one can either (i) use rate-dependent timescale separation as demonstrated in §5.3 or (ii) reduce the enumeration rule set by explicitly prohibiting remote toehold-mediated reactions with reject-remote semantics. As explained in appendix A, reject-remote semantics can miss biophysically important pathways and has to be used with caution. By contrast, option (i) yields a reaction network with all biophysically plausible reactions and provides insights about potential unintended pathways; specifically, these semantics reveal that the domain lengths (and hence the rates of the remote toehold-mediated reverse reactions) are critical to the proper functioning of the system—if these domains are too short, the reverse reactions will dominate, whereas if they are longer, the bimolecular reactions will have time to occur.

Other rule-based models developed for concisely representing combinatorial structures in systems biology and chemistry, e.g. BioNetGen [70], Kappa [71], MØD [72] could in principle be used for DNA systems. However, while we use general purpose pattern-matching reaction rules, those models require hard-coded rules for site-specific interactions which would have to be provided by the user for each system. A more in-depth description of this problem can be found in Petersen *et al*. [18] and in Mokhtar *et al*. [19], both of which present an encoding of DNA molecules into graphs and propose a set of graph rewrite rules applicable for DNA nanotechnology.

### 6.2. Future work

In conclusion, Peppercorn provides all relevant DSD reaction types within the domain of pseudoknot-free secondary structures, and thus can be used as an independent source to analyse the properties and dynamics of domain-level systems. However, future development should address several improvements for a more reliable and automated analysis: (i) refinements of the rate model, (ii) an automatic conversion from high-level experimental conditions to low-level enumeration semantics, (iii) refinements of the available enumeration semantics, and (iv) a combined enumeration and simulation mode for stochastic analysis of large systems.

*Rate model.* The rate model captures our understanding of DSD biophysics, and incorporates parameters to reproduce experimental results on single condensed reactions from Zhang & Winfree [34] and Dabby [37] (figure 7). We have shown that it is sufficient to get a qualitative understanding of domain-level system dynamics, but that individual system completion times are not reliable. That is not surprising, as the model has several limitations: (i) the parameters reflect a specific experimental set-up (temperature, ion concentrations), (ii) the parameters neglect nucleotide sequence variations, (iii) the model calculates the right probability of a successful reaction, but not necessarily the correct time spent in that reaction (see appendix §C).

Future development of a more sophisticated rate model may adjust for the expected time spent in a reaction pathway [41], may incorporate nucleotide sequence, temperature and buffer conditions, and may be optimized via systematic parameter inference to better match experimental measurements. Eventually, such a kinetic model can complement thermodynamic energy parameters [73], and provide deeper insights into fundamental principles of nucleic acid folding.

*Automatic choice of enumeration semantics.* The choice of parameters can be important and may require some knowledge about the experimental setup, e.g. when to use the rate-dependent model and which values for *k*_slow_ and *k*_fast_ are appropriate (see §5.2). Conversely, if specific semantics are required to find the intended reaction network from a set of initial species, then that has implications on how to choose an experimental setup. Yet it is an open problem to provide a high level interface that connects experimental conditions with particular parameters. For example, future versions (using a systematically trained kinetic rate model) may automate the choice of semantics, depending on initial species concentrations.

*Augmented enumeration semantics.* More fundamental changes in the enumeration semantics may be warranted. Our condensation algorithm for reducing the size of the enumerated CRN is justified with respect to the limit of low concentrations—an assumption that is also baked into the detailed enumeration semantics that ignores bimolecular interactions between transient species. As these assumptions do not hold for some cases of interest, it would be valuable to develop enumeration methods that are better adapted to the high concentration regime and to make use of CRN reduction methods that have been developed in more general contexts [74–76]. A further enhancement would be for Peppercorn to (at the user’s discretion) include bimolecular zero-toehold three-way and four-way branch migration reaction types, which would be valuable for exploring leak pathways in strand displacement circuits [77,78].

*Just-in-time simulation.* While our implementation exhaustively enumerates the full reaction network, other enumerators include a *just-in-time* simulation mode, which combines the enumeration and simulation processes. The algorithm generates a set of possible reactions among initial (or current) complexes and determines the products probabilistically for the next iteration. It is not clear how such a mode is compatible with the timescale separation approach used here, but the prospect of producing statistically correct samples from the time-evolution of the ensemble is appealing, as it would enable us to simulate, for example, systems with genuine polymerization.

## Supplementary Material

Supplemental Online Material

## Figures and Tables

**Figure 13.**
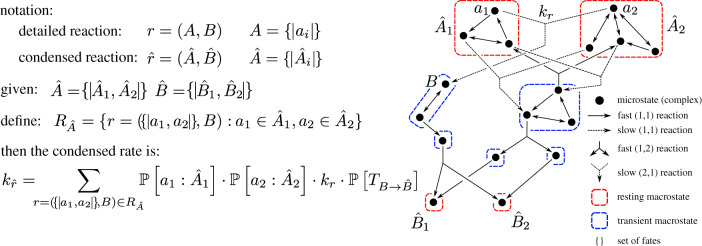
Reaction rate condensation summary. In order to calculate the condensed rate $\hat{r}=(\;\hat{A},\hat{B})$, we define $R_{\;\hat{A}}$ as the set of detailed reactions leaving $\;\hat{A}$ (to some species *B*). The condensed rate can be calculated from all reactions in $R_{\;\hat{A}}$, given that we have also calculated the steady-state probability of reactants, the detailed reaction rates and the probability that *B* decays into $\hat{B}$.

## Endnotes

1 Most directly, hairpin loops containing fewer than 3 unpaired bases are not sterically plausible; such cases may be effectively eliminated by assigning them a large energy or imposing a hard constraint. Although standard sequence-level models [22–24] strictly impose this constraint, Peppercorn does not impose any length constraint on hairpin loops at the domain level. For example, the hairpin x() is permitted. The rationale is that, at least for long enough domains, a real molecule would still form a hairpin, but with just a few intended base pairs omitted – this is not particularly worse than say long duplex domains where the real molecule would be likely to have a few base pairs breathing or fraying at the ends. It is sometimes assumed that other than the hairpin length constraint, non-pseudoknotted secondary structures guarantee the absence of steric constraints, and thus justify the simple additive form of the standard nearest neighbour energy model. Unfortunately, this is not always correct: some large secondary structures describe configurations with impossible molecular densities, which are less straightforward to identify and counteract. For example, consider a secondary structure *M*_*n*_ that folds into a depth-*n* binary tree, e.g. $M_{3}=a(a(a(h)a(h))a(a(h)a(h)))$. Domain a appears 2^*n*^ − 1 times, implying that the mass of the molecule scales as *O*(2^*n*^). However, every domain is within distance *O*(*n*) of the root because it is a tree of depth *n*, implying that the volume of the molecule fits within a sphere of radius *O*(*n*) and thus volume *O*(*n*^3^). Regardless of constant factors, a contradiction arises for large *n*, at which point steric effects would prevent fitting the full molecular mass within the volume implied by the tree-like secondary structure. In such cases, the standard nearest neighbour energy model will be woefully inaccurate.

2 A unimolecular pattern-matching rule can be written in *n* *equivalent circular permutations*, where *n* is the number of ‘?’ wildcards in each pattern. In order to rotate a pattern, the structure enclosing the pattern becomes a new wildcard, and shifts one of the previous wildcards out of the pattern. For example, see figure 2*d*: the product pattern of three-way-fw is equivalent to the reactant of three-way-bw. The third circular permutation is not shown, but it would start at the unbound domain r. Our implementation derives a unique permutation (canonical form) for each complex, which saves computation time whenever the products of a pattern matching rule are compared to the set of known configurations, but it requires us to apply all *k* permutations of pattern matching rules to the canonical form of a complex.

3 Consider a pathological reaction network such as *a* → *b* + *c*, *c* → *a*. These types of reactions prevent us from finding meaningful SCCs. Such a reaction network would not be generated by our enumerator, because Peppercorn requires that the number of DNA strands are conserved across reactions; this network would also *not* satisfy property 3.

## Data accessibility

Raw data for case studies are available online at https://github.com/DNA-and-Natural-Algorithms-Group/peppercornenumerator.

## Appendix A. Reaction enumeration semantics

Different reaction enumeration semantics enable us to vary the size of the reaction network in a controlled manner. That means in the simplest form, we can exclude a reaction type, e.g. four-way branch migration reactions, or we can vary the dissociation threshold *L*. Here, we will discuss two alternative semantics: **reject-remote** reduces the state space significantly but might thereby exclude states and reactions of biophysical importance, while **max-helix** reduces the state-space whenever there exists a more compact domain-level representation for a sequence of same reaction types. Examples of how those semantics effect reaction enumeration are shown in figure 12.

*Max-helix notion.* An enumeration as described previously is inherently dependent on the domain-level representation of the system. For example, a long domain cannot unbind, but if it is represented as the concatenation of two or more short domains, then those can dissociate by a sequence of two or more fast reactions. The max-helix reaction semantics reduces the effects of the chosen domain-level representation. Let $\underset{↔}{r}$ denote a maximal-length sequence of immediately adjacent domains that can engage in the same reaction type, then we can formulate max-helix semantics as a simple extension of the pattern-matching rules:

— bind11: $\;\underset{↔}{r}$ ? $\underset{↔}{r*}\;$ -> r( ? )
— bind21: [? $\underset{↔}{r}$ ?] + [? $\underset{↔}{r*}$ ?] -> [? r( ? + ? ) ?]
— open: $\underset{↔}{r(}\;$ ? $\underset{↔}{)}$ -> r ? r*
— three-way-fw: $\underset{\leftarrow }{r(}\;$ ? $\underset{\leftarrow }{r}\;$ ? $\underset{\to }{)}$ -> r ? ( ? )
— three-way-bw: $\underset{\to }{r}\;$ ? $\underset{\to }{r(}\;$ ? $\underset{\leftarrow }{)}$ -> r( ? r ? )
— four-way: $\underset{\to }{r(}\;$ ? $\underset{\leftarrow }{)}\;$ ? $\underset{\to }{r(}$ ? $\underset{\leftarrow }{)}$ -> r( ? r*( ? ) ? )

Max-helix requires all involved components of reactants to have the correct domain-level sequence such that the rate can be calculated in terms of a single overall reaction. Note that bind and open reactions are trivial cases where any matching domain can extend to the left and to the right. It is important to include both directions, such that any domain corresponding to a partial open or bind reaction will extend to the same max-helix pattern match. Initial matches for three-way and four-way branch migration can only be extended in one direction under max-helix notion. For example, the three-way branch migration reaction from A to A3 in figure 12*a*, can only be initiated from domain c and extended until domain b. The direction of potential extensions can also be seen directly from the kernel notation: any ‘?’ must be well formed, so there must never be arrows that extend a given helix into two independent ‘?’ regions.

*Reject remote branch-migration.* DSD systems are often designed using simplified variants of the reaction rules described above. In particular, branch migration domains are often immediately adjacent to already bound domains. One might therefore choose to enumerate a system using these constrained reaction types. The corresponding modifications of our pattern matching are shown below; we leave it as an exercise for the reader to draw the corresponding secondary structures:

— three-way-fw-rr: r( ? r a( ? ) ) -> r ? r( a( ? ) )
— three-way-bw-rr: r( a( ? ) r* ? ) -> r( a( ? ) ) ? r*
— four-way-rr: a( r( ? ) b( ? ) r( ? ) ) -> a( r( ? r*( b( ? ) ) ? ) )

The reject-remote notion has a potentially undesirable feature that unimolecular reactions that do not involve dissociation are no longer guaranteed to be reversible (figure 12*b*). This is easily visible for three-way branch migration reactions because of their asymmetry. Note that if we remove the bound domain a( ) from the expressions, then the reactant of the backward three-way reaction is a circular permutation of the product of the forward three-way reaction

r  ? r (  ? ) =r ∗ (  ? r  ? ) =r (  ? r ∗  ? )  

but since we have the constraint a(. . .) this is not guaranteed. The reject-remote conditions for four-way branch migration enforces that the initial configuration is a proper four-way junction (where a(. . .) and b(. . .) form two opposite arms). Consequently, if the proximal toehold four-way branch migration reaction does not yield a properly formed four-way junction, then the reaction will be irreversible.

## Appendix B. Justification of the condensed reaction algorithm

We will now justify the algorithm for condensing reactions with several theorems that show the relationship between the condensed reaction network $\hat{G}=(\hat{C},\hat{R})$ and the detailed reaction network *G* = (*C*, *R*). Here, we treat the rate-independent model, for which bimolecular detailed reactions are always classified as slow and unimolecular detailed reactions are always classified as fast. Recall from §3 that S(x) is the strongly connected component of a complex *x*, F(x) denotes the set of fates of a complex *x*, and R(r) is the set of fates of a reaction *r*. We introduce two further definitions.

First, we need a notion of what kind of processes from the detailed reaction network are actually included in the condensed reaction network. We define a **fast transition** *T*_⦃*x*⦄→*B*_ to be a sequence of (zero or more) unimolecular reactions that begin from a single initial (transient or resting) complex *x* and result in a multiset *B* of resting complexes. A **resting transition** *T*_⦃*a_1_*__*,a_2_*__⦄→*B*_ is a sequence of detailed reactions starting with a bimolecular (slow) reaction (by definition between two resting complexes *a*_1_ and *a*_2_), followed by a sequence of (zero or more) unimolecular (fast) reactions that can occur if the system starts with just *a*_1_ and *a*_2_ present, and such that the final state *B* consists exclusively of resting complexes.

Second, we need a notion of correspondence between some reaction in the condensed reaction network and a *transition* that can occur in the detailed reaction network. For a given multiset of resting macrostates $\;\hat{A}=$ ⦃ ${\;\hat{A}}_{1},{\;\hat{A}}_{2},\ldots$ ⦄, where each ${\hat{A}}_{i}=\{a_{i,1},a_{i,2},\ldots \}$, a **representation** of $\;\hat{A}$ is a set containing a choice of *one* complex *a*_*i*,*j*_ from each ${\;\hat{A}}_{i}$. Note that if any of the sets ${\hat{A}}_{i}\in \;\hat{A}$ are not singletons, then there are multiple representations of $\;\hat{A}$. For example, if $\;\hat{A}=$ ⦃ ${\;\hat{A}}_{1},{\;\hat{A}}_{2}$ ⦄, ${\;\hat{A}}_{1}=\{a_{1,1},a_{1,2}\}$, and ${\;\hat{A}}_{2}=\{a_{2,1},a_{2,2}\}$, then there are four possible representations of $\;\hat{A}$: {*a*_1,1_, *a*_2,1_}, {*a*_1,2_, *a*_2,1_}, {*a*_1,1_, *a*_2,2_}, or {*a*_1,2_, *a*_2,2_}. We can write $A∼\;\hat{A}$ to indicate^4^ that *A* is a representation of $\;\hat{A}$.

Lemma B.1. *For every complex* *x*, *and for every fate* *F* *in the set of fates* F(x), *and for every* *B* *such that* *B* ∼ *F*, *there exists a fast transition* *T*_⦃*x*⦄→*B*_.

Proof. Consider a single fate F∈F(x). In the base case where *x* is a resting complex, then F(x)={S(x)} is singleton, and we take F=S(x). If S(x) is non-singleton, then any transition *T*_⦃*x*⦄→⦃*b*⦄_ between *x* and another complex b∈S(x) will satisfy the property that *B* ∼ *F* when *B* = ⦃*b*⦄. If S(x) is singleton (S(x)={x}), then the transition *T*_⦃*x*⦄→⦃*x*⦄_ is degenerate, but still satisfies the propery that *B* = ⦃*x*⦄ and ⦃*x*⦄ ∼ *F*. When *x* is not a resting complex, recognize that each fate F∈F(x) was generated by application of the recursive case of equation (3.4), in which a union is taken over outgoing reactions from S(x). That is, each fate F∈F(x) is generated by some reaction *r* = (*α*, *β*) that is outgoing from S(x). Specifically, *F* is one element of the set $R(r)=F(\beta )={⨁}_{b\in \beta }F(b)$. For any *B* ∼ *F*, the fast transition *T*_⦃*x*⦄→*B*_ can thus be accomplished by first following *r*, followed by the concatenation of *T*_⦃*b*⦄→F(*b*)_ for each *b* ∈ *β*. By induction, we recognize that, for any complex *x* and fate F∈F(x), a fast transition can be accomplished from *x* to any *B* ∼ *F*. ▪

Theorem B.2 (Condensed reactions map to detailed reactions). *For every condensed reaction* $\hat{r}=(\;\hat{A},\hat{B})$, *for every* *A* *that represents* $\;\hat{A}$, *and for every* *B* that represents $\hat{B}$, *there exists a detailed resting transition* *T*_*A*→*B*_.

Proof. First, recognize that every condensed reaction $\hat{r}=(\;\hat{A},\hat{B})$ was generated by some bimolecular reaction *r* = (*A*, *A*′), where *A* contains only resting complexes and represents $\;\hat{A}$. Therefore, we must only show that there exists a fast transition *T*_*A*′→*B*_, such that $B∼\hat{B}$. We recognize that the multiset of products $\hat{B}$, of the condensed reaction $\hat{r}$, was generated from one element of $R(r)=F(A^{\prime })$. Therefore, $\hat{B}$ is an element of $F(A^{\prime })$. By lemma B.1, there exists a detailed transition *T*_*A*′→*B*_, such that $B∼\hat{B}$. Therefore, there exists a transition *T*_*A*→*B*_ such that $A∼\;\hat{A}$ and $B∼\hat{B}$. ▪

Lemma B.3. *For every complex* *x*, *each fast transition* *T*_⦃*x*⦄→*B*_, *such that* *B* *contains only resting complexes, corresponds to exactly one fate* F∈F(x). *Specifically, there exists some fate* F∈F(x) *such that* *B* ∼ *F*.

Proof. Consider the base case where *x* is a resting complex; in this case, all fast transitions from *x* must lead to another resting complex in S(x). F(x)={S(x)}, by equation (3.4), and therefore this transition corresponds to the fate F=S(x). Consider some detailed fast transition *T*_⦃*x*⦄→*B*_ such that *B* = ⦃$\;b_{1},b_{2},\ldots \;$⦄ contains only resting complexes. We recognize that, if *x* is *not* a resting complex, there must be at least one reaction in this process. The transition begins with this initial reaction *r*^0^ = (⦃ x ⦄, *Y*); *Y* may have multiple products, each of which decays independently to some complex or set of complexes in *B*. For some reaction *r*_*i*_ = (*A*_*i*−1_, *A*_*i*_), by applying equation (3.4), we recognize that if a fate *F* is reachable from *A*_*i*_, then it is reachable from *A*_*i*−1_. That is, for some fate *F*, $F\in F(A_{i})\Rightarrow F\in F(A_{i-1})$. This means that, for some prior reaction *r*_*i*−1_ = (*A*_*i*−2_, *A*′_*i*_) such that *A*_*i*_ ⊆ *A*′_*i*_ (that is, a reaction *r*_*i*−1_ that produces the reactant of *r_i_*) $F\in R(r_{i})\Rightarrow F\in R(r_{i-1})$. Next, we note that the set of products *B* of the transition *T*_⦃*x*⦄→*B*_ must represent some fate; that is, *B* ∼ *F*. Since *B* consists exclusively of resting complexes, *F* = ⦃S(b) : b∈B ⦄. Multiple reactions *r*_1_, *r*_2_, …*r*_*m*_ may have produced complexes that are in *B*; let us denote the set of reactions whose products are in *B* by *R*_*B*_: $R_{B}=\{r_{i}=(A_{i},B_{i})\in \;$*T*_⦃*x*⦄→*B*_$:\;B_{i}\subseteq B\}$; *B* is therefore the sum of the products of these reactions: $B=\sum_{r_{i}=(A_{i},B_{i})\in R_{B}}B_{i}$. Because *B* ∼ *F* and equation (3.4) includes all possible sums of R(B), this means that if we choose fates $F_{i}\in F(r_{i})$ for each of those reactions, there exists some set {*F*_1_, *F*_2_, …, *F*_*m*_} such that *F*_1_ + *F*_2_ + · · · + *F*_*m*_ = *F*. Consider one of the reactions *r*_*i*_ = (*A*_*i*_, *B*_*i*_) ∈ *R*_*B*_, that produces complex(es) in *B*. Each fate $F^{\prime }\in R(r_{i})$ is *also* a fate of any reaction that produces *A*_*i*_. This means that, for *r*_*i*_, the particular fate *F*_*i*_ ∈ {*F*_1_, *F*_2_, …, *F*_*m*_} satisfying $\sum_{\;j=1}^{m}F_{j}$ must *also* be a fate of any reaction that produces *A*_*i*_. By induction, we can work backwards from *r*_*i*_ all the way to the initial reaction *r*^0^, and recognize that *F*_*i*_ ⊆ *F*^0^ for some $F^{0}\in R(r^{0})$. The same is true for all reactions *r*_*i*_ ∈ *R*_*B*_. Because the recursive case of equation (3.4) sums over all combinations of fates for all such pathways, the *F*_1_ + *F*_2_ + · · · + *F*_*m*_ = *F* must be a member of $R(r^{0})$, and therefore a member of F(x). ▪

Theorem B.4 (Detailed reactions map to condensed reactions). *For every detailed resting transition* *T*_*A*→*B*_, *there exists a condensed reaction* $\hat{r}=(\;\hat{A},\hat{B})$ *such that* *A* *represents* $\;\hat{A}$ *and* *B* *represents* $\hat{B}$.

Proof. Since *T*_*A*→*B*_ is a transition between two sets (*A* and *B*) of detailed resting complexes, the transition consists of two steps: first, a bimolecular reaction *r* = (*A*, *A*′) converts *A* to *A*′; second, a series of unimolecular reactions convert the complexes in *A*′ to *B*. The algorithm generates one or more condensed reactions for each detailed bimolecular reaction. Specifically, the algorithm generates one condensed reaction for each combination of fates of the products in *A*′. That is, each of the condensed reactions is generated from one element in $R(r)={⨁}_{a^{\prime }\in A^{\prime }}F(A^{\prime })$. By lemma B.3, for each product *a*′ ∈ *A*′, $F(a^{\prime })$ corresponds to the set of possible transitions from *a*′ that result in some resting macrostate. Therefore, we can choose any possible fast transition between *T*_*A*′→*B*_, and it will correspond to some element of R(r)—and therefore to a condensed reaction $\hat{r}=(\;\hat{A},\hat{B})$. ▪

Intuitively, these two theorems mean that the condensed reaction network effectively models the detailed reaction network, at least in terms of transitions between resting macrostates. The first theorem shows that a condensed reaction must be mapped to a suitable sequence of reactions in the detailed reaction network. The second theorem shows the converse—that any process in the detailed reaction network is represented by the condensed reactions. Having proved these theorems, we propose the following corollaries that extend this reasoning from individual (detailed and condensed) reactions to sequences of condensed and detailed reactions. We omit the proofs.

Corollary B.5. *For any sequence of condensed reactions starting in some initial state* $\;\hat{A}$ *and ending in some final state* $\hat{B}$, *and for any* $A∼\;\hat{A}$ *and for any* $B∼\hat{B}$, *there exists a sequence of detailed reactions starting in* *A* *and ending in* *B*.

Corollary B.6. *Conversely, for any sequence of detailed reactions starting in some multiset of resting complexes* *A* *and ending in some multiset of resting complexes* *B*, *there exists a sequence of condensed reactions starting in* $\;\hat{A}$ *and ending in* $\hat{B}$ *such that* $A∼\;\hat{A}$ *and* $B∼\hat{B}$.

## Appendix C. Biophysical kinetics model

The model presented below in §C.1 calculates approximate reaction rates for different types of detailed reactions from the length of involved domains. In §C.2, we present how to calculate reaction rate constants for condensed reactions from the detailed reactions along with our algorithm for reaction condensation. Both sections rely on the standard mass-action model for chemical kinetics. For simulations with deterministic continuous semantics, i.e. bulk systems, we use ODEs to describe the dynamics. Let ρ(r) be the molar rate of some reaction *r* = (*A*, *B*), with reactants *A* = *a*_1_, *a*_2_, …, then

C 1 $$\rho (r)=k\prod_{a\in A}{\left[a\right]}^{\chi {\;}_{-}^{r,a}}$$

and

C 2 $$\frac{d[a]}{dt}=\sum_{r}(\chi {\;}_{+}^{r,a}-\chi {\;}_{-}^{r,a})\rho (r),$$

where [*a*] represents the concentration of some species *a*, *k* is the rate constant for reaction *r*, and $\chi {\;}_{+}^{r,a}$ and $\chi {\;}_{-}^{r,a}$ are the stochiometry coefficients of *a* as a product or reactant in *r*, respectively. Since the reaction enumerator has no knowledge of concentrations, the problem of estimating the rate ρ(r) of a reaction boils down to estimating the rate constant *k*. Implicit in this choice of rate law is the assumption that all reactions are elementary (meaning there is only a single transition state between the reactants and the products such that once the reaction occurs, the products are released effectively instantaneously). For simulations with discrete stochastic semantics, i.e. small-volume systems, the same CRN is described as a continuous-time Markov chain (CTMC) with rate parameters derived from the bulk rate constants in a standard way that depends on the reaction volume *V*. In the CTMC model, the probability that the next event is reaction *r* is linearly proportional to its propensity, i.e. the instantaneous reaction rate. Thus the assumption that reactions are instantaneous amounts to assuming that the probability a particular reaction occurs next is directly related to the expected time that the reaction will take to complete.

Since we use CRNs with mass-action kinetics for both the detailed and condensed network representations, it is reasonable to ask how well the assumptions hold. While base-pairing changes in models of sequence-level secondary structure kinetics [40,79] may usually represent physically elementary reactions (with some exceptions where non-Markovian effects have been observed [80]), for our domain-level representation, this may not be the case, as many DSD reactions have a complex transition state landscape and involve many intermediate states. As an example, the probability that a length-*n* three-way branch migration process completes rather than returns to the start (and thus the probability that this domain-level reaction occurs rather than a competing branch migration reaction) scales as 1/*n*, while the actual expected time to complete scales as 1/*n*^2^ due to the random walk [38]—violating the strict linkage between probability and rate that is inherent in CTMCs. When considering condensed reactions, this issue is compounded, as there may be complex trajectories through transient states before a resting macrostate is reached. When assigning a rate model for detailed reactions at the domain-level, we acknowledge and accept this limitation; when forced to choose, we prioritize accuracy for probabilities rather than reaction times because in the limit of low concentration, the duration for a reaction becomes a negligible, whereas the probability that a trajectory through transients arrives at a given fate remains pertinent.

**C.1. Approximate detailed reaction kinetics**

It is important to emphasize that our formulae for reaction rate constants, although based on experimental evidence and intuition, are heuristic and approximate; they serve as a placeholder until a more accurate and more general model can be developed. The kinetics of a real physical system will be affected by parameters outside the consideration of our model. For example, the nucleotide sequences of each domain, the temperature and salt concentrations all affect the binding energies and hybridization kinetics. The formulae here assume ‘well-designed’ sequences with perfect Watson–Crick base pairing (e.g. *x* is complementary to *x**), perfect binding orthogonality (e.g. *x* does not bind to either *y* or *y**), and experimental conditions similar to 25°C and 10 mM Mg^2+^ and pH 8.0. As mentioned above, they focus on kinetics and trajectory probabilities; as a consequence of these choices, they are not guaranteed to satisfy detailed balance and we cannot claim a well-defined energy for complexes.

Ultimately, our rate model must provide bimolecular rate constants for bind21 reactions and unimolecular rate constants for bind11, open, three-way-fw, three-way-bw and four-way reactions. The rate constants will depend not only on the lengths of the relevant domains but also upon features of the local secondary structure. The bimolecular bind21 reaction has the fewest such context-dependent considerations, so we will start there.

*Bimolecular binding rate constant (bind21)*. A bimolecular bind reaction, i.e. with arity (2,1), is dominated by the rate limiting step of forming the initial stable contact. The number of opportunities to initiate successfully scales with the length ℓ, so the binding rate constant is approximated as *k*_bind21_ = 3ℓ × 10^5^ M^−1^s^−1^, following the empirical formulae of Wetmur [26] for complementary strands shorter than roughly 100 nucleotides. The assumption is that the initiation of a bimolecular binding reaction is dominated by the case where binding is well-aligned, even though there are up to ℓ^2^ possibilities of forming an initial inter-molecular base-pair and potentially more initial contacts with other unpaired domains.

*Opening reaction rate constant (open)*. In our model, open reactions are predominantly important for toehold dissociation. Using the previously introduced parameters for bimolecular binding, we can calculate *k*_open_ as the reverse reaction of *k*_bind21_ using the parameters of the nearest neighbour energy model. The equilibrium constant of the reaction relates to the change in free energy Δ*G*° as:

C 3 $$K_{\mathrm{eq}}=\frac{k_{\mathrm{open}}}{k_{\mathrm{bind}21}}=e^{\frac{\Delta G^{\circ }}{RT}}\;\text{M},$$

where for the gas constant, we use *R* = 1.987 cal mol^−1^ K^−1^. According to SantaLucia & Hicks [62], the average energy of a single base stack is Δ*G*_stack_ = −1.7 kcal mol^−1^ at *T* = 298 K (25°C) in a 1 M sodium buffer (which is thermodynamically similar to a 10 mM magnesium buffer), and the penalty for strand association is Δ*G*_assoc_ = 1.9 kcal mol^−1^. The typical free energy change of a hybridization reaction according to the nearest neighbour energy model is therefore approximately

C 4 $$\Delta G^{\circ }=\ell \cdot \Delta G_{\mathrm{stack}}+\Delta G_{\mathrm{assoc}},$$

where the length ℓ roughly corresponds to the number of stacking interactions and we ignore possible dangle and coaxial stacking contributions. We therefore, compute the reaction rate constant for dissociation as

C 5 $$k_{\mathrm{open}}=k_{\mathrm{bind}21}{\text{e}}^{(\ell \times \Delta G_{\mathrm{stack}}+\Delta G_{\mathrm{assoc}})/RT}\;\text{M}=3\ell \times 10^{5}\times {\text{e}}^{(-1.7\cdot \ell +1.9)/298.15\cdot R}\;{\text{s}}^{-1}=7.41\ell \times 10^{6}\times {\text{e}}^{-2.86\ell }\;{\text{s}}^{-1}.$$

*Unimolecular binding rate constant* (*bind11*). The unimolecular bind reaction depends on the linkers connecting the binding domains, that is, it depends on the secondary structure immediately on either side of the domains that bind. The intuition is that the linkers on either side determine the effective local concentration for formation of the initial base pair in the binding domain, or other potential geometric constraints that prove rate-limiting. We distinguish three cases, providing distinct formulae for *k*_bind11_ for each:

First, **zipping** is the unconstrained elongation of an already formed helix. This case occurs when on exactly one side of the binding domains is an already-bound domain, while on the other side is an open loop or a closed loop (with the exception of the bubble closing case described below). The per-base-pair rate of zippering (with an open loop on one side) has been estimated between 10^6^ s^−1^ and 10^10^ s^−1^ for DNA [26,63,64]. For numeric stability, we use *k*_zip_ = 10^6^ s^−1^, which is still orders of magnitude faster than the rate-limiting steps of most reaction pathways. Treating zippering of the entire domain as an elementary step, we arrive at *k*_bind11_ = *k*_zip_/ℓ = 10^6^/ℓ s^−1^ for this case.

Second, **bubble closing** corresponds to simultaneous zipping from both ends of a domain. Thus, this case occurs when both sides of the binding domains are immediately flanked by an already-bound domain. Perhaps surprisingly, this reaction has been found to be dominated by the rate limiting step for closing the *last* base-pair [32], and we use *k*_bind11_ = 10^4^ s^−1^.

The third case occurs when both sides of the binding domains are flanked by an open loop or a closed loop that matches neither the zippering or bubble closing cases described above. In general, we call this case of unimolecular binding to be **loop closing**, but the simplest case—where one side is an open loop and the other side is just a single-stranded domain—is the familiar and well-studied **hairpin closing** reaction. As other cases are less well studied, we derive our general rate formula as a generalization of hairpin closing. Specifically, we use *k*_bind11_ = *C*(ℓ) × *k*_zip_, where *C*(ℓ) represents a ‘closing fraction’ that accounts for the formation of the first base pair being the rate-limiting step, but depending on the relevant loop length ℓ, the relevant nucleotides will be appropriately positioned to zip up only a fraction of the time. Below, we show how ℓ and *C*(ℓ) are calculated for genuine hairpins and for more complex generalizations.

Experimental studies of DNA hairpin opening and closing kinetics [31,33,36,39,65], usually with poly-T or poly-A loops and in sodium and magnesium buffers of lower ionic strength than our default here, disagree by sometimes more than a factor of 10—but all are consistent with a power-law scaling for the dependence of the closing rate on the loop length. Here, we attempt to strike a balance, using a simplified expression for the rate of hairpin closing with a cut-off for short hairpins:

C 6 khp=min{ 106×ℓ−2.5 s−1 33 000 s−1,

where ℓ is the number phosphate links in the hairpin loop (i.e. the number of unpaired nucleotides plus one). For loop-closing reactions that are open on one side and have a single-stranded domain (or domains) on the other side, we simply use

C 7 $$k_{\mathrm{bind}11}=C(\ell )k_{\mathrm{zip}}=k_{\mathrm{hp}}.$$

Since *k*_zip_ is known, this implicitly defines *C*(ℓ).

Due to a lack of systematic biophysical studies, further generalization is based on unverified intuition. We first generalize to loop-closing reactions that are open one side, while the nascent loop on the other side consists both of single-stranded domains and helix stems (that may lead to other secondary structure). A mixed-composition loop with *h* helix ends and *u* nucleotides of single-stranded domains will have an effective length ℓ=1+u+h+(2.0/0.43) h in nucleotides, because each helix stem contributes one phosphate link of length 0.43 nm and has a diameter of approximately 2.0 nm. For example, if we consider the opening and closing of the central multiloop of figure 1 via domains d and d*, *u* is the combined length of domains e, i, n and r, while *h* = 3.

Finally, in the case where the complementary domains form closed loops on both sides, we calculate ℓ for both and use just the minimal value, as the smaller loop is expected to provide the stronger constraints and determine the effective local concentration. An example of this would be the opening and closing of the helix formed by domains j and j* in figure 1.

*Branch migration rate constants* *(three-way-fw, three-way-bw and four-way)*. We distinguish two properties of branch migration: the average number of attempts until a branch migration reaction is successful depends on the length of the domain ℓ, while the expected time to complete branch migration corresponds to a random walk scaling with ℓ^2^ [34,38]. Assume we have the rate for initiating the first step of branch migration *k*_bminit_ and the rate for subsequent branch migration steps *k*_bmstep_, then the rate for initiating a successful branch migration is *k*_bm_ = *k*_bminit_/ℓ. Note that this rate is independent of *k*_bmstep_, as we ignore the time spent in the branch migration process.

We distinguish several cases based on how the branch migrating domains are connected to each other. In three-way branch migration, we have the canonical (direct) case, exemplified by reaction A43→A4 in figure 12*a*; the canonical (two-tailed) case, exemplified by reaction A34→A43 in figure 12*a*; and the non-canonical (remote) case, exemplified by reaction A3→A33 in figure 12*a*. In four-way branch migration, we have the canonical (closed) case, exemplified by reaction A→A4 in figure 12*a*; the canonical (open) case, exemplified by reaction A3→A34 in figure 12*a*; and the non-canonical case, exemplified by what could happen in the multiloop of figure 1*a* if domain d were identical to domain j*.

For canonical (direct) three-way branch migration, where the first displacing nucleotide extends from a helix stem that is coaxially stacked with the helix being displaced, and where the first nucleotide of the displaced domain is the end of the strand, we use *k*_bminit_ = 0.333 × 10^3^ s^−1^ as inferred experimentally [38], so *k*_three−way−fw_ = *k*_three−way−bw_ = 0.333 × 10^3^/ℓ s^−1^. For the canonical (two-tailed) case, the first displacing nucleotide again extends from a helix stem that is coaxially stacked with the helix being displaced, but now the first-displaced nucleotide of the displaced domain has some extension, which must not be connected to the displacing domain (except via the displaced domain). In this case, there are overhanging nucleotides on both sides of the initiation side, as in an intermediate step during branch migration, and therefore we use *k*_bminit_ = *k*_bmstep_ = 10^4^ s^−1^ as inferred experimentally [38], so *k*_three−way−fw_ = *k*_three−way−bw_ = 10^4^/ℓ s^−1^.

For canonical four-way branch migration, initiated from a perfect Holliday junction consisting of four helix stems, we use *k*_bminit_ = *k*_bmstep_ = 0.333 s^−1^, which is inferred from Panyutin & Hsieh’s measurements [28] in 10 mM Mg^2+^ at 50°C and 37°C, and extrapolating to 25°C. Thus, for this ‘closed loop’ case we have *k*_four−way_ = 0.333/ℓ s^−1^.

Four-way branch migration initiated from a Holliday junction with an open loop (i.e. two opposing helix stems with identical sequence, connected by a third stem on one side but with an open loop on the other side) has been studied by Dabby [37], who reports *k*_bminit_ = 0.0093 s^−1^. Thus, for this ‘open loop’ case we have *k*_four−way_ = 0.0093/ℓ s^−1^.

Other cases of three-way and four-way branch migration have been less well studied, to our knowledge. For simplicity, we treat all cases with a consistent approach: using the *k*_bminit_ from the closest category above (three-way, closed four-way, or open four-way), the rate for initiating branch migration is slowed down based on the estimated fraction of time that first displacing nucleotide is in position to initiate branch migration. We use *C*(ℓ) for this fraction, following the method discussed above for unimolecular binding reactions.

For non-canonical (remote) three-way branch migration, there may be a linker either to the left or to the right of the first displacing nucleotide, or both—in which case the shorter linker is used, as before. The displacing single-stranded domain(s) will be part of one of the linkers, if the side containing it is connected, because that linker’s length is relevant for the local concentration of the first displacing nucleotide. Thus, for such cases, *k*_three−way−fw_ = *k*_three−way−bw_ = *C*(ℓ) × *k*_bminit_/ℓ.

For non-canonical (remote) four-way branch migration, using the value of *k*_bminit_ appropriate for the closed (two linkers) or open (one linker) case, we similarly use the smallest available value of ℓ and compute *k*_four−way_ = *C*(ℓ) × *k*_bminit_/ℓ.

**C.2. Derivation of condensed reaction kinetics**

The overall rate for a condensed reaction is proportional to the rates of the detailed reactions, weighted by the joint probability that the reactant complexes are actually present, and that the product complexes decay to the correct resting macrostates with the correct probabilities. That is, the overall rates of interactions between molecules should be consistent between the detailed model and the condensed model, and the probabilities of particular outcomes from an interaction also should be consistent. However, because the condensed model is represented as instantaneous reactions with no intermediate steps, the time it takes from the initial interaction to the eventual release of products (which is included in the detailed model) is not represented in the condensed model.

As usual, let *G* = (*C*, *R*) be a detailed reaction network and $\hat{G}=(\hat{C},\hat{R})$ the corresponding condensed representation (see §3). A condensed reaction is $\hat{r}=(\;\hat{A},\hat{B})$ where $\;\hat{A}$ and $\hat{B}$ are multisets of resting macrostates from $\hat{C}$. Let $R_{\;\hat{A}}$ be the set of all detailed *slow* reactions with reactants in resting macrostate(s) $\;\hat{A}$. For example, if $\;\hat{A}$ is a multiset of two resting macrostates $\;\hat{A}=\left\{\right|\;{\;\hat{A}}_{1},{\;\hat{A}}_{2}\;\left|\right\}$, then $R_{\;\hat{A}}$ is given by all detailed bimolecular reactions that satisfy

$$R_{\;\hat{A}}=\{r=(\{|\;a_{1},a_{2}\;|\},B)\;:\;r\in R,a_{1}\in {\;\hat{A}}_{1},a_{2}\in {\;\hat{A}}_{2}\},$$

with arbitrary products *B*. In order to predict the rate constant $k_{\hat{r}}$ of a condensed reaction, recall that we assume the system to be in steady state relative to the fast reactions. That means the rate constant for a condensed reaction depends on the **steady-state probability** of a reactant complex in its resting macrostate, $P[a_{i}\;:\;{\;\hat{A}}_{i}]$, and the **decay probability** that product complexes *B* react to complexes that represent resting macrostates $\hat{B}$, $P[T_{B\to \hat{B}}]$. The approximate condensed rate constant $k_{\hat{r}}$ for our bimolecular example can, therefore, be calculated as

$$k_{\hat{r}}=\sum_{r=(\left\{\;\right|a_{1},a_{2}\left|\;\right\},B)\in R_{\;\hat{A}}}P\;\left[a_{1}\;:\;{\;\hat{A}}_{1}\right]\times P\left[a_{2}\;:\;{\;\hat{A}}_{2}\right]\times k_{r}\times P\left[T_{B\to \hat{B}}\right],$$

where *k*_*r*_ represents the rate constant for the *detailed* reaction *r*. The sum is over all detailed reactions which consume one complex from each resting set in $\;\hat{A}$. Hence, if *r* produces products *B* which can never be converted to the resting macrostates in $\hat{B}$, then this term will be 0. This example is illustrated in figure 13.

The general form including the unimolecular and bimolecular case is given by

C 8 $$k_{\hat{r}}=\sum_{r=(A,B)\in R_{\;\hat{A}}}\left(k_{r}\times P\left[T_{B\to \hat{B}}\right]\times \prod_{a_{i}\in A}P\left[a_{i}\;:\;{\;\hat{A}}_{i}\right]\right).$$

To calculate $P\left[a_{i}\;:\;{\;\hat{A}}_{i}\right]$, and $P\left[T_{B\to \hat{B}}\right]$, it is helpful to think of each transient or resting macrostate as an individual, irreducible CTMC. The remainder of this section derives $P\left[a_{i}\;:\;{\;\hat{A}}_{i}\right]$ for resting macrostates, and $P\left[T_{B\to \hat{B}}\right]$ for transient macrostates.

*Resting macrostates.* We can treat a single resting macrostate *A* = {*a*_1_, *a*_2_, …, *a*_*L*_} to be a continuous-time Markov process, continually transitioning between each of the *L* states. The dynamics of this process can be written as a matrix $T\in R^{L\times L}$, where the elements *T*_*ij*_ are the rates (possibly zero) of the reaction from state *j* to state *i*, which we denote ρ(j→i), and each diagonal element is the negative sum of the column

C 9 $$T_{ij}=\left\{ \begin{array}{cc} \rho (j\to i)\; & \mathrm{if}\;i\neq j\;\\ -\sum_{\;j=1,j\neq i}^{L}T_{\;ji}\; & \mathrm{if}\;i=j.\;\\ \; \end{array}\right.$$

Let **s**(*t*) = (*s*_1_, *s*_2_, …)^*T*^ be an *L*-dimensional vector giving the probabilities, at time *t*, of being in any of the *L* states. The continuous-time dynamics of this process obeys

$$\frac{ds}{dt}=Ts(t).$$

For a resting macrostate, we assume that the system has reached steady state, and so **s** is not changing with time. We therefore find the **stationary distribution** $\hat{s}$ of this process by setting *d***s**/*dt* = 0, and recognizing that $\hat{s}$ is the right-eigenvector of **T** with eigenvalue zero. Given the stationary distribution $\hat{s}={\left({\hat{s}}_{1},{\hat{s}}_{2},\ldots ,{\hat{s}}_{L}\right)}^{T}$, we recognize that

C 10 $$P\left[a_{i}\;:\;{\;\hat{A}}_{i}\right]={\hat{s}}_{i}.$$

*Transient macrostates*. To calculate the decay probability $P\left[T_{B\to \hat{B}}\right]$ that complexes in *B* react to complexes that represent $\hat{B}$, we cannot use the stationary distribution since there are outgoing fast reactions that exit this macrostate. However, we can include the *e* outgoing reactions, using an (*L* + *e*)-state Markov process, where each of the *e* states is absorbing. This enables us to calculate the probability that, having entered the macrostate in some state *i* ∈ {1, …, *L*}, it will leave via some reaction *j* ∈ {*L* + 1, …, *L* + *e*}. Hence, outgoing reactions and complexes are discussed consistently as states in the same Markov process. We first derive how to calculate the **decay probability** of a single complex P[x→F] and then express the decay probability of multiple species to a given fate *F* as a combination of all pathways whose fates sum up to *F*. Note that here, we are using a discrete-time Markov process because we are not concerned with how long it takes for *B* to reach $\hat{B}$, but just the probability that $\hat{B}$ is reached.

Assume the macrostate is again given by *A* = {*a*_1_, *a*_2_, …, *a*_*L*_}. Let $Q\in R^{L\times L}$ be the matrix of transition probabilities *within* the macrostate, such that *Q*_*ij*_ is the probability that, at a given time the system’s next transition is from state *i* to state *j*, where *i*, *j* ∈ {1, …, *L*}:

C 11 $$Q_{ij}=\frac{k_{ij}}{\sum_{\;j^{\prime }=1}^{L+e}k_{ij^{\prime }}}.$$

Now let us use the same principle to define a matrix $R\in R^{L\times e}$, where *R*_*ij*_ represents the probability that the system in state *i* ∈ {1, …, *L*} transitions directly to absorbing state *j* ∈ {*L* + 1, …, *L* + *e*}. Based on transition probabilities *Q*_*ij*_ we calculate the **fundamental matrix** **N**, which contains the expected number of visits to state *j*, starting from state *i* as

C 12 $$N=\sum_{k=0}^{\infty }Q^{k}={\left(I_{L}-Q\right)}^{-1},$$

where **I**_*L*_ is the *L* × *L* identity matrix. In combination with exit probabilities **R**, the **absorption matrix** is calculated as **B** = **N****R**, such that entries *B*_*ij*_ are the probability of exiting via state *j* after entering through state *i*. Let S(x) be the macrostate containing complex *x*; we can compute the probability that a single complex *x* decays into a given fate *F* (see definition 3.1) as

C 13 $$P[x\to F]=\;\{\begin{array}{cc} 1 & \mathrm{if},S(x)\;\mathrm{is}\;a\;\mathrm{resting}\;\mathrm{macrostate}\;\mathrm{and}\;F(x)=\{F\}\\ \sum_{i}B_{ij}P[r_{j}\to F] & \mathrm{if}\;S(x)\;\mathrm{is}\;a\;\mathrm{transient}\;\mathrm{macrostate}\\ 0 & \mathrm{if}F\notin F(x), \end{array}$$

where **B** = [*B*_*ij*_] is the absorption matrix for S(x), *i* represents the index of complex *x* in S(x), *j* is the index of the reaction that exits S(x), and $P\left[r_{j}\to F\right]$ is the probability that the products of the reaction *r*_*j*_ decay to complexes that represent *F*. We can calculate the **reaction decay probability** for a reaction *r*_*j*_ = (*C*, *D*) as the joint probability that products *D* = ⦃$d_{1},d_{2},\ldots ,d_{n}$⦄ decay to their respective target fates

C 14 $$P\left[r_{j}\to F\right]=\sum_{\left\{F_{1}^{\prime }+F_{2}^{\prime }+\cdots F_{n}^{\prime }=F\right\}}P\left[d_{1}\to F_{1}^{\prime }\right]\times P\left[d_{2}\to F_{2}^{\prime }\right]\times \cdots \times P\left[d_{n}\to F_{n}^{\prime }\right].$$

The sum is taken over all combinations where the fates of product complexes ⦃$F_{k}^{\prime }$⦄ sum to the overall target fate *F*. This can be computed efficiently alongside equations (3.3) and (3.4), where we compute the set of fates of a reaction using the **Cartesian sum**. First, take the **Cartesian product** of all product complex fates ⦃ *F*_*k*_′ ⦄ ∈ ⦃$\;F(d_{1})\times F(d_{2})\times \cdots :\;d_{k}\in D\;$⦄, second, take every combination where ∑_⦃ F′_*k*___⦄_ = *F*. Finally, we can write an expression for our quantity of interest. We want to know the probability $P\left[T_{B\to \hat{B}}\right]$ which can be computed using equation (C 14)

C 15 $$P\left[T_{B\to \hat{B}}\right]=P\left[r\to \hat{B}\right],$$

where *r* is the original, detailed bimolecular reaction.

Now we have shown how to efficiently compute all the terms to compute a rate constant $k_{\hat{r}}$ for each condensed reaction using equation (C 8). The structure of our arguments has mirrored the algorithm for deriving the condensed reactions, for which we provide pseudocode in electronic supplementary material, §1, Alg. 2.

## References

[1] Simmel FC, Yurke B, Singh HR 2019 Principles and applications of nucleic acid strand displacement reactions. Chem. Rev. 119, 6326–6369. (10.1021/acs.chemrev.8b00580) 30714375

[2] Wolfe BR, Pierce NA 2015 Sequence design for a test tube of interacting nucleic acid strands. ACS Synth. Biol. 4, 1086–1100. (10.1021/sb5002196) 25329866

[3] Wolfe BR, Porubsky NJ, Zadeh JN, Dirks RM, Pierce NA 2017 Constrained multistate sequence design for nucleic acid reaction pathway engineering. J. Am. Chem. Soc. 139, 3134–3144. (10.1021/jacs.6b12693) 28191938

[4] Qian L, Winfree E 2011 Scaling up digital circuit computation with DNA strand displacement cascades. Science 332, 1196–1201. (10.1126/science.1200520) 21636773

[5] Chen Y-J, Dalchau N, Srinivas N, Phillips A, Cardelli L, Soloveichik D, Seelig G 2013 Programmable chemical controllers made from DNA. Nat. Nanotechnol. 8, 755–762. (10.1038/nnano.2013.189) 24077029 PMC4150546

[6] Thubagere AJ, Thachuk C, Berleant J, Johnson RF, Ardelean DA, Cherry KM, Qian L 2017 Compiler-aided systematic construction of large-scale DNA strand displacement circuits using unpurified components. Nat. Commun. 8, 14373 (10.1038/ncomms14373) 28230154 PMC5331218

[7] Srinivas N, Parkin J, Seelig G, Winfree E, Soloveichik D 2017 Enzyme-free nucleic acid dynamical systems. Science 358, eaal2052 (10.1126/science.aal2052) 29242317

[8] Cherry KM, Qian L 2018 Scaling up molecular pattern recognition with DNA-based winner-take-all neural networks. Nature 559, 370–376. (10.1038/s41586-018-0289-6) 29973727

[9] Soloveichik D, Seelig G, Winfree E 2010 DNA as a universal substrate for chemical kinetics. Proc. Natl Acad. Sci. USA 107, 5393–5398. (10.1073/pnas.0909380107) 20203007 PMC2851759

[10] Shin SW, Thachuk C, Winfree E 2019 Verifying chemical reaction network implementations: a pathway decomposition approach. Theor. Comput. Sci. 765, 67–96. (10.1016/j.tcs.2017.10.011)

[11] Johnson R, Dong Q, Winfree E 2019 Verifying chemical reaction network implementations: a bisimulation approach. Theor. Comput. Sci. 765, 3–46. (10.1016/j.tcs.2018.01.002)

[12] Nishikawa A, Yamamura M, Hagiya M 2001 DNA computation simulator based on abstract bases. Soft Comput. 5, 25–38. (10.1007/s005000000062)

[13] Kawamata I, Tanaka F, Hagiya M 2011 Abstraction of DNA graph structures for efficient enumeration and simulation. In *Int. Conf. on Parallel and Distributed Processing Techniques and Applications*, pp. 800–806.

[14] Kawamata I, Aubert N, Hamano M, Hagiya M 2012 Abstraction of graph-based models of bio-molecular reaction systems for efficient simulation. In *Computational Methods in Systems Biology*, pp. 187–206. New York, NY: Springer (10.1007/978-3-642-33636-2_12)

[15] Phillips A, Cardelli L 2009 A programming language for composable DNA circuits. J. R. Soc. Interface 6, S419–S436. (10.1098/rsif.2009.0072.focus) 19535415 PMC2843959

[16] Lakin MR, Youssef S, Polo F, Emmott S, Phillips A 2011 Visual DSD: a design and analysis tool for DNA strand displacement systems. Bioinformatics 27, 3211–3213. (10.1093/bioinformatics/btr543) 21984756 PMC3208393

[17] Lakin MR, Youssef S, Cardelli L, Phillips A 2012 Abstractions for DNA circuit design. J. R. Soc. Interface 9, 470–486. (10.1098/rsif.2011.0343) 21775321 PMC3262419

[18] Petersen RL, Lakin MR, Phillips A 2016 A strand graph semantics for DNA-based computation. Theor. Comput. Sci. 632, 43–73. (10.1016/j.tcs.2015.07.041) 27293306 PMC4896506

[19] Mokhtar R, Garg S, Chandran H, Bui H, Song T, Reif J 2017 Modeling DNA nanodevices using graph rewrite systems. In *Advances in Unconventional Computing*, pp. 347–395. New York, NY: Springer (10.1007/978-3-319-33921-4_15)

[20] Spaccasassi C, Lakin MR, Phillips A 2019 A logic programming language for computational nucleic acid devices. ACS Synth. Biol. 8, 1530–1547. (10.1021/acssynbio.8b00229) 30372611

[21] Turner DH, Mathews DH 2010 NNDB: the nearest neighbor parameter database for predicting stability of nucleic acid secondary structure. Nucleic Acids Res. 38, D280–D282. (10.1093/nar/gkp892) 19880381 PMC2808915

[22] Lorenz R, Bernhart SH, Siederdissen CH, Tafer H, Flamm C, Stadler PF, Hofacker IL 2011 ViennaRNA Package 2.0. Algorithms Mol. Biol. 6, 26 (10.1186/1748-7188-6-26) 22115189 PMC3319429

[23] Dirks RM, Bois JS, Schaeffer JM, Winfree E, Pierce NA 2007 Thermodynamic analysis of interacting nucleic acid strands. SIAM Rev. 49, 65–88. (10.1137/060651100)

[24] Mathews DH 2014 RNA secondary structure analysis using RNAstructure. Curr. Protoc. Bioinf. 46, 12.6.1–12.6.25. (10.1002/0471250953.bi1206s46) 24939127

[25] Wetmur JG, Davidson N 1968 Kinetics of renaturation of DNA. J. Mol. Biol. 31, 349–370. (10.1016/0022-2836(68)90414-2) 5637197

[26] Wetmur JG 1976 Hybridization and renaturation kinetics of nucleic acids. Annu. Rev. Biophys. Bioeng. 5, 337–361. (10.1146/annurev.bb.05.060176.002005) 7992

[27] Wetmur JG 1991 DNA probes: applications of the principles of nucleic acid hybridization. Crit. Rev. Biochem. Mol. Biol. 26, 227–259. (10.3109/10409239109114069) 1718662

[28] Panyutin IG, Hsieh P 1993 Formation of a single base mismatch impedes spontaneous DNA branch migration. J. Mol. Biol. 230, 413–424. (10.1006/jmbi.1993.1159) 8464057

[29] Panyutin IG, Hsieh P 1994 The kinetics of spontaneous DNA branch migration. Proc. Natl Acad. Sci. USA 91, 2021–2025. (10.1073/pnas.91.6.2021) 8134343 PMC43301

[30] Guéron M, Leroy J-L. 1995 Studies of base pair kinetics by NMR measurement of proton exchange. Methods Enzymol. 261, 383–413. (10.1016/S0076-6879(95)61018-9) 8569504

[31] Bonnet G, Krichevsky O, Libchaber A 1998 Kinetics of conformational fluctuations in DNA hairpin-loops. Proc. Natl Acad. Sci. USA 95, 8602–8606. (10.1073/pnas.95.15.8602) 9671724 PMC21122

[32] Altan-Bonnet G, Libchaber A, Krichevsky O 2003 Bubble dynamics in double-stranded DNA. Phys. Rev. Lett. 90, 138101 (10.1103/PhysRevLett.90.138101) 12689326

[33] Kuznetsov SV, Ren C-C, Woodson SA, Ansari A 2008 Loop dependence of the stability and dynamics of nucleic acid hairpins. Nucleic Acids Res. 36, 1098–1112. (10.1093/nar/gkm1083) 18096625 PMC2275088

[34] Zhang DY, Winfree E 2009 Control of DNA strand displacement kinetics using toehold exchange. J. Am. Chem. Soc. 131, 17 303–17 314. (10.1021/ja906987s) 19894722

[35] Genot AJ, Zhang DY, Bath J, Turberfield AJ 2011 Remote toehold: a mechanism for flexible control of DNA hybridization kinetics. J. Am. Chem. Soc. 133, 2177–2182. (10.1021/ja1073239) 21268641

[36] Nayak RK, Peersen OB, Hall KB, Van Orden A 2012 Millisecond time-scale folding and unfolding of DNA hairpins using rapid-mixing stopped-flow kinetics. J. Am. Chem. Soc. 134, 2453–2456. (10.1021/ja208490w) 22263662

[37] Dabby NL 2013 Synthetic molecular machines for active self-assembly: prototype algorithms, designs, and experimental study. PhD thesis, California Institute of Technology, Pasadena, CA (10.7907/T0ZG-PA07)

[38] Srinivas N, Ouldridge TE, Šulc P, Schaeffer JM, Yurke B, Louis AA, Doye JPK, Winfree E 2013 On the biophysics and kinetics of toehold-mediated DNA strand displacement. Nucleic Acids Res. 41, 10 641–10 658. (10.1093/nar/gkt801) PMC3905871 24019238

[39] Tsukanov R, Tomov TE, Masoud R, Drory H, Plavner N, Liber M, Nir E 2013 Detailed study of DNA hairpin dynamics using single-molecule fluorescence assisted by DNA origami. J. Phys. Chem. B 117, 11 932–11 942. (10.1021/jp4059214) 24041226

[40] Schaeffer JM, Thachuk C, Winfree E 2015 Stochastic simulation of the kinetics of multiple interacting nucleic acid strands. In *DNA Computing and Molecular Programming*, pp. 194–211. New York, NY: Springer (10.1007/978-3-319-21999-8_13)

[41] Berleant J, Berlind C, Badelt S, Dannenberg F, Schaeffer J, Winfree E 2018 Automated sequence-level analysis of kinetics and thermodynamics for domain-level DNA strand-displacement systems. J. R. Soc. Interface 15, 20180107 (10.1098/rsif.2018.0107) 30958232 PMC6303802

[42] DNA and Natural Algorithms Group. peppercornenumerator. See www.github.com/DNA-and-Natural-Algorithms-Group/peppercornenumerator.

[43] Grun C, Werfel J, Zhang DY, Yin P 2015 DyNAMiC Workbench: an integrated development environment for dynamic DNA nanotechnology. J. R. Soc. Interface 12, 20150580 (10.1098/rsif.2015.0580) 26423437 PMC4614494

[44] Badelt S, Shin SW, Johnson RF, Dong Q, Thachuk C, Winfree E 2017 A general-purpose CRN-to-DSD compiler with formal verification, optimization, and simulation capabilities. In *DNA Computing and Molecular Programming*, pp. 232–248. New York, NY: Springer (10.1007/978-3-319-66799-7_15)

[45] Rivas E, Eddy SR 1999 A dynamic programming algorithm for RNA structure prediction including pseudoknots. J. Mol. Biol. 285, 2053–2068. (10.1006/jmbi.1998.2436) 9925784

[46] Andersen ES 2010 Prediction and design of DNA and RNA structures. New Biotechnol. 27, 184–193. (10.1016/j.nbt.2010.02.012) 20193785

[47] Doye JPK *et al.* 2013 Coarse-graining DNA for simulations of DNA nanotechnology. Phys. Chem. Chem. Phys. 15, 20 395–20 414. (10.1039/c3cp53545b) 24121860

[48] Haslinger C, Stadler PF 1999 RNA structures with pseudo-knots: graph-theoretical, combinatorial, and statistical properties. Bull. Math. Biol. 61, 437–467. (10.1006/bulm.1998.0085) 17883226 PMC7197269

[49] Dirks RM, Pierce NA 2004 An algorithm for computing nucleic acid base-pairing probabilities including pseudoknots. J. Comput. Chem. 25, 1295–1304. (10.1002/jcc.20057) 15139042

[50] Chitsaz H, Salari R, Sahinalp SC, Backofen R 2009 A partition function algorithm for interacting nucleic acid strands. Bioinformatics 25, i365–i373. (10.1093/bioinformatics/btp212) 19478011 PMC2687966

[51] Reidys CM, Huang FWD, Andersen JE, Penner RC, Stadler PF, Nebel ME 2011 Topology and prediction of RNA pseudoknots. Bioinformatics 27, 1076–1085. (10.1093/bioinformatics/btr090) 21335320

[52] Seeman NC 1982 Nucleic acid junctions and lattices. J. Theor. Biol. 99, 237–247. (10.1016/0022-5193(82)90002-9) 6188926

[53] Gillespie DT 2007 Stochastic simulation of chemical kinetics. Annu. Rev. Phys. Chem. 58, 35–55. (10.1146/annurev.physchem.58.032806.104637) 17037977

[54] Dirks RM, Pierce NA 2004 Triggered amplification by hybridization chain reaction. Proc. Natl Acad. Sci. USA 101, 15 275–15 278. (10.1073/pnas.0407024101) PMC524468 15492210

[55] Yin P, Choi HMT, Calvert CR, Pierce NA 2008 Programming biomolecular self-assembly pathways. Nature 451, 318–322. (10.1038/nature06451) 18202654

[56] Zhang DY 2011 Cooperative hybridization of oligonucleotides. J. Am. Chem. Soc. 133, 1077–1086. (10.1021/ja109089q) 21166410

[57] Cardelli L 2013 Two-domain DNA strand displacement. Math. Struct. Comput. Sci. 23, 247–271. (10.1017/S0960129512000102)

[58] Tarjan R 1972 Depth-first search and linear graph algorithms. SIAM J. Comput. 1, 146–160. (10.1137/0201010)

[59] Venkataraman S, Dirks RM, Rothemund PWK, Winfree E, Pierce NA 2007 An autonomous polymerization motor powered by DNA hybridization. Nat. Nanotechnol. 2, 490–494. (10.1038/nnano.2007.225) 18654346

[60] Lakin MR, Phillips A, Stefanovic D 2013 Modular verification of DNA strand displacement networks via serializability analysis. In *DNA Computing and Molecular Programming*, pp. 133–146. Cham, Switzerland: Springer International Publishing (10.1007/978-3-319-01928-4_10)

[61] Jones E *et al.* 2001 SciPy: Open source scientific tools for Python, 2001–. http://www.scipy.org/ (accessed 22 July 2019).

[62] SantaLucia J Jr, Hicks D 2004 The thermodynamics of DNA structural motifs. Annu. Rev. Biophys. Biomol. Struct. 33, 415–440. (10.1146/annurev.biophys.32.110601.141800) 15139820

[63] Crothers DM 1964 The kinetics of DNA denaturation. J. Mol. Biol. 9, 712–733. (10.1016/S0022-2836(64)80177-7) 14216613

[64] Manghi M, Destainville N 2016 Physics of base-pairing dynamics in DNA. Phys. Rep. 631, 1–41. (10.1016/j.physrep.2016.04.001)

[65] Kuznetsov SV, Ansari A 2012 A kinetic zipper model with intrachain interactions applied to nucleic acid hairpin folding kinetics. Biophys. J. 102, 101–111. (10.1016/j.bpj.2011.11.4017) 22225803 PMC3250679

[66] Kotani S, Hughes WL 2017 Multi-arm junctions for dynamic DNA nanotechnology. J. Am. Chem. Soc. 139, 6363–6368. (10.1021/jacs.7b00530) 28436649 PMC6317518

[67] Rohatgi A. WebPlotDigitizer. See www.github.com/ankitrohatgi/WebPlotDigitizer.

[68] Zhang DY, Turberfield AJ, Yurke B, Winfree E 2007 Engineering entropy-driven reactions and networks catalyzed by DNA. Science 318, 1121–1125. (10.1126/science.1148532) 18006742

[69] Zhang DY, Winfree E 2010 Robustness and modularity properties of a non-covalent DNA catalytic reaction. Nucleic Acids Res. 38, 4182–4197. (10.1093/nar/gkq088) 20194118 PMC2896509

[70] Faeder JR, Blinov ML, Hlavacek WS 2009 Rule-based modeling of biochemical systems with BioNetGen. In *Systems Biology*, pp. 113–167. New York, NY: Springer (10.1007/978-1-59745-525-1_5)

[71] Danos V, Feret J, Fontana W, Harmer R, Krivine J 2007 Rule-based modelling of cellular signalling. In *CONCUR 2007 - Concurrency Theory*, pp. 17–41. New York, NY: Springer (10.1007/978-3-540-74407-8_3)

[72] Andersen JL, Flamm C, Merkle D, Stadler PF 2016 A software package for chemically inspired graph transformation. In *Graph Transformation*, pp. 73–88. New York, NY: Springer (10.1007/978-3-319-40530-8_5)

[73] Zolaktaf S, Dannenberg F, Rudelis X, Condon A, Schaeffer JM, Schmidt M, Thachuk C, Winfree E 2017 Inferring parameters for an elementary step model of DNA structure kinetics with locally context-dependent Arrhenius rates. In *DNA Computing and Molecular Programming*, pp. 172–187. New York, NY: Springer (10.1007/978-3-319-66799-7_12)

[74] Peleš S, Munsky B, Khammash M 2006 Reduction and solution of the chemical master equation using time scale separation and finite state projection. J. Chem. Phys. 125, 204104 (10.1063/1.2397685) 17144687

[75] Kuwahara H, Myers CJ, Samoilov MS, Barker NA, Arkin AP 2006 Automated abstraction methodology for genetic regulatory networks. In *Transactions on Computational Systems Biology VI*, pp. 150–175. New York, NY: Springer (10.1007/11880646_7)

[76] Madelaine G, Lhoussaine C, Niehren J 2015 Structural simplification of chemical reaction networks preserving deterministic semantics. In *Computational Methods in Systems Biology*, pp. 133–144. New York, NY: Springer (10.1007/978-3-319-23401-4_12) 27521766

[77] Thachuk C, Winfree E, Soloveichik D 2015 Leakless DNA strand displacement systems. In *DNA Computing and Molecular Programming*, pp. 133–153. New York, NY: Springer (10.1007/978-3-319-21999-8_9)

[78] Wang B, Thachuk C, Ellington AD, Winfree E, Soloveichik D 2018 Effective design principles for leakless strand displacement systems. Proc. Natl Acad. Sci. USA 115, E12182–E12191. (10.1073/pnas.1806859115) 30545914 PMC6310779

[79] Flamm C, Fontana W, Hofacker IL, Schuster P 2000 RNA folding at elementary step resolution. RNA 6, 325–338. (10.1017/S1355838200992161) 10744018 PMC1369916

[80] Ouldridge TE, Šulc P, Romano F, Doye JPK, Louis AA 2013 DNA hybridization kinetics: zippering, internal displacement and sequence dependence. Nucleic Acids Res. 41, 8886–8895. (10.1093/nar/gkt687) 23935069 PMC3799446

