# Supplementary material for: A domain-level DNA strand displacement reaction enumerator allowing arbitrary non-pseudoknotted secondary structures
Source: J R Soc Interface. 2020 Jun 3;17(167):20190866. doi: 10.1098/rsif.2019.0866 (PMC7328391; doi:10.1098/rsif.2019.0866)
Supplement: Supplemental Online Material [file rsif20190866supp1.pdf]

A domain-level DNA strand displacement reaction enumerator allowing arbitrary non-pseudoknotted secondary structures

Stefan Badelt[1*], Casey Grun[2†*], Karthik V. Sarma[3], Brian Wolfe[1], Seung Woo Shin[1], and Erik Winfree[1‡]

[1] California Institute of Technology, Pasadena, CA, USA
[2] Wyss Institute, Harvard University, Boston, MA, USA
[3] David Geffen School of Medicine at UCLA, Los Angeles, CA, USA

Supplemental Online Material

1 Reaction enumeration algorithm details

The reaction enumeration algorithm works by passing complexes through a progression of several mutable sets; as new complexes are enumerated, they are added to one of these sets, then eventually removed and transferred to a later set. All complexes accumulate in either $\mathcal{T}$ (transient complexes) or $\mathcal{E}$ (resting complexes). This progression enforces the requirement that each complex be classified as a resting complex or transient complex, that all fast reactions are enumerated before slow reactions, and that complexes are not enumerated more than once. For simplicity, we assume an operation $\textsc{Pop}(S)$ exists that removes and returns some element from the mutable set S. In Alg. 1 we provide pseudocode for the reaction enumeration algorithm.

- $\mathcal{B}$ contains complexes that have had no reactions enumerated yet. Complexes are moved out of $\mathcal{B}$ into $\mathcal{F}$ when their **neighborhood** is considered. We define the **neighborhood** of a complex c to be the set of complexes that can be produced by a series of zero or more fast reactions starting from complex c.
- $\mathcal{F}$ contains complexes in the current neighborhood which have not yet had fast reactions enumerated. These complexes will be moved to $\mathcal{N}$ once their fast reactions have been enumerated.
- $\mathcal{N}$ contains complexes enumerated within the current neighborhood, but that have not yet been characterized as transient or resting complexes. Each of these complexes is classified, then moved into $\mathcal{S}$ or $\mathcal{T}$.
- $\mathcal{S}$ contains resting complexes which have not yet had bimolecular reactions with set $\mathcal{E}$ enumerated yet. All self-interactions for these complexes have been enumerated.
- $\mathcal{E}$ contains enumerated **resting complexes**. Only cross-reactions with other end states need to be considered for these complexes. These complexes will remain in this list throughout function execution.
- $\mathcal{T}$ contains **transient complexes** which have had their fast reactions enumerated. These complexes will remain in this list throughout function execution.

Additionally, two other sets are accumulated over the course of the enumeration:

- $\mathcal{R}$ contains all reactions that have been enumerated.
- $\mathcal{Q}$ contains all resting macrostates that have been enumerated.

1.1 Terminating conditions

Although our enumerator is designed to avoid enumerating implausible polymerization reactions, as described in Fig. 2, it is possible to enumerate systems which result in genuine polymerization, such as those described by [1,2]. To allow such enumerations to terminate, our enumerator places a soft limit on the maximum number of complexes and the maximum number of reactions that can be enumerated before the enumerator will exit. These limits are checked before the neighborhood of fast reactions is enumerated, for each complex in B. The limits are configurable by the user.

* These authors contributed equally to this study.
† current address: Yale University, New Haven, CT, USA
‡ To whom correspondence should be addressed, winfree@caltech.edu

If the number of complexes in $\mathcal{E} \cup \mathcal{S} \cup \mathcal{T}$ is greater than the maximum number of complexes, or the number of reactions in $\mathcal{R}$ is greater than the configured maximum, the partially-enumerated network is "cleaned up" by deleting all reactions in $\mathcal{R}$ that produce complex(es) leftover in $\mathcal{B}$. That is, no complex will be reported in the output that has not had its neighborhood of fast reactions enumerated exhaustively. Evaluating the limits only *between* consideration of each neighborhood (rather than during) prevents the pathological mis-classification of resting and transient complexes, but means the limit may be exceeded if the last neighborhood considered is large.

1.2 Pseudocode

Algorithm 1 Reaction enumeration

procedure ENUMERATE(A : {Complex})
$\mathcal{E} \leftarrow \{\}\,; \mathcal{S} \leftarrow \{\}\,; \mathcal{T} \leftarrow \{\}$ ▷ Complexes
$\mathcal{R} \leftarrow \{\}$ ▷ Reactions
$\mathcal{Q} \leftarrow \{\}$ ▷ Resting macrostates
$\mathcal{B} \leftarrow A$
while $\mathcal{B} \neq \{\}$ **do** ▷ Enumerate fast reactions from A
$b \leftarrow \text{pop}(\mathcal{B})$
$(S', T', Q', R') \leftarrow$ ENUMERATENEIGHBORHOOD(b) ▷ Find fast reactions from b
$\mathcal{S} \leftarrow \mathcal{S} \cup S'$; $\mathcal{T} \leftarrow \mathcal{T} \cup T'$; $\mathcal{R} \leftarrow \mathcal{R} \cup R'$; $\mathcal{Q} \leftarrow \mathcal{Q} \cup Q'$
end while
while $\mathcal{S} \neq \{\}$ **do** ▷ Enumerate slow reactions between resting complexes
$s \leftarrow \text{pop}(\mathcal{S})$
$(R', B') \leftarrow$ GETSLOWREACTIONS(s, $\mathcal{S} \cup \mathcal{E}$) ▷ Find slow reactions from s
$\mathcal{E} \leftarrow \mathcal{E} \cup \{s\}$ ▷ s moves to $\mathcal{E}$ once slow reactions are enumerated
$\mathcal{R} \leftarrow \mathcal{R} \cup R'$ ▷ Store new reactions
$\mathcal{B} \leftarrow \mathcal{B} \cup B' \setminus (\mathcal{E} \cup \mathcal{S} \cup T)$ ▷ Store new complexes
while $\mathcal{B} \neq \{\}$ **do** ▷ Enumerate fast reactions from B
$b \leftarrow \text{pop}(\mathcal{B})$
$(S', T', Q', R') \leftarrow$ ENUMERATENEIGHBORHOOD(b) ▷ Find fast reactions from b
$\mathcal{S} \leftarrow \mathcal{S} \cup S'$; $\mathcal{T} \leftarrow \mathcal{T} \cup T'$; $\mathcal{R} \leftarrow \mathcal{R} \cup R'$; $\mathcal{Q} \leftarrow \mathcal{Q} \cup Q'$
end while
end while
end procedure

procedure ENUMERATENEIGHBORHOOD(c : Complex) ▷ Calculates fast reactions from c, sorts complexes into resting/transient complexes
$\mathcal{F} = \{c\}$ ▷ Complexes from fast reactions in neighborhood
$\mathcal{N} = \{\}$ ▷ Complexes in neighborhood
$\mathcal{R}_N = \{\}$ ▷ (Fast) Reactions in neighborhood
while $\mathcal{F} \neq \{\}$ **do** ▷ Enumerate fast reactions from each complex in F
$f \leftarrow \text{pop}(\mathcal{F})$
$(R', F') \leftarrow$ GETFASTREACTIONS(f) ▷ Find fast reactions from f
$\mathcal{F} \leftarrow \mathcal{F} \cup F' \setminus \mathcal{N}$
$\mathcal{N} \leftarrow \mathcal{N} \cup F'$
$\mathcal{R}_N \leftarrow \mathcal{R}_N \cup R'$
end while
Apply Tarjan's algorithm [3] to find strongly-connected components of the directed graph $G = (\mathcal{N}, \mathcal{R}_N)$
$Q' \leftarrow$ {strongly-connected components of G with no outgoing fast reactions} ▷ Resting macrostates are SCCs of G
$S' \leftarrow \{s : s \in q \text{ for any } q \in Q'\}$ ▷ resting complexes are in a resting macrostate
$T' \leftarrow \mathcal{N} \setminus S'$ ▷ Transient complexes are everything else
return $(S', T', Q', \mathcal{R}_N)$
end procedure

procedure GETFASTREACTIONS(c : Complex) ▷ Calculates all fast (unimolecular) reactions that consume c
$R \leftarrow$ fast reactions consuming c, $C \leftarrow$ union of products of reactions in R
return R, C
end procedure
procedure GETSLOWREACTIONS(c : Complex, S : {Complex}) ▷ Calculates all slow (bimolecular) reactions that consume c and an element of S
$R \leftarrow$ slow reactions consuming c and $s \in S$, $C \leftarrow$ union of products of reactions in R
return R, C
end procedure

Algorithm 2 Reaction network condensation

$\mathbb{F}x \leftarrow$ undefined $\forall$ complexes x ▷ The map $\mathbb{F}$: Complex $\rightarrow$ {Fate} is global and begins empty
$S \leftarrow \{\ \}$ ▷ The map S : Complex $\rightarrow$ {Complex} is global and begins empty

procedure CONDENSE($\mathcal{C}$: {Complex}, $\mathcal{R}$: {Reaction}) ▷ Computes the fates for each complex, then generates a set of condensed reactions
  $\mathcal{R}_s \leftarrow \{r : r \in \mathcal{R}, r$ is slow$\}$ ▷ Slow reactions
  $\mathcal{R}_f \leftarrow \mathcal{R} \setminus \mathcal{R}_s$ ▷ Fast reactions
  $\mathcal{R}_f^{(1,1)} \leftarrow \{r \in \mathcal{R}_f : \alpha(r) = (1,1)\}$ ▷ Fast (1,1) reactions
  Use Tarjan's algorithm [3] to compute the set of strongly-connected components from the graph $\Gamma = (\mathcal{C}, \mathcal{R}_f^{(1,1)})$
  $S \leftarrow$ the set of strongly-connected components of Γ
  $\mathbb{S}(x) \leftarrow$ the strongly-connected component containing complex x, $\forall x \in \mathcal{C}$
  for all $\mathcal{C}_c \in S$ **do** ▷ For each SCC $\mathcal{C}_c$ of Γ
    COMPUTEFATES(C_c, $\mathcal{R}_f$)
  end for
  return CONDENSEREACTIONS($\mathcal{R}_s$)
end procedure

procedure COMPUTEFATES($\mathcal{C}$: {Complex}, $\mathcal{R}_f$: {Reaction})
  $R_o \leftarrow \{r = (A,B) \in \mathcal{R}_f : A \subseteq \mathcal{C}, B \setminus \mathcal{C} \neq \emptyset\}$ ▷ Outgoing fast reactions
  if $|R_o| = 0$ **then** ▷ If no outgoing fast reactions
    $\mathbb{F}(c) \leftarrow \{\!\{\mathcal{C}\}\!\}\ \forall\ c \in \mathcal{C}$ ▷ $\mathbb{F}(c)$ is the resting macrostate $\mathcal{C}$ containing the complex c
  else ▷ If there are outgoing fast reactions
    for all $c \in \mathcal{C}$ **do**
      $R_o^{(}1,n) \leftarrow \{r \in R_o : \alpha(r) = (1,n)\}$
      $P_o \leftarrow \bigcup_{r=(A,B)\in R_o^{(1,1)}} B$
      for all $x \in P_o$ **do**
        If $\mathbb{F}(x)$ is undefined, COMPUTEFATES($\mathbb{S}(x)$, $\mathcal{R}_f$)
      end for
      $\mathbb{F}(c) \leftarrow \bigcup_{r=(A,B)\in R_o} \left(\bigoplus_{b\in B} \mathbb{F}(b) \right)$ ▷ $\mathbb{F}(c)$ are the possible fates from outgoing reactions
    end for
  end if
end procedure

procedure CONDENSEREACTIONS($\mathcal{R}_s$: {Reaction}) ▷ Condensed reaction space from the set of slow reactions
  $\hat{\mathcal{R}} \leftarrow \{\ \}$ ▷ Condensed reactions
  for all $s = (A,b) \in \mathcal{R}_s$ **do**
    $A' \leftarrow \sum_{a\in A} \mathbb{F}(a)$ ▷ Fates of reactants are all trivial
    for all $B' \in \mathbb{F}(b)$ **do** ▷ For each fate of b
      $r' \leftarrow (A', B')$ ▷ Generate new reaction
      $\hat{\mathcal{R}} \leftarrow \hat{\mathcal{R}} \cup r'$
    end for
  end for
  return $\hat{\mathcal{R}}$
end procedure

2 Case study raw data

Input Filename	(n, m)	Reaction	Semantics	Rate (calculated)	Rate (experiment)
Zhang2009-3way-00	(1, 14)	S + X → L + Y	condensed	11.891	8.170
Zhang2009-3way-01	(2, 13)	S + X → L + Y	condensed	209.564	144.000
Zhang2009-3way-02	(3, 12)	S + X → L + Y	condensed	3680.666	1080.000
Zhang2009-3way-03	(4, 11)	S + X → L + Y	condensed	61798.887	50500.000
Zhang2009-3way-04	(5, 10)	S + X → L + Y	condensed	650503.785	964000.000
Zhang2009-3way-05	(6, 9)	S + X → L + Y	condensed	1653058.538	2360000.000
Zhang2009-3way-06	(7, 8)	S + X → L + Y	condensed	2087718.874	3220000.000
Zhang2009-3way-07	(8, 9)	S + X → L + Y	condensed	2400000.000	3150000.000
Zhang2009-3way-08	(9, 8)	S + X → L + Y	condensed	2700000.000	2770000.000
Zhang2009-3way-09	(10, 7)	S + X → L + Y	condensed	3000000.000	2830000.000
Zhang2009-3way-10	(15, 0)	S + X → L + Y	condensed	4500000.000	4780000.000

Table 1: Data for Fig. 7 a. Zhang and Winfree (2009) – 3-way strand displacement

Input Filename	(n, m)	Reaction	Semantics	Rate (calculated)	Rate (experiment)
Zhang2009-3wayX-00	(1, 4)	S + X → L + Y	condensed	13.919	7.700
Zhang2009-3wayX-01	(1, 3)	S + X → L + Y	condensed	13.922	5.480
Zhang2009-3wayX-02	(1, 2)	S + X → L + Y	condensed	13.207	23.500
Zhang2009-3wayX-03	(1, 1)	S + X → L + Y	condensed	12.516	18.900
Zhang2009-3wayX-04	(2, 5)	S + X → L + Y	condensed	138.274	43.600
Zhang2009-3wayX-05	(2, 4)	S + X → L + Y	condensed	245.292	214.050
Zhang2009-3wayX-06	(2, 3)	S + X → L + Y	condensed	245.346	273.000
Zhang2009-3wayX-07	(2, 2)	S + X → L + Y	condensed	232.750	249.000
Zhang2009-3wayX-08	(2, 1)	S + X → L + Y	condensed	220.581	231.000
Zhang2009-3wayX-09	(3, 6)	S + X → L + Y	condensed	309.170	66.900
Zhang2009-3wayX-10	(3, 5)	S + X → L + Y	condensed	2431.660	215.000
Zhang2009-3wayX-11	(3, 4)	S + X → L + Y	condensed	4305.421	939.000
Zhang2009-3wayX-12	(3, 3)	S + X → L + Y	condensed	4306.368	974.000
Zhang2009-3wayX-13	(3, 2)	S + X → L + Y	condensed	4086.192	907.000
Zhang2009-3wayX-14	(3, 1)	S + X → L + Y	condensed	3873.391	846.000
Zhang2009-3wayX-15	(4, 7)	S + X → L + Y	condensed	381.692	131.000
Zhang2009-3wayX-16	(4, 6)	S + X → L + Y	condensed	5427.681	407.000
Zhang2009-3wayX-17	(4, 5)	S + X → L + Y	condensed	41498.277	4250.000
Zhang2009-3wayX-18	(4, 4)	S + X → L + Y	condensed	71709.175	21300.000
Zhang2009-3wayX-19	(4, 3)	S + X → L + Y	condensed	71724.079	24100.000
Zhang2009-3wayX-20	(4, 2)	S + X → L + Y	condensed	68249.766	22900.000
Zhang2009-3wayX-21	(4, 1)	S + X → L + Y	condensed	64873.056	19700.000
Zhang2009-3wayX-22	(5, 7)	S + X → L + Y	condensed	6701.066	3590.000
Zhang2009-3wayX-23	(5, 6)	S + X → L + Y	condensed	90332.748	97200.000
Zhang2009-3wayX-24	(5, 5)	S + X → L + Y	condensed	503452.200	345000.000
Zhang2009-3wayX-25	(5, 4)	S + X → L + Y	condensed	709009.167	1530000.000
Zhang2009-3wayX-26	(5, 3)	S + X → L + Y	condensed	709091.806	1580000.000
Zhang2009-3wayX-27	(5, 2)	S + X → L + Y	condensed	689410.828	1580000.000
Zhang2009-3wayX-28	(5, 1)	S + X → L + Y	condensed	669445.127	1730000.000
Zhang2009-3wayX-29	(6, 7)	S + X → L + Y	condensed	111326.440	161000.000
Zhang2009-3wayX-30	(6, 6)	S + X → L + Y	condensed	872844.303	405000.000
Zhang2009-3wayX-31	(6, 5)	S + X → L + Y	condensed	1586271.727	1480000.000
Zhang2009-3wayX-32	(6, 4)	S + X → L + Y	condensed	1672957.506	3040000.000
Zhang2009-3wayX-33	(6, 3)	S + X → L + Y	condensed	1672983.602	2590000.000
Zhang2009-3wayX-34	(6, 2)	S + X → L + Y	condensed	1666616.282	3000000.000
Zhang2009-3wayX-35	(7, 7)	S + X → L + Y	condensed	1047996.408	470000.000
Zhang2009-3wayX-36	(7, 6)	S + X → L + Y	condensed	1962075.431	1110000.000
Zhang2009-3wayX-37	(7, 5)	S + X → L + Y	condensed	2081440.832	2900000.000
Zhang2009-3wayX-38	(7, 4)	S + X → L + Y	condensed	2089499.345	3570000.000
Zhang2009-3wayX-39	(8, 7)	S + X → L + Y	condensed	2400000.000	1940000.000
Zhang2009-3wayX-40	(8, 6)	S + X → L + Y	condensed	2400000.000	2680000.000
Zhang2009-3wayX-41	(8, 5)	S + X → L + Y	condensed	2400000.000	3140000.000
Zhang2009-3wayX-42	(8, 4)	S + X → L + Y	condensed	2400000.000	3370000.000

Table 2: Data for Fig. 7 b. Zhang and Winfree (2009) – 3-way toehold exchange

Input Filename	(n, m)	Reaction	Semantics	Rate (calculated)	Rate (experiment)
Dabby2013-4way-00	(0, 2)	clx + rep → pr1 + pr2	condensed	0.006	0.047
Dabby2013-4way-01	(2, 2)	clx + rep → pr1 + pr2	condensed	0.014	0.100
Dabby2013-4way-02	(2, 0)	clx + rep → pr1 + pr2	condensed	0.006	0.033
Dabby2013-4way-03	(4, 2)	clx + rep → pr1 + pr2	condensed	2.675	0.930
Dabby2013-4way-04	(4, 0)	clx + rep → pr1 + pr2	condensed	1.740	0.039
Dabby2013-4way-05	(0, 4)	clx + rep → pr1 + pr2	condensed	1.740	0.970
Dabby2013-4way-06	(2, 4)	clx + rep → pr1 + pr2	condensed	2.675	56.000
Dabby2013-4way-07	(6, 2)	clx + rep → pr1 + pr2	condensed	828.046	490.000
Dabby2013-4way-08	(0, 6)	clx + rep → pr1 + pr2	condensed	540.548	58.000
Dabby2013-4way-09	(4, 4)	clx + rep → pr1 + pr2	condensed	151.477	770.000
Dabby2013-4way-10	(6, 0)	clx + rep → pr1 + pr2	condensed	540.548	5.000
Dabby2013-4way-11	(2, 6)	clx + rep → pr1 + pr2	condensed	828.046	9400.000
Dabby2013-4way-12	(4, 6)	clx + rep → pr1 + pr2	condensed	39523.467	70000.000
Dabby2013-4way-13	(6, 4)	clx + rep → pr1 + pr2	condensed	39523.467	280000.000
Dabby2013-4way-14	(6, 6)	clx + rep → pr1 + pr2	condensed	2609291.671	690000.000

Table 3: Data for Fig. 7 c. Dabby (2013) – 4-way strand displacement

Input Filename	Simulation	Reporter	Metric	Semantics	Concentration (simulation)	Time (simulation)	Concentration (experiment)	Time (experiment)
Genot2011-F3-00	S=6.6 I=660	T	completion-time	condensed, k_fast=0.01, k_slow=1e-10	1.98	51.8	1.98	42
Genot2011-F3-01	S=6.6 I=660	T	completion-time	condensed, k_fast=0.01, k_slow=1e-10	1.98	248	1.98	48
Genot2011-F3-02	S=6.6 I=660	T	completion-time	condensed, k_fast=0.01, k_slow=1e-10	1.98	350	1.98	100
Genot2011-F3-03	S=6.6 I=660	T	completion-time	condensed, k_fast=0.01, k_slow=1e-10	1.98	474	1.98	91
Genot2011-F4A-00	S=6.6 I=22	T	completion-time	condensed, k_fast=0.01, k_slow=1e-10	1.98	5.2	1.98	2
Genot2011-F4A-01	S=6.6 I=22	T	completion-time	condensed, k_fast=0.01, k_slow=1e-10	1.98	6.4	1.98	3
Genot2011-F4B-00	S=6.6 I=22	T	completion-time	condensed, k_fast=0.01, k_slow=1e-10	1.98	5.2	1.98	15
Genot2011-F4B-01	S=6.6 I=22	T	completion-time	condensed, k_fast=0.01, k_slow=1e-10	1.98	6.4	1.98	75
Genot2011-F4C-00	S=6.6 I=330	T	completion-time	condensed, k_fast=0.01, k_slow=1e-10	1.98	0.7	1.98	25
Genot2011-F4C-00	S=6.6 I=145	T	completion-time	condensed, k_fast=0.01, k_slow=1e-10	1.98	1.6	1.98	75
Genot2011-F4C-00	S=6.6 I=66	T	completion-time	condensed, k_fast=0.01, k_slow=1e-10	1.98	3.4	1.98	150
Genot2011-F4D-00	S=6.6 I=330	T	completion-time	condensed, k_fast=0.01, k_slow=1e-10	1.98	249	1.98	51
Genot2011-F4D-00	S=6.6 I=145	T	completion-time	condensed, k_fast=0.01, k_slow=1e-10	1.98	250	1.98	55
Genot2011-F4D-00	S=6.6 I=66	T	completion-time	condensed, k_fast=0.01, k_slow=1e-10	1.98	252	1.98	60
Genot2011-SF4A-00	R=30 X=40 S=10	F	completion-time	condensed, k_fast=0.01, k_slow=1e-10	3	273	3	4249
Genot2011-SF4A-01	R=30 X=40 S=10	F	completion-time	condensed, k_fast=0.01, k_slow=1e-10	3	375	3	6492
Genot2011-SF4A-02	R=30 X=40 S=10	F	completion-time	condensed, k_fast=0.01, k_slow=1e-10	3	499	3	12157

Table 4: Data for Fig. 7 d. Genot et al. (2011) – remote-toehold strand displacement

Input Filename	Simulation	Reporter	Metric	Metric-values	Semantics	Concentration (simulation)	Time (simulation)	Concentration (experiment)	Time (experiment)
Kotani2017-F4-00	C1=0.1	D	completion-time	5	detailed, release_cutoff=8	5	7176	5	6132
Kotani2017-F4-00	C1=0.01	D	completion-time	5	detailed, release_cutoff=8	5	10217	5	9125
Kotani2017-F4-00	C1=0.001	D	completion-time	5	detailed, release_cutoff=8	5	13269	5	10780
Kotani2017-F4-00	C1=0	D	completion-time	5	detailed, release_cutoff=8	5	32366	5	11520
Kotani2017-F4-00	C1=0.1	D	completion-time	5	condensed, release_cutoff=8	5	500	5	6132
Kotani2017-F4-00	C1=0.01	D	completion-time	5	condensed, release_cutoff=8	5	736	5	9125
Kotani2017-F4-00	C1=0.001	D	completion-time	5	condensed, release_cutoff=8	5	972	5	10780
Kotani2017-F4-00	C1=0	D	completion-time	5	condensed, release_cutoff=8	5	2248	5	11520
Kotani2017-F4-00	C1=0.1	D	completion-time	5	detailed, k_slow=0.0001	5	7176	5	6132
Kotani2017-F4-00	C1=0.01	D	completion-time	5	detailed, k_slow=0.0001	5	10217	5	9125
Kotani2017-F4-00	C1=0.001	D	completion-time	5	detailed, k_slow=0.0001	5	13269	5	10780
Kotani2017-F4-00	C1=0	D	completion-time	5	detailed, k_slow=0.0001	5	32538	5	11520
Kotani2017-F4-00	C1=0.1	D	completion-time	5	condensed, k_slow=0.0001	5	500	5	6132
Kotani2017-F4-00	C1=0.01	D	completion-time	5	condensed, k_slow=0.0001	5	736	5	9125
Kotani2017-F4-00	C1=0.001	D	completion-time	5	condensed, k_slow=0.0001	5	972	5	10780
Kotani2017-F4-00	C1=0	D	completion-time	5	condensed, k_slow=0.0001	5	2244	5	11520
Kotani2017-F4-00	C1=0.1	D	completion-time	5	detailed, k_slow=0.0001, k_fast=0.001	5	10577	5	6132
Kotani2017-F4-00	C1=0.01	D	completion-time	5	detailed, k_slow=0.0001, k_fast=0.001	5	15137	5	9125
Kotani2017-F4-00	C1=0.001	D	completion-time	5	detailed, k_slow=0.0001, k_fast=0.001	5	19777	5	10780
Kotani2017-F4-00	C1=0	D	completion-time	5	detailed, k_slow=0.0001, k_fast=0.001	5	nan	5	11520
Kotani2017-F4-00	C1=0.1	D	completion-time	5	condensed, k_slow=0.0001, k_fast=0.001	5	4680	5	6132
Kotani2017-F4-00	C1=0.01	D	completion-time	5	condensed, k_slow=0.0001, k_fast=0.001	5	6084	5	9125
Kotani2017-F4-00	C1=0.001	D	completion-time	5	condensed, k_slow=0.0001, k_fast=0.001	5	7504	5	10780
Kotani2017-F4-00	C1=0	D	completion-time	5	condensed, k_slow=0.0001, k_fast=0.001	5	nan	5	11520
Kotani2017-F4-00	C1=0.1	D	completion-time	5	detailed, k_slow=0.0001, k_fast=0.01	5	8244	5	6132
Kotani2017-F4-00	C1=0.01	D	completion-time	5	detailed, k_slow=0.0001, k_fast=0.01	5	10977	5	9125
Kotani2017-F4-00	C1=0.001	D	completion-time	5	detailed, k_slow=0.0001, k_fast=0.01	5	13737	5	10780
Kotani2017-F4-00	C1=0	D	completion-time	5	detailed, k_slow=0.0001, k_fast=0.01	5	nan	5	11520
Kotani2017-F4-00	C1=0.1	D	completion-time	5	condensed, k_slow=0.0001, k_fast=0.01	5	5656	5	6132
Kotani2017-F4-00	C1=0.01	D	completion-time	5	condensed, k_slow=0.0001, k_fast=0.01	5	7520	5	9125
Kotani2017-F4-00	C1=0.001	D	completion-time	5	condensed, k_slow=0.0001, k_fast=0.01	5	9401	5	10780
Kotani2017-F4-00	C1=0	D	completion-time	5	condensed, k_slow=0.0001, k_fast=0.01	5	nan	5	11520
Kotani2017-F4-00	C1=0.1	D	completion-time	5	detailed, k_slow=1e-05, k_fast=0.01	5	8244	5	6132
Kotani2017-F4-00	C1=0.01	D	completion-time	5	detailed, k_slow=1e-05, k_fast=0.01	5	10977	5	9125
Kotani2017-F4-00	C1=0.001	D	completion-time	5	detailed, k_slow=1e-05, k_fast=0.01	5	13737	5	10780
Kotani2017-F4-00	C1=0	D	completion-time	5	detailed, k_slow=1e-05, k_fast=0.01	5	nan	5	11520
Kotani2017-F4-00	C1=0.1	D	completion-time	5	condensed, k_slow=1e-05, k_fast=0.01	5	5656	5	6132
Kotani2017-F4-00	C1=0.01	D	completion-time	5	condensed, k_slow=1e-05, k_fast=0.01	5	7520	5	9125
Kotani2017-F4-00	C1=0.001	D	completion-time	5	condensed, k_slow=1e-05, k_fast=0.01	5	9401	5	10780
Kotani2017-F4-00	C1=0	D	completion-time	5	condensed, k_slow=1e-05, k_fast=0.01	5	nan	5	11520

Table 5: Data for Fig. 8. Kotani & Hughes (2017) – Autocatalytic system

Input Filename	Simulation	Reporter	Metric	Semantics	Concentration (sim)	Time (sim)	Concentration (exp)	Time (exp)
Qian2011-SF22-00	I=100	O	diagonal-crossing-time:2700:100	condensed, k_fast=1, k_slow=0.01	95	139	72.9	739
Qian2011-SF22-00	I=90	O	diagonal-crossing-time:2700:100	condensed, k_fast=1, k_slow=0.01	94.6	146	71.8	766
Qian2011-SF22-00	I=80	O	diagonal-crossing-time:2700:100	condensed, k_fast=1, k_slow=0.01	94.3	156	70.6	797
Qian2011-SF22-00	I=70	O	diagonal-crossing-time:2700:100	condensed, k_fast=1, k_slow=0.01	93.8	168	69.2	835
Qian2011-SF22-00	I=60	O	diagonal-crossing-time:2700:100	condensed, k_fast=1, k_slow=0.01	93.3	184	67.2	886
Qian2011-SF22-00	I=50	O	diagonal-crossing-time:2700:100	condensed, k_fast=1, k_slow=0.01	92.5	205	65	952
Qian2011-SF22-00	I=40	O	diagonal-crossing-time:2700:100	condensed, k_fast=1, k_slow=0.01	91.3	234	61.8	1.04e+03
Qian2011-SF22-00	I=30	O	diagonal-crossing-time:2700:100	condensed, k_fast=1, k_slow=0.01	89.7	280	57.3	1.16e+03
Qian2011-SF22-00	I=20	O	diagonal-crossing-time:2700:100	condensed, k_fast=1, k_slow=0.01	86.7	360	50.3	1.35e+03
Qian2011-SF22-00	I=10	O	diagonal-crossing-time:2700:100	condensed, k_fast=1, k_slow=0.01	79.8	547	37.5	1.7e+03
Qian2011-SF22-00	I=100	O	diagonal-crossing-time:2700:100	condensed, dG_bp=-1.3, k_fast=1, k_slow=0.01	73.7	710	72.9	739
Qian2011-SF22-00	I=90	O	diagonal-crossing-time:2700:100	condensed, dG_bp=-1.3, k_fast=1, k_slow=0.01	72.7	737	71.8	766
Qian2011-SF22-00	I=80	O	diagonal-crossing-time:2700:100	condensed, dG_bp=-1.3, k_fast=1, k_slow=0.01	71.5	770	70.6	797
Qian2011-SF22-00	I=70	O	diagonal-crossing-time:2700:100	condensed, dG_bp=-1.3, k_fast=1, k_slow=0.01	70	810	69.2	835
Qian2011-SF22-00	I=60	O	diagonal-crossing-time:2700:100	condensed, dG_bp=-1.3, k_fast=1, k_slow=0.01	68.2	860	67.2	886
Qian2011-SF22-00	I=50	O	diagonal-crossing-time:2700:100	condensed, dG_bp=-1.3, k_fast=1, k_slow=0.01	65.8	924	65	952
Qian2011-SF22-00	I=40	O	diagonal-crossing-time:2700:100	condensed, dG_bp=-1.3, k_fast=1, k_slow=0.01	62.6	1.01e+03	61.8	1.04e+03
Qian2011-SF22-00	I=30	O	diagonal-crossing-time:2700:100	condensed, dG_bp=-1.3, k_fast=1, k_slow=0.01	58.1	1.13e+03	57.3	1.16e+03
Qian2011-SF22-00	I=20	O	diagonal-crossing-time:2700:100	condensed, dG_bp=-1.3, k_fast=1, k_slow=0.01	51.1	1.32e+03	50.3	1.35e+03
Qian2011-SF22-00	I=10	O	diagonal-crossing-time:2700:100	condensed, dG_bp=-1.3, k_fast=1, k_slow=0.01	38.2	1.67e+03	37.5	1.7e+03
Qian2011-SF23-00	I=100	O	diagonal-crossing-time:10800:100	condensed, k_fast=1, k_slow=0.01	97.1	310	83.2	1.81e+03
Qian2011-SF23-00	I=90	O	diagonal-crossing-time:10800:100	condensed, k_fast=1, k_slow=0.01	96.6	366	80.7	2.08e+03
Qian2011-SF23-00	I=80	O	diagonal-crossing-time:10800:100	condensed, k_fast=1, k_slow=0.01	95.8	458	76.3	2.59e+03
Qian2011-SF23-00	I=70	O	diagonal-crossing-time:10800:100	condensed, k_fast=1, k_slow=0.01	94.1	640	67.4	3.52e+03
Qian2011-SF23-00	I=60	O	diagonal-crossing-time:10800:100	condensed, k_fast=1, k_slow=0.01	88.4	1.25e+03	39.1	6.59e+03
Qian2011-SF23-00	I=50	O	diagonal-crossing-time:10800:100	condensed, k_fast=1, k_slow=0.01	32.7	7.27e+03	9.05	9.83e+03
Qian2011-SF23-00	I=40	O	diagonal-crossing-time:10800:100	condensed, k_fast=1, k_slow=0.01	19.3	8.71e+03	6.21	1.01e+04
Qian2011-SF23-00	I=30	O	diagonal-crossing-time:10800:100	condensed, k_fast=1, k_slow=0.01	12.3	9.48e+03	4.41	1.03e+04
Qian2011-SF23-00	I=20	O	diagonal-crossing-time:10800:100	condensed, k_fast=1, k_slow=0.01	7.25	1e+04	2.47	1.05e+04
Qian2011-SF23-00	I=10	O	diagonal-crossing-time:10800:100	condensed, k_fast=1, k_slow=0.01	3.3	1.04e+04	1.31	1.06e+04
Qian2011-SF23-00	I=100	O	diagonal-crossing-time:10800:100	condensed, dG_bp=-1.3, k_fast=1, k_slow=0.01	83.9	1.74e+03	83.2	1.81e+03
Qian2011-SF23-00	I=90	O	diagonal-crossing-time:10800:100	condensed, dG_bp=-1.3, k_fast=1, k_slow=0.01	81.7	1.98e+03	80.7	2.08e+03
Qian2011-SF23-00	I=80	O	diagonal-crossing-time:10800:100	condensed, dG_bp=-1.3, k_fast=1, k_slow=0.01	78.3	2.35e+03	76.3	2.59e+03
Qian2011-SF23-00	I=70	O	diagonal-crossing-time:10800:100	condensed, dG_bp=-1.3, k_fast=1, k_slow=0.01	71.9	3.04e+03	67.4	3.52e+03
Qian2011-SF23-00	I=60	O	diagonal-crossing-time:10800:100	condensed, dG_bp=-1.3, k_fast=1, k_slow=0.01	53.8	4.99e+03	39.1	6.59e+03
Qian2011-SF23-00	I=50	O	diagonal-crossing-time:10800:100	condensed, dG_bp=-1.3, k_fast=1, k_slow=0.01	3.81	1.04e+04	9.05	9.83e+03
Qian2011-SF23-00	I=40	O	diagonal-crossing-time:10800:100	condensed, dG_bp=-1.3, k_fast=1, k_slow=0.01	2.09	1.06e+04	6.21	1.01e+04
Qian2011-SF23-00	I=30	O	diagonal-crossing-time:10800:100	condensed, dG_bp=-1.3, k_fast=1, k_slow=0.01	1.27	1.07e+04	4.41	1.03e+04
Qian2011-SF23-00	I=20	O	diagonal-crossing-time:10800:100	condensed, dG_bp=-1.3, k_fast=1, k_slow=0.01	0.733	1.07e+04	2.47	1.05e+04
Qian2011-SF23-00	I=10	O	diagonal-crossing-time:10800:100	condensed, dG_bp=-1.3, k_fast=1, k_slow=0.01	0.327	1.08e+04	1.31	1.06e+04
Qian2011-F2C-OR-00	x1=90 x2=90	y	diagonal-crossing-time:18000:100	condensed, k_fast=1, k_slow=0.01	98.3	310	85	3.24e+03
Qian2011-F2C-OR-00	x1=10 x2=90	y	diagonal-crossing-time:18000:100	condensed, k_fast=1, k_slow=0.01	96.9	564	68	4.73e+03
Qian2011-F2C-OR-00	x1=90 x2=10	y	diagonal-crossing-time:18000:100	condensed, k_fast=1, k_slow=0.01	96.9	564	68	4.73e+03
Qian2011-F2C-OR-00	x1=10 x2=10	y	diagonal-crossing-time:18000:100	condensed, k_fast=1, k_slow=0.01	5.85	1.69e+04	0.4	1.05e+04
Qian2011-F2C-OR-00	x1=90 x2=90	y	diagonal-crossing-time:18000:100	condensed, dG_bp=-1.3, k_fast=1, k_slow=0.01	89.1	1.96e+03	85	3.24e+03
Qian2011-F2C-OR-00	x1=10 x2=90	y	diagonal-crossing-time:18000:100	condensed, dG_bp=-1.3, k_fast=1, k_slow=0.01	82.4	3.17e+03	68	4.73e+03
Qian2011-F2C-OR-00	x1=90 x2=10	y	diagonal-crossing-time:18000:100	condensed, dG_bp=-1.3, k_fast=1, k_slow=0.01	82.4	3.17e+03	68	4.73e+03
Qian2011-F2C-OR-00	x1=10 x2=10	y	diagonal-crossing-time:18000:100	condensed, dG_bp=-1.3, k_fast=1, k_slow=0.01	0.587	1.79e+04	0.4	1.05e+04
Qian2011-F2C-AND-00	x1=90 x2=90 T2 5=132	y	diagonal-crossing-time:43200:100	condensed, k_fast=1, k_slow=0.01	98.1	827	68	1.38e+04
Qian2011-F2C-AND-00	x1=10 x2=90 T2 5=132	y	diagonal-crossing-time:43200:100	condensed, k_fast=1, k_slow=0.01	20.9	3.42e+04	0.7	4.1e+04
Qian2011-F2C-AND-00	x1=90 x2=10 T2 5=132	y	diagonal-crossing-time:43200:100	condensed, k_fast=1, k_slow=0.01	20.9	3.42e+04	0.7	4.1e+04
Qian2011-F2C-AND-00	x1=10 x2=10 T2 5=132	y	diagonal-crossing-time:43200:100	condensed, k_fast=1, k_slow=0.01	2.71	4.2e+04	0.4	4.22e+04
Qian2011-F2C-AND-00	x1=90 x2=90 T2 5=132	y	diagonal-crossing-time:43200:100	condensed, dG_bp=-1.3, k_fast=1, k_slow=0.01	88.3	5.08e+03	68	1.38e+04
Qian2011-F2C-AND-00	x1=10 x2=90 T2 5=132	y	diagonal-crossing-time:43200:100	condensed, dG_bp=-1.3, k_fast=1, k_slow=0.01	2.28	4.22e+04	0.7	4.1e+04
Qian2011-F2C-AND-00	x1=90 x2=10 T2 5=132	y	diagonal-crossing-time:43200:100	condensed, dG_bp=-1.3, k_fast=1, k_slow=0.01	2.28	4.22e+04	0.7	4.1e+04
Qian2011-F2C-AND-00	x1=10 x2=10 T2 5=132	y	diagonal-crossing-time:43200:100	condensed, dG_bp=-1.3, k_fast=1, k_slow=0.01	0.268	4.31e+04	0.4	4.22e+04
Qian2011-SF26-00	((1 ∨ 1) ∨ 1) ∨ 1	y	diagonal-crossing-time:21600:100	condensed, k_fast=1, k_slow=0.01	98.4	354	84.5	3.33e+03
Qian2011-SF26-00	((0 ∨ 0) ∨ 1) ∨ 0	y	diagonal-crossing-time:21600:100	condensed, k_fast=1, k_slow=0.01	96.7	728	65.7	7.37e+03
Qian2011-SF26-00	((0 ∨ 1) ∨ 0) ∨ 0	y	diagonal-crossing-time:21600:100	condensed, k_fast=1, k_slow=0.01	96.2	834	55.5	9.56e+03
Qian2011-SF26-00	((0 ∨ 0) ∨ 0) ∨ 0	y	diagonal-crossing-time:21600:100	condensed, k_fast=1, k_slow=0.01	4.06	2.07e+04	3.67	2.08e+04
Qian2011-SF26-00	((1 ∨ 1) ∨ 1) ∨ 1	y	diagonal-crossing-time:21600:100	condensed, k_fast=1, dG_bp=-1.3, k_slow=0.01	88.7	2.43e+03	84.5	3.33e+03
Qian2011-SF26-00	((0 ∨ 0) ∨ 1) ∨ 0	y	diagonal-crossing-time:21600:100	condensed, k_fast=1, dG_bp=-1.3, k_slow=0.01	75.2	5.36e+03	65.7	7.37e+03
Qian2011-SF26-00	((0 ∨ 1) ∨ 0) ∨ 0	y	diagonal-crossing-time:21600:100	condensed, k_fast=1, dG_bp=-1.3, k_slow=0.01	70.1	6.46e+03	55.5	9.56e+03
Qian2011-SF26-00	((0 ∨ 0) ∨ 0) ∨ 0	y	diagonal-crossing-time:21600:100	condensed, k_fast=1, dG_bp=-1.3, k_slow=0.01	0.276	2.15e+04	3.67	2.08e+04
Qian2011-SF27-00	(((1 ∨ 1) ∨ 1) ∨ 1) ∨ 1	y	diagonal-crossing-time:21600:100	condensed, k_fast=1, k_slow=0.01	98.4	356	82.9	3.56e+03
Qian2011-SF27-00	(((0 ∨ 0) ∨ 1) ∨ 0) ∨ 0	y	diagonal-crossing-time:21600:100	condensed, k_fast=1, k_slow=0.01	96.1	848	53.8	9.85e+03
Qian2011-SF27-00	(((0 ∨ 1) ∨ 0) ∨ 0) ∨ 0	y	diagonal-crossing-time:21600:100	condensed, k_fast=1, k_slow=0.01	95.6	960	43.9	1.21e+04
Qian2011-SF27-00	(((0 ∨ 0) ∨ 0) ∨ 0) ∨ 0	y	diagonal-crossing-time:21600:100	condensed, k_fast=1, k_slow=0.01	3.93	2.08e+04	3.28	2.09e+04
Qian2011-SF27-00	(((1 ∨ 1) ∨ 1) ∨ 1) ∨ 1	y	diagonal-crossing-time:21600:100	condensed, k_fast=1, dG_bp=-1.3, k_slow=0.01	88.5	2.48e+03	82.9	3.56e+03
Qian2011-SF27-00	(((0 ∨ 0) ∨ 1) ∨ 0) ∨ 0	y	diagonal-crossing-time:21600:100	condensed, k_fast=1, dG_bp=-1.3, k_slow=0.01	67.7	6.99e+03	53.8	9.85e+03
Qian2011-SF27-00	(((0 ∨ 1) ∨ 0) ∨ 0) ∨ 0	y	diagonal-crossing-time:21600:100	condensed, k_fast=1, dG_bp=-1.3, k_slow=0.01	62.3	8.15e+03	43.9	1.21e+04

Qian2011-SF27-00	(((0 ∨ 0) ∨ 0) ∨ 0) ∨ 0	y	diagonal-crossing-time:21600:100	condensed, k_fast=1, dG_bp=-1.3, k_slow=0.01	0.276	2.15e+04	3.28	2.09e+04
Qian2011-SF28-00	(((1 ∨ 1) ∧ 1) ∨ 1) ∨ (1 ∧ 1)	y	diagonal-crossing-time:28800:100	condensed, k_fast=1, k_slow=0.01	98.5	444	83.8	4.47e+03
Qian2011-SF28-00	(((1 ∨ 0) ∧ 1) ∨ 1) ∨ (0 ∧ 0)	y	diagonal-crossing-time:28800:100	condensed, k_fast=1, k_slow=0.01	97.3	778	73.5	7.54e+03
Qian2011-SF28-00	(((1 ∨ 1) ∧ 0) ∨ 1) ∨ (0 ∧ 0)	y	diagonal-crossing-time:28800:100	condensed, k_fast=1, k_slow=0.01	97.2	820	71.5	8.22e+03
Qian2011-SF28-00	(((0 ∨ 0) ∧ 0) ∨ 1) ∨ (0 ∧ 1)	y	diagonal-crossing-time:28800:100	condensed, k_fast=1, k_slow=0.01	97.6	682	69.5	8.82e+03
Qian2011-SF28-00	(((0 ∨ 0) ∧ 0) ∨ 0) ∨ (1 ∧ 1)	y	diagonal-crossing-time:28800:100	condensed, k_fast=1, k_slow=0.01	97	882	57.2	1.22e+04
Qian2011-SF28-00	(((1 ∨ 0) ∧ 1) ∨ 0) ∨ (1 ∧ 0)	y	diagonal-crossing-time:28800:100	condensed, k_fast=1, k_slow=0.01	96.9	900	51.3	1.39e+04
Qian2011-SF28-00	(((0 ∨ 1) ∧ 1) ∨ 0) ∨ (0 ∧ 0)	y	diagonal-crossing-time:28800:100	condensed, k_fast=1, k_slow=0.01	96.2	1.1e+03	42.6	1.64e+04
Qian2011-SF28-00	(((1 ∨ 1) ∧ 0) ∨ 0) ∨ (0 ∧ 1)	y	diagonal-crossing-time:28800:100	condensed, k_fast=1, k_slow=0.01	11	2.56e+04	2.57	2.8e+04
Qian2011-SF28-00	(((1 ∨ 0) ∧ 0) ∨ 0) ∨ (1 ∧ 0)	y	diagonal-crossing-time:28800:100	condensed, k_fast=1, k_slow=0.01	11.1	2.56e+04	2.57	2.8e+04
Qian2011-SF28-00	(((0 ∨ 1) ∧ 0) ∨ 0) ∨ (0 ∧ 1)	y	diagonal-crossing-time:28800:100	condensed, k_fast=1, k_slow=0.01	11.1	2.56e+04	2.57	2.8e+04
Qian2011-SF28-00	(((0 ∨ 0) ∧ 1) ∨ 0) ∨ (1 ∧ 0)	y	diagonal-crossing-time:28800:100	condensed, k_fast=1, k_slow=0.01	9.73	2.6e+04	2.57	2.8e+04
Qian2011-SF28-00	(((0 ∨ 0) ∧ 0) ∨ 0) ∨ (0 ∧ 0)	y	diagonal-crossing-time:28800:100	condensed, k_fast=1, k_slow=0.01	1.6	2.83e+04	2.57	2.8e+04
Qian2011-SF28-00	(((1 ∨ 1) ∧ 1) ∨ 1) ∨ (1 ∧ 1)	y	diagonal-crossing-time:28800:100	condensed, k_fast=1, dG_bp=-1.3, k_slow=0.01	87.6	3.57e+03	83.8	4.47e+03
Qian2011-SF28-00	(((1 ∨ 0) ∧ 1) ∨ 1) ∨ (0 ∧ 0)	y	diagonal-crossing-time:28800:100	condensed, k_fast=1, dG_bp=-1.3, k_slow=0.01	79.6	5.87e+03	73.5	7.54e+03
Qian2011-SF28-00	(((1 ∨ 1) ∧ 0) ∨ 1) ∨ (0 ∧ 0)	y	diagonal-crossing-time:28800:100	condensed, k_fast=1, dG_bp=-1.3, k_slow=0.01	77.4	6.5e+03	71.5	8.22e+03
Qian2011-SF28-00	(((0 ∨ 0) ∧ 0) ∨ 1) ∨ (0 ∧ 1)	y	diagonal-crossing-time:28800:100	condensed, k_fast=1, dG_bp=-1.3, k_slow=0.01	77.6	6.46e+03	69.5	8.82e+03
Qian2011-SF28-00	(((0 ∨ 0) ∧ 0) ∨ 0) ∨ (1 ∧ 1)	y	diagonal-crossing-time:28800:100	condensed, k_fast=1, dG_bp=-1.3, k_slow=0.01	76.8	6.68e+03	57.2	1.22e+04
Qian2011-SF28-00	(((1 ∨ 0) ∧ 1) ∨ 0) ∨ (1 ∧ 0)	y	diagonal-crossing-time:28800:100	condensed, k_fast=1, dG_bp=-1.3, k_slow=0.01	67.2	9.46e+03	51.3	1.39e+04
Qian2011-SF28-00	(((0 ∨ 1) ∧ 1) ∨ 0) ∨ (0 ∧ 0)	y	diagonal-crossing-time:28800:100	condensed, k_fast=1, dG_bp=-1.3, k_slow=0.01	66.6	9.63e+03	42.6	1.64e+04
Qian2011-SF28-00	(((1 ∨ 1) ∧ 0) ∨ 0) ∨ (0 ∧ 1)	y	diagonal-crossing-time:28800:100	condensed, k_fast=1, dG_bp=-1.3, k_slow=0.01	0.0664	2.88e+04	2.57	2.8e+04
Qian2011-SF28-00	(((1 ∨ 0) ∧ 0) ∨ 0) ∨ (1 ∧ 0)	y	diagonal-crossing-time:28800:100	condensed, k_fast=1, dG_bp=-1.3, k_slow=0.01	0.0664	2.88e+04	2.57	2.8e+04
Qian2011-SF28-00	(((0 ∨ 1) ∧ 0) ∨ 0) ∨ (0 ∧ 1)	y	diagonal-crossing-time:28800:100	condensed, k_fast=1, dG_bp=-1.3, k_slow=0.01	0.0664	2.88e+04	2.57	2.8e+04
Qian2011-SF28-00	(((0 ∨ 0) ∧ 1) ∨ 0) ∨ (1 ∧ 0)	y	diagonal-crossing-time:28800:100	condensed, k_fast=1, dG_bp=-1.3, k_slow=0.01	0.0656	2.88e+04	2.57	2.8e+04
Qian2011-SF28-00	(((0 ∨ 0) ∧ 0) ∨ 0) ∨ (0 ∧ 0)	y	diagonal-crossing-time:28800:100	condensed, k_fast=1, dG_bp=-1.3, k_slow=0.01	0.0134	2.88e+04	2.57	2.8e+04
Qian2011-SF29-OR-00	(1 ∨ 0) ∨ (1 ∨ 0) ∨ (1 ∨ 0) ∨ (1 ∨ 0)	y	diagonal-crossing-time:10800:100	condensed, k_fast=1, k_slow=0.01	98	222	81.2	1.93e+03
Qian2011-SF29-OR-00	(1 ∨ 0) ∨ (1 ∨ 0) ∨ (1 ∨ 0) ∨ (0 ∨ 0)	y	diagonal-crossing-time:10800:100	condensed, k_fast=1, k_slow=0.01	97.8	248	78.4	2.3e+03
Qian2011-SF29-OR-00	(1 ∨ 0) ∨ (1 ∨ 0) ∨ (0 ∨ 0) ∨ (0 ∨ 0)	y	diagonal-crossing-time:10800:100	condensed, k_fast=1, k_slow=0.01	97.3	300	74.8	2.68e+03
Qian2011-SF29-OR-00	(0 ∨ 0) ∨ (0 ∨ 0) ∨ (0 ∨ 0) ∨ (1 ∨ 0)	y	diagonal-crossing-time:10800:100	condensed, k_fast=1, k_slow=0.01	95.5	486	63.6	3.81e+03
Qian2011-SF29-OR-00	(0 ∨ 0) ∨ (0 ∨ 0) ∨ (1 ∨ 0) ∨ (0 ∨ 0)	y	diagonal-crossing-time:10800:100	condensed, k_fast=1, k_slow=0.01	95.5	486	61.2	4.1e+03
Qian2011-SF29-OR-00	(0 ∨ 0) ∨ (1 ∨ 0) ∨ (0 ∨ 0) ∨ (0 ∨ 0)	y	diagonal-crossing-time:10800:100	condensed, k_fast=1, k_slow=0.01	95.5	486	59.2	4.31e+03
Qian2011-SF29-OR-00	(1 ∨ 0) ∨ (0 ∨ 0) ∨ (0 ∨ 0) ∨ (0 ∨ 0)	y	diagonal-crossing-time:10800:100	condensed, k_fast=1, k_slow=0.01	95.5	486	58	4.48e+03
Qian2011-SF29-OR-00	(0 ∨ 0) ∨ (0 ∨ 0) ∨ (0 ∨ 0) ∨ (0 ∨ 0)	y	diagonal-crossing-time:10800:100	condensed, k_fast=1, k_slow=0.01	7.07	1e+04	3.6	1.05e+04
Qian2011-SF29-OR-00	(1 ∨ 0) ∨ (1 ∨ 0) ∨ (1 ∨ 0) ∨ (1 ∨ 0)	y	diagonal-crossing-time:10800:100	condensed, k_fast=1, dG_bp=-1.3, k_slow=0.01	83.8	1.76e+03	81.2	1.93e+03
Qian2011-SF29-OR-00	(1 ∨ 0) ∨ (1 ∨ 0) ∨ (1 ∨ 0) ∨ (0 ∨ 0)	y	diagonal-crossing-time:10800:100	condensed, k_fast=1, dG_bp=-1.3, k_slow=0.01	81.9	1.96e+03	78.4	2.3e+03
Qian2011-SF29-OR-00	(1 ∨ 0) ∨ (1 ∨ 0) ∨ (0 ∨ 0) ∨ (0 ∨ 0)	y	diagonal-crossing-time:10800:100	condensed, k_fast=1, dG_bp=-1.3, k_slow=0.01	77.9	2.39e+03	74.8	2.68e+03
Qian2011-SF29-OR-00	(0 ∨ 0) ∨ (0 ∨ 0) ∨ (0 ∨ 0) ∨ (1 ∨ 0)	y	diagonal-crossing-time:10800:100	condensed, k_fast=1, dG_bp=-1.3, k_slow=0.01	62.3	4.07e+03	63.6	3.81e+03
Qian2011-SF29-OR-00	(0 ∨ 0) ∨ (0 ∨ 0) ∨ (1 ∨ 0) ∨ (0 ∨ 0)	y	diagonal-crossing-time:10800:100	condensed, k_fast=1, dG_bp=-1.3, k_slow=0.01	62.3	4.07e+03	61.2	4.1e+03
Qian2011-SF29-OR-00	(0 ∨ 0) ∨ (1 ∨ 0) ∨ (0 ∨ 0) ∨ (0 ∨ 0)	y	diagonal-crossing-time:10800:100	condensed, k_fast=1, dG_bp=-1.3, k_slow=0.01	62.3	4.07e+03	59.2	4.31e+03
Qian2011-SF29-OR-00	(1 ∨ 0) ∨ (0 ∨ 0) ∨ (0 ∨ 0) ∨ (0 ∨ 0)	y	diagonal-crossing-time:10800:100	condensed, k_fast=1, dG_bp=-1.3, k_slow=0.01	62.3	4.07e+03	58	4.48e+03
Qian2011-SF29-OR-00	(0 ∨ 0) ∨ (0 ∨ 0) ∨ (0 ∨ 0) ∨ (0 ∨ 0)	y	diagonal-crossing-time:10800:100	condensed, k_fast=1, dG_bp=-1.3, k_slow=0.01	0.0591	1.08e+04	3.6	1.05e+04
Qian2011-SF29-AND-00	(1 ∨ 0) ∧ (1 ∨ 0) ∧ (1 ∨ 0) ∧ (1 ∨ 0)	y	diagonal-crossing-time:36000:100	condensed, k_fast=1, k_slow=0.01	94.3	2.04e+03	55.3	1.59e+04
Qian2011-SF29-AND-00	(1 ∨ 0) ∧ (1 ∨ 0) ∧ (1 ∨ 0) ∧ (0 ∨ 0)	y	diagonal-crossing-time:36000:100	condensed, k_fast=1, k_slow=0.01	28.8	2.56e+04	11.1	3.19e+04
Qian2011-SF29-AND-00	(1 ∨ 0) ∧ (1 ∨ 0) ∧ (0 ∨ 0) ∧ (0 ∨ 0)	y	diagonal-crossing-time:36000:100	condensed, k_fast=1, k_slow=0.01	6.59	3.36e+04	5.79	3.43e+04
Qian2011-SF29-AND-00	(1 ∨ 0) ∧ (1 ∨ 0) ∧ (0 ∨ 0) ∧ (0 ∨ 0)	y	diagonal-crossing-time:36000:100	condensed, k_fast=1, k_slow=0.01	6.59	3.36e+04	2.94	3.51e+04
Qian2011-SF29-AND-00	(1 ∨ 0) ∧ (1 ∨ 0) ∧ (1 ∨ 0) ∧ (1 ∨ 0)	y	diagonal-crossing-time:36000:100	condensed, k_fast=1, dG_bp=-1.3, k_slow=0.01	66.7	1.2e+04	55.3	1.59e+04
Qian2011-SF29-AND-00	(1 ∨ 0) ∧ (1 ∨ 0) ∧ (1 ∨ 0) ∧ (0 ∨ 0)	y	diagonal-crossing-time:36000:100	condensed, k_fast=1, dG_bp=-1.3, k_slow=0.01	3.09	3.49e+04	11.1	3.19e+04
Qian2011-SF29-AND-00	(1 ∨ 0) ∧ (1 ∨ 0) ∧ (0 ∨ 0) ∧ (0 ∨ 0)	y	diagonal-crossing-time:36000:100	condensed, k_fast=1, dG_bp=-1.3, k_slow=0.01	0.555	3.58e+04	5.79	3.43e+04
Qian2011-SF29-AND-00	(1 ∨ 0) ∧ (1 ∨ 0) ∧ (0 ∨ 0) ∧ (0 ∨ 0)	y	diagonal-crossing-time:36000:100	condensed, k_fast=1, dG_bp=-1.3, k_slow=0.01	0.555	3.58e+04	2.94	3.51e+04
Qian2011-SF31-00	0000	y1_0	diagonal-crossing-time:36000:50	condensed, seesaw-conc=5e-08	47.7	1.69e+03	21	1.51e+04
Qian2011-SF31-00	0001	y1_0	diagonal-crossing-time:36000:50	condensed, seesaw-conc=5e-08	15.8	2.47e+04	5	2.56e+04
Qian2011-SF31-00	0010	y1_0	diagonal-crossing-time:36000:50	condensed, seesaw-conc=5e-08	15.8	2.47e+04	5	2.56e+04
Qian2011-SF31-00	0011	y1_0	diagonal-crossing-time:36000:50	condensed, seesaw-conc=5e-08	12.6	2.69e+04	5	2.56e+04
Qian2011-SF31-00	0100	y1_0	diagonal-crossing-time:36000:50	condensed, seesaw-conc=5e-08	47.7	1.66e+03	27.5	1.4e+04
Qian2011-SF31-00	0101	y1_0	diagonal-crossing-time:36000:50	condensed, seesaw-conc=5e-08	47.5	1.77e+03	22.5	1.48e+04
Qian2011-SF31-00	0110	y1_0	diagonal-crossing-time:36000:50	condensed, seesaw-conc=5e-08	47.5	1.77e+03	22.5	1.48e+04
Qian2011-SF31-00	0111	y1_0	diagonal-crossing-time:36000:50	condensed, seesaw-conc=5e-08	47.3	1.94e+03	22.5	1.48e+04
Qian2011-SF31-00	1000	y1_0	diagonal-crossing-time:36000:50	condensed, seesaw-conc=5e-08	47.4	1.91e+03	21	1.62e+04
Qian2011-SF31-00	1001	y1_0	diagonal-crossing-time:36000:50	condensed, seesaw-conc=5e-08	12.2	2.72e+04	5	2.63e+04
Qian2011-SF31-00	1010	y1_0	diagonal-crossing-time:36000:50	condensed, seesaw-conc=5e-08	12.2	2.72e+04	5	2.63e+04
Qian2011-SF31-00	1011	y1_0	diagonal-crossing-time:36000:50	condensed, seesaw-conc=5e-08	10.8	2.82e+04	5	2.63e+04
Qian2011-SF31-00	1100	y1_0	diagonal-crossing-time:36000:50	condensed, seesaw-conc=5e-08	12.3	2.71e+04	5	2.63e+04
Qian2011-SF31-00	1101	y1_0	diagonal-crossing-time:36000:50	condensed, seesaw-conc=5e-08	15.8	2.46e+04	5	2.63e+04
Qian2011-SF31-00	1110	y1_0	diagonal-crossing-time:36000:50	condensed, seesaw-conc=5e-08	15.8	2.46e+04	5	2.63e+04
Qian2011-SF31-00	1111	y1_0	diagonal-crossing-time:36000:50	condensed, seesaw-conc=5e-08	12.7	2.69e+04	5	2.63e+04
Qian2011-SF31-00	0000	y1_1	diagonal-crossing-time:36000:50	condensed, seesaw-conc=5e-08	5.83	3.18e+04	5	2.56e+04
Qian2011-SF31-00	0001	y1_1	diagonal-crossing-time:36000:50	condensed, seesaw-conc=5e-08	47.2	1.98e+03	20	1.8e+04
Qian2011-SF31-00	0010	y1_1	diagonal-crossing-time:36000:50	condensed, seesaw-conc=5e-08	47.2	1.98e+03	20	1.8e+04
Qian2011-SF31-00	0011	y1_1	diagonal-crossing-time:36000:50	condensed, seesaw-conc=5e-08	47.3	1.94e+03	21	1.51e+04
Qian2011-SF31-00	0100	y1_1	diagonal-crossing-time:36000:50	condensed, seesaw-conc=5e-08	4.84	3.25e+04	4	2.63e+04
Qian2011-SF31-00	0101	y1_1	diagonal-crossing-time:36000:50	condensed, seesaw-conc=5e-08	6.13	3.16e+04	4	2.63e+04
Qian2011-SF31-00	0110	y1_1	diagonal-crossing-time:36000:50	condensed, seesaw-conc=5e-08	6.13	3.16e+04	4	2.63e+04
Qian2011-SF31-00	0111	y1_1	diagonal-crossing-time:36000:50	condensed, seesaw-conc=5e-08	5.58	3.2e+04	4	2.63e+04
Qian2011-SF31-00	1000	y1_1	diagonal-crossing-time:36000:50	condensed, seesaw-conc=5e-08	11.5	2.77e+04	5	2.48e+04

Qian2011-SF31-00	1001	y1_1	diagonal-crossing-time:36000:50	condensed, seesaw-conc=5e-08	47.7	1.66e+03	25	1.44e+04
Qian2011-SF31-00	1010	y1_1	diagonal-crossing-time:36000:50	condensed, seesaw-conc=5e-08	47.7	1.66e+03	25	1.44e+04
Qian2011-SF31-00	1011	y1_1	diagonal-crossing-time:36000:50	condensed, seesaw-conc=5e-08	47.8	1.62e+03	30	1.37e+04
Qian2011-SF31-00	1100	y1_1	diagonal-crossing-time:36000:50	condensed, seesaw-conc=5e-08	47.6	1.75e+03	12.5	2.16e+04
Qian2011-SF31-00	1101	y1_1	diagonal-crossing-time:36000:50	condensed, seesaw-conc=5e-08	48	1.46e+03	25.5	1.44e+04
Qian2011-SF31-00	1110	y1_1	diagonal-crossing-time:36000:50	condensed, seesaw-conc=5e-08	48	1.46e+03	25.5	1.44e+04
Qian2011-SF31-00	1111	y1_1	diagonal-crossing-time:36000:50	condensed, seesaw-conc=5e-08	48	1.46e+03	30	1.26e+04
Qian2011-SF31-00	0000	y2_0	diagonal-crossing-time:36000:50	condensed, seesaw-conc=5e-08	48	1.46e+03	27.5	1.12e+04
Qian2011-SF31-00	0001	y2_0	diagonal-crossing-time:36000:50	condensed, seesaw-conc=5e-08	48	1.45e+03	30	1.08e+04
Qian2011-SF31-00	0010	y2_0	diagonal-crossing-time:36000:50	condensed, seesaw-conc=5e-08	48	1.45e+03	30	1.08e+04
Qian2011-SF31-00	0011	y2_0	diagonal-crossing-time:36000:50	condensed, seesaw-conc=5e-08	48	1.45e+03	30	1.08e+04
Qian2011-SF31-00	0100	y2_0	diagonal-crossing-time:36000:50	condensed, seesaw-conc=5e-08	12.8	2.68e+04	5	2.45e+04
Qian2011-SF31-00	0101	y2_0	diagonal-crossing-time:36000:50	condensed, seesaw-conc=5e-08	12.8	2.68e+04	5	2.45e+04
Qian2011-SF31-00	0110	y2_0	diagonal-crossing-time:36000:50	condensed, seesaw-conc=5e-08	12.8	2.68e+04	5	2.45e+04
Qian2011-SF31-00	0111	y2_0	diagonal-crossing-time:36000:50	condensed, seesaw-conc=5e-08	12.8	2.68e+04	5	2.45e+04
Qian2011-SF31-00	1000	y2_0	diagonal-crossing-time:36000:50	condensed, seesaw-conc=5e-08	12.8	2.68e+04	5.5	2.52e+04
Qian2011-SF31-00	1001	y2_0	diagonal-crossing-time:36000:50	condensed, seesaw-conc=5e-08	12.8	2.68e+04	5.5	2.52e+04
Qian2011-SF31-00	1010	y2_0	diagonal-crossing-time:36000:50	condensed, seesaw-conc=5e-08	12.8	2.68e+04	5.5	2.52e+04
Qian2011-SF31-00	1011	y2_0	diagonal-crossing-time:36000:50	condensed, seesaw-conc=5e-08	12.8	2.68e+04	5.5	2.52e+04
Qian2011-SF31-00	1100	y2_0	diagonal-crossing-time:36000:50	condensed, seesaw-conc=5e-08	1.3	3.51e+04	4	2.59e+04
Qian2011-SF31-00	1101	y2_0	diagonal-crossing-time:36000:50	condensed, seesaw-conc=5e-08	1.3	3.51e+04	4	2.59e+04
Qian2011-SF31-00	1110	y2_0	diagonal-crossing-time:36000:50	condensed, seesaw-conc=5e-08	1.3	3.51e+04	4	2.59e+04
Qian2011-SF31-00	1111	y2_0	diagonal-crossing-time:36000:50	condensed, seesaw-conc=5e-08	1.3	3.51e+04	4	2.59e+04
Qian2011-SF31-00	0000	y2_1	diagonal-crossing-time:36000:50	condensed, seesaw-conc=5e-08	2.8	3.4e+04	5	2.52e+04
Qian2011-SF31-00	0001	y2_1	diagonal-crossing-time:36000:50	condensed, seesaw-conc=5e-08	2.8	3.4e+04	5	2.52e+04
Qian2011-SF31-00	0010	y2_1	diagonal-crossing-time:36000:50	condensed, seesaw-conc=5e-08	2.8	3.4e+04	5	2.52e+04
Qian2011-SF31-00	0011	y2_1	diagonal-crossing-time:36000:50	condensed, seesaw-conc=5e-08	2.8	3.4e+04	5	2.52e+04
Qian2011-SF31-00	0100	y2_1	diagonal-crossing-time:36000:50	condensed, seesaw-conc=5e-08	48.4	1.19e+03	37.5	7.2e+03
Qian2011-SF31-00	0101	y2_1	diagonal-crossing-time:36000:50	condensed, seesaw-conc=5e-08	48.4	1.19e+03	37.5	7.2e+03
Qian2011-SF31-00	0110	y2_1	diagonal-crossing-time:36000:50	condensed, seesaw-conc=5e-08	48.4	1.19e+03	37.5	7.2e+03
Qian2011-SF31-00	0111	y2_1	diagonal-crossing-time:36000:50	condensed, seesaw-conc=5e-08	48.4	1.19e+03	37.5	7.2e+03
Qian2011-SF31-00	1000	y2_1	diagonal-crossing-time:36000:50	condensed, seesaw-conc=5e-08	48.4	1.18e+03	40	6.48e+03
Qian2011-SF31-00	1001	y2_1	diagonal-crossing-time:36000:50	condensed, seesaw-conc=5e-08	48.4	1.18e+03	40	6.48e+03
Qian2011-SF31-00	1010	y2_1	diagonal-crossing-time:36000:50	condensed, seesaw-conc=5e-08	48.4	1.18e+03	40	6.48e+03
Qian2011-SF31-00	1011	y2_1	diagonal-crossing-time:36000:50	condensed, seesaw-conc=5e-08	48.4	1.18e+03	40	6.48e+03
Qian2011-SF31-00	1100	y2_1	diagonal-crossing-time:36000:50	condensed, seesaw-conc=5e-08	49	710	40	7.2e+03
Qian2011-SF31-00	1101	y2_1	diagonal-crossing-time:36000:50	condensed, seesaw-conc=5e-08	49	710	40	7.2e+03
Qian2011-SF31-00	1110	y2_1	diagonal-crossing-time:36000:50	condensed, seesaw-conc=5e-08	49	710	40	7.2e+03
Qian2011-SF31-00	1111	y2_1	diagonal-crossing-time:36000:50	condensed, seesaw-conc=5e-08	49	710	40	7.2e+03
Qian2011-SF31-00	0000	y1_0	diagonal-crossing-time:36000:50	seesaw-conc=5e-08, condensed, dG_bp=-1.3	30.2	1.43e+04	21	1.51e+04
Qian2011-SF31-00	0001	y1_0	diagonal-crossing-time:36000:50	seesaw-conc=5e-08, condensed, dG_bp=-1.3	1.14	3.52e+04	5	2.56e+04
Qian2011-SF31-00	0010	y1_0	diagonal-crossing-time:36000:50	seesaw-conc=5e-08, condensed, dG_bp=-1.3	1.14	3.52e+04	5	2.56e+04
Qian2011-SF31-00	0011	y1_0	diagonal-crossing-time:36000:50	seesaw-conc=5e-08, condensed, dG_bp=-1.3	1.14	3.52e+04	5	2.56e+04
Qian2011-SF31-00	0100	y1_0	diagonal-crossing-time:36000:50	seesaw-conc=5e-08, condensed, dG_bp=-1.3	33.6	1.18e+04	27.5	1.4e+04
Qian2011-SF31-00	0101	y1_0	diagonal-crossing-time:36000:50	seesaw-conc=5e-08, condensed, dG_bp=-1.3	28.6	1.54e+04	22.5	1.48e+04
Qian2011-SF31-00	0110	y1_0	diagonal-crossing-time:36000:50	seesaw-conc=5e-08, condensed, dG_bp=-1.3	28.6	1.54e+04	22.5	1.48e+04
Qian2011-SF31-00	0111	y1_0	diagonal-crossing-time:36000:50	seesaw-conc=5e-08, condensed, dG_bp=-1.3	28.2	1.57e+04	22.5	1.48e+04
Qian2011-SF31-00	1000	y1_0	diagonal-crossing-time:36000:50	seesaw-conc=5e-08, condensed, dG_bp=-1.3	29.2	1.5e+04	21	1.62e+04
Qian2011-SF31-00	1001	y1_0	diagonal-crossing-time:36000:50	seesaw-conc=5e-08, condensed, dG_bp=-1.3	1.14	3.52e+04	5	2.63e+04
Qian2011-SF31-00	1010	y1_0	diagonal-crossing-time:36000:50	seesaw-conc=5e-08, condensed, dG_bp=-1.3	1.14	3.52e+04	5	2.63e+04
Qian2011-SF31-00	1011	y1_0	diagonal-crossing-time:36000:50	seesaw-conc=5e-08, condensed, dG_bp=-1.3	1.14	3.52e+04	5	2.63e+04
Qian2011-SF31-00	1100	y1_0	diagonal-crossing-time:36000:50	seesaw-conc=5e-08, condensed, dG_bp=-1.3	1.14	3.52e+04	5	2.63e+04
Qian2011-SF31-00	1101	y1_0	diagonal-crossing-time:36000:50	seesaw-conc=5e-08, condensed, dG_bp=-1.3	1.14	3.52e+04	5	2.63e+04
Qian2011-SF31-00	1110	y1_0	diagonal-crossing-time:36000:50	seesaw-conc=5e-08, condensed, dG_bp=-1.3	1.14	3.52e+04	5	2.63e+04
Qian2011-SF31-00	1111	y1_0	diagonal-crossing-time:36000:50	seesaw-conc=5e-08, condensed, dG_bp=-1.3	1.14	3.52e+04	5	2.63e+04
Qian2011-SF31-00	0000	y1_1	diagonal-crossing-time:36000:50	seesaw-conc=5e-08, condensed, dG_bp=-1.3	0.0405	3.6e+04	5	2.56e+04
Qian2011-SF31-00	0001	y1_1	diagonal-crossing-time:36000:50	seesaw-conc=5e-08, condensed, dG_bp=-1.3	27	1.66e+04	20	1.8e+04
Qian2011-SF31-00	0010	y1_1	diagonal-crossing-time:36000:50	seesaw-conc=5e-08, condensed, dG_bp=-1.3	27	1.66e+04	20	1.8e+04
Qian2011-SF31-00	0011	y1_1	diagonal-crossing-time:36000:50	seesaw-conc=5e-08, condensed, dG_bp=-1.3	28.2	1.57e+04	21	1.51e+04
Qian2011-SF31-00	0100	y1_1	diagonal-crossing-time:36000:50	seesaw-conc=5e-08, condensed, dG_bp=-1.3	0.0361	3.6e+04	4	2.63e+04
Qian2011-SF31-00	0101	y1_1	diagonal-crossing-time:36000:50	seesaw-conc=5e-08, condensed, dG_bp=-1.3	0.0404	3.6e+04	4	2.63e+04
Qian2011-SF31-00	0110	y1_1	diagonal-crossing-time:36000:50	seesaw-conc=5e-08, condensed, dG_bp=-1.3	0.0404	3.6e+04	4	2.63e+04
Qian2011-SF31-00	0111	y1_1	diagonal-crossing-time:36000:50	seesaw-conc=5e-08, condensed, dG_bp=-1.3	0.0401	3.6e+04	4	2.63e+04
Qian2011-SF31-00	1000	y1_1	diagonal-crossing-time:36000:50	seesaw-conc=5e-08, condensed, dG_bp=-1.3	0.0664	3.6e+04	5	2.48e+04
Qian2011-SF31-00	1001	y1_1	diagonal-crossing-time:36000:50	seesaw-conc=5e-08, condensed, dG_bp=-1.3	29.7	1.46e+04	25	1.44e+04
Qian2011-SF31-00	1010	y1_1	diagonal-crossing-time:36000:50	seesaw-conc=5e-08, condensed, dG_bp=-1.3	29.7	1.46e+04	25	1.44e+04
Qian2011-SF31-00	1011	y1_1	diagonal-crossing-time:36000:50	seesaw-conc=5e-08, condensed, dG_bp=-1.3	31.3	1.35e+04	30	1.37e+04
Qian2011-SF31-00	1100	y1_1	diagonal-crossing-time:36000:50	seesaw-conc=5e-08, condensed, dG_bp=-1.3	23.4	1.91e+04	12.5	2.16e+04
Qian2011-SF31-00	1101	y1_1	diagonal-crossing-time:36000:50	seesaw-conc=5e-08, condensed, dG_bp=-1.3	27.5	1.62e+04	25.5	1.44e+04
Qian2011-SF31-00	1110	y1_1	diagonal-crossing-time:36000:50	seesaw-conc=5e-08, condensed, dG_bp=-1.3	27.5	1.62e+04	25.5	1.44e+04
Qian2011-SF31-00	1111	y1_1	diagonal-crossing-time:36000:50	seesaw-conc=5e-08, condensed, dG_bp=-1.3	28.7	1.53e+04	30	1.26e+04
Qian2011-SF31-00	0000	y2_0	diagonal-crossing-time:36000:50	seesaw-conc=5e-08, condensed, dG_bp=-1.3	38.2	8.49e+03	27.5	1.12e+04
Qian2011-SF31-00	0001	y2_0	diagonal-crossing-time:36000:50	seesaw-conc=5e-08, condensed, dG_bp=-1.3	38.2	8.47e+03	30	1.08e+04
Qian2011-SF31-00	0010	y2_0	diagonal-crossing-time:36000:50	seesaw-conc=5e-08, condensed, dG_bp=-1.3	38.2	8.47e+03	30	1.08e+04

Qian2011-SF31-00	0011	y2_0	diagonal-crossing-time:36000:50	seesaw-conc=5e-08, condensed, dG_bp=-1.3	38.2	8.47e+03	30	1.08e+04
Qian2011-SF31-00	0100	y2_0	diagonal-crossing-time:36000:50	seesaw-conc=5e-08, condensed, dG_bp=-1.3	1.17	3.52e+04	5	2.45e+04
Qian2011-SF31-00	0101	y2_0	diagonal-crossing-time:36000:50	seesaw-conc=5e-08, condensed, dG_bp=-1.3	1.17	3.52e+04	5	2.45e+04
Qian2011-SF31-00	0110	y2_0	diagonal-crossing-time:36000:50	seesaw-conc=5e-08, condensed, dG_bp=-1.3	1.17	3.52e+04	5	2.45e+04
Qian2011-SF31-00	0111	y2_0	diagonal-crossing-time:36000:50	seesaw-conc=5e-08, condensed, dG_bp=-1.3	1.17	3.52e+04	5	2.45e+04
Qian2011-SF31-00	1000	y2_0	diagonal-crossing-time:36000:50	seesaw-conc=5e-08, condensed, dG_bp=-1.3	1.17	3.52e+04	5.5	2.52e+04
Qian2011-SF31-00	1001	y2_0	diagonal-crossing-time:36000:50	seesaw-conc=5e-08, condensed, dG_bp=-1.3	1.17	3.52e+04	5.5	2.52e+04
Qian2011-SF31-00	1010	y2_0	diagonal-crossing-time:36000:50	seesaw-conc=5e-08, condensed, dG_bp=-1.3	1.17	3.52e+04	5.5	2.52e+04
Qian2011-SF31-00	1011	y2_0	diagonal-crossing-time:36000:50	seesaw-conc=5e-08, condensed, dG_bp=-1.3	1.17	3.52e+04	5.5	2.52e+04
Qian2011-SF31-00	1100	y2_0	diagonal-crossing-time:36000:50	seesaw-conc=5e-08, condensed, dG_bp=-1.3	0.0122	3.6e+04	4	2.59e+04
Qian2011-SF31-00	1101	y2_0	diagonal-crossing-time:36000:50	seesaw-conc=5e-08, condensed, dG_bp=-1.3	0.0122	3.6e+04	4	2.59e+04
Qian2011-SF31-00	1110	y2_0	diagonal-crossing-time:36000:50	seesaw-conc=5e-08, condensed, dG_bp=-1.3	0.0122	3.6e+04	4	2.59e+04
Qian2011-SF31-00	1111	y2_0	diagonal-crossing-time:36000:50	seesaw-conc=5e-08, condensed, dG_bp=-1.3	0.0122	3.6e+04	4	2.59e+04
Qian2011-SF31-00	0000	y2_1	diagonal-crossing-time:36000:50	seesaw-conc=5e-08, condensed, dG_bp=-1.3	0.0246	3.6e+04	5	2.52e+04
Qian2011-SF31-00	0001	y2_1	diagonal-crossing-time:36000:50	seesaw-conc=5e-08, condensed, dG_bp=-1.3	0.0246	3.6e+04	5	2.52e+04
Qian2011-SF31-00	0010	y2_1	diagonal-crossing-time:36000:50	seesaw-conc=5e-08, condensed, dG_bp=-1.3	0.0246	3.6e+04	5	2.52e+04
Qian2011-SF31-00	0011	y2_1	diagonal-crossing-time:36000:50	seesaw-conc=5e-08, condensed, dG_bp=-1.3	0.0246	3.6e+04	5	2.52e+04
Qian2011-SF31-00	0100	y2_1	diagonal-crossing-time:36000:50	seesaw-conc=5e-08, condensed, dG_bp=-1.3	39	7.94e+03	37.5	7.2e+03
Qian2011-SF31-00	0101	y2_1	diagonal-crossing-time:36000:50	seesaw-conc=5e-08, condensed, dG_bp=-1.3	39	7.94e+03	37.5	7.2e+03
Qian2011-SF31-00	0110	y2_1	diagonal-crossing-time:36000:50	seesaw-conc=5e-08, condensed, dG_bp=-1.3	39	7.94e+03	37.5	7.2e+03
Qian2011-SF31-00	0111	y2_1	diagonal-crossing-time:36000:50	seesaw-conc=5e-08, condensed, dG_bp=-1.3	39	7.95e+03	37.5	7.2e+03
Qian2011-SF31-00	1000	y2_1	diagonal-crossing-time:36000:50	seesaw-conc=5e-08, condensed, dG_bp=-1.3	39	7.92e+03	40	6.48e+03
Qian2011-SF31-00	1001	y2_1	diagonal-crossing-time:36000:50	seesaw-conc=5e-08, condensed, dG_bp=-1.3	39	7.92e+03	40	6.48e+03
Qian2011-SF31-00	1010	y2_1	diagonal-crossing-time:36000:50	seesaw-conc=5e-08, condensed, dG_bp=-1.3	39	7.92e+03	40	6.48e+03
Qian2011-SF31-00	1011	y2_1	diagonal-crossing-time:36000:50	seesaw-conc=5e-08, condensed, dG_bp=-1.3	39	7.92e+03	40	6.48e+03
Qian2011-SF31-00	1100	y2_1	diagonal-crossing-time:36000:50	seesaw-conc=5e-08, condensed, dG_bp=-1.3	43.5	4.68e+03	40	7.2e+03
Qian2011-SF31-00	1101	y2_1	diagonal-crossing-time:36000:50	seesaw-conc=5e-08, condensed, dG_bp=-1.3	43.5	4.68e+03	40	7.2e+03
Qian2011-SF31-00	1110	y2_1	diagonal-crossing-time:36000:50	seesaw-conc=5e-08, condensed, dG_bp=-1.3	43.5	4.68e+03	40	7.2e+03
Qian2011-SF31-00	1111	y2_1	diagonal-crossing-time:36000:50	seesaw-conc=5e-08, condensed, dG_bp=-1.3	43.5	4.68e+03	40	7.2e+03

Table 6: Data for Fig. 10. Qian & Winfree (2011) – Seesaw systems

Input Filename	Simulation	Reporter	Metric	Metric-values	Semantics	Concentration (simulation)	Time (simulation)	Concentration (experiment)	Time (experiment)
Zhang2007-F1-00	C=10	ROX	diagonal-crossing-time	7200:10	condensed	7.18	2.03e+03	7.44	1.22e+03
Zhang2007-F1-00	C=5	ROX	diagonal-crossing-time	7200:10	condensed	6.76	2.33e+03	6.83	1.66e+03
Zhang2007-F1-00	C=2	ROX	diagonal-crossing-time	7200:10	condensed	5.19	3.47e+03	5.78	2.46e+03
Zhang2007-F1-00	C=1	ROX	diagonal-crossing-time	7200:10	condensed	3.74	4.51e+03	4.49	3.44e+03
Zhang2007-F1-00	C=0.5	ROX	diagonal-crossing-time	7200:10	condensed	2.43	5.45e+03	3.26	4.36e+03
Zhang2007-F1-00	C=0.2	ROX	diagonal-crossing-time	7200:10	condensed	1.2	6.33e+03	1.88	5.42e+03
Zhang2007-F1-00	C=0.1	ROX	diagonal-crossing-time	7200:10	condensed	0.655	6.73e+03	1.03	6.07e+03
Zhang2007-F1-00	C=0.05	ROX	diagonal-crossing-time	7200:10	condensed	0.343	6.95e+03	0.57	6.39e+03
Zhang2007-F1-00	C=0.02	ROX	diagonal-crossing-time	7200:10	condensed	0.141	7.1e+03	0.32	6.55e+03
Zhang2007-F1-00	C=10	ROX	completion-time	5	condensed	5	1e+03	5	551
Zhang2007-F1-00	C=5	ROX	completion-time	5	condensed	5	1.47e+03	5	953
Zhang2007-F1-00	C=2	ROX	completion-time	5	condensed	5	3.31e+03	5	1.91e+03
Zhang2007-F1-00	C=1	ROX	completion-time	5	condensed	5	6.44e+03	5	4.08e+03
Zhang2007-F3-00	C0=10	ROX	diagonal-crossing-time	7200:10	condensed	6.54	2.49e+03	6.56	1.86e+03
Zhang2007-F3-00	C0=5	ROX	diagonal-crossing-time	7200:10	condensed	6.31	2.66e+03	5.98	2.3e+03
Zhang2007-F3-00	C0=2	ROX	diagonal-crossing-time	7200:10	condensed	5.56	3.2e+03	5.4	2.74e+03
Zhang2007-F3-00	C0=1	ROX	diagonal-crossing-time	7200:10	condensed	4.73	3.8e+03	4.98	3.07e+03
Zhang2007-F3-00	C0=0.5	ROX	diagonal-crossing-time	7200:10	condensed	3.76	4.49e+03	3.92	3.86e+03
Zhang2007-F3-00	C0=0.2	ROX	diagonal-crossing-time	7200:10	condensed	2.47	5.42e+03	3.07	4.52e+03
Zhang2007-F3-00	C0=0.1	ROX	diagonal-crossing-time	7200:10	condensed	1.63	6.03e+03	2.54	4.91e+03
Zhang2007-F3-00	C0=0.05	ROX	diagonal-crossing-time	7200:10	condensed	0.982	6.49e+03	1.63	5.61e+03
Zhang2007-F3-00	C0=0.02	ROX	diagonal-crossing-time	7200:10	condensed	0.454	6.87e+03	1.23	5.9e+03
Zhang2007-F3-00	C0=0.01	ROX	diagonal-crossing-time	7200:10	condensed	0.24	7.03e+03	1.02	6.07e+03
Zhang2007-F3-00	C0=10	ROX	completion-time	5	condensed	5	1.84e+03	5	1.26e+03
Zhang2007-F3-00	C0=5	ROX	completion-time	5	condensed	5	2.12e+03	5	1.85e+03
Zhang2007-F3-00	C0=2	ROX	completion-time	5	condensed	5	2.95e+03	5	2.51e+03
Zhang2007-F3-00	C0=1	ROX	completion-time	5	condensed	5	3.95e+03	5	3.07e+03
Zhang2007-F3-00	C0=0.5	ROX	completion-time	5	condensed	5	5.39e+03	5	4.85e+03
Zhang2007-F4-00	A=10	TET	diagonal-crossing-time	3600:10	condensed	9.05	341	7.62	496
Zhang2007-F4-00	A=7	TET	diagonal-crossing-time	3600:10	condensed	8.67	481	7.32	601
Zhang2007-F4-00	A=5	TET	diagonal-crossing-time	3600:10	condensed	8.21	646	7.16	655
Zhang2007-F4-00	A=3	TET	diagonal-crossing-time	3600:10	condensed	7.42	930	6.61	856
Zhang2007-F4-00	A=2	TET	diagonal-crossing-time	3600:10	condensed	6.75	1.17e+03	6.45	910
Zhang2007-F4-00	A=1	TET	diagonal-crossing-time	3600:10	condensed	5.6	1.59e+03	6.04	1.06e+03
Zhang2007-F4-00	A=0.7	TET	diagonal-crossing-time	3600:10	condensed	5.01	1.8e+03	5.64	1.21e+03
Zhang2007-F4-00	A=0.5	TET	diagonal-crossing-time	3600:10	condensed	4.48	1.99e+03	5.54	1.24e+03
Zhang2007-F4-00	A=0.3	TET	diagonal-crossing-time	3600:10	condensed	3.7	2.27e+03	5.26	1.33e+03
Zhang2007-F4-00	A=0.2	TET	diagonal-crossing-time	3600:10	condensed	3.12	2.48e+03	5.1	1.39e+03
Zhang2007-F4-00	A=0.1	TET	diagonal-crossing-time	3600:10	condensed	2.23	2.8e+03	4.91	1.47e+03
Zhang2007-F4-00	A=10	TET	completion-time	5	condensed	5	94	5	241
Zhang2007-F4-00	A=7	TET	completion-time	5	condensed	5	138	5	350
Zhang2007-F4-00	A=5	TET	completion-time	5	condensed	5	227	5	394
Zhang2007-F4-00	A=3	TET	completion-time	5	condensed	5	520	5	610
Zhang2007-F4-00	A=2	TET	completion-time	5	condensed	5	831	5	694
Zhang2007-F4-00	A=1	TET	completion-time	5	condensed	5	1.45e+03	5	892
Zhang2007-F4-00	A=0.7	TET	completion-time	5	condensed	5	1.79e+03	5	1.09e+03
Zhang2007-F4-00	A=0.5	TET	completion-time	5	condensed	5	2.13e+03	5	1.12e+03
Zhang2007-F4-00	A=0.3	TET	completion-time	5	condensed	5	2.65e+03	5	1.28e+03
Zhang2007-F4-00	A=0.2	TET	completion-time	5	condensed	5	3.07e+03	5	1.38e+03
Zhang2007-F4-00	A=0.1	TET	completion-time	5	condensed	5	3.8e+03	5	1.47e+03
Yin2008-F3-00	I=20	A	diagonal-crossing-time	18000:2;-20	condensed, k_slow=1e-05, k_fast=0.1	2.08	89	3.3	297
Yin2008-F3-00	I=6	A	diagonal-crossing-time	18000:2;-20	condensed, k_slow=1e-05, k_fast=0.1	2.19	199	5.23	2.26e+03
Yin2008-F3-00	I=2	A	diagonal-crossing-time	18000:2;-20	condensed, k_slow=1e-05, k_fast=0.1	2.29	297	7.37	4.41e+03
Yin2008-F3-00	I=1	A	diagonal-crossing-time	18000:2;-20	condensed, k_slow=1e-05, k_fast=0.1	2.35	353	8.72	5.81e+03
Yin2008-F3-00	I=0.6	A	diagonal-crossing-time	18000:2;-20	condensed, k_slow=1e-05, k_fast=0.1	2.36	393	9.83	6.93e+03
Yin2008-F3-00	I=0.4	A	diagonal-crossing-time	18000:2;-20	condensed, k_slow=1e-05, k_fast=0.1	2.4	423	10.4	7.55e+03
Yin2008-F3-00	I=0.2	A	diagonal-crossing-time	18000:2;-20	condensed, k_slow=1e-05, k_fast=0.1	2.47	473	11.2	8.3e+03
Yin2008-F3-00	I=0.1	A	diagonal-crossing-time	18000:2;-20	condensed, k_slow=1e-05, k_fast=0.1	2.49	523	11.8	8.97e+03
Yin2008-F3-00	I=0.06	A	diagonal-crossing-time	18000:2;-20	condensed, k_slow=1e-05, k_fast=0.1	2.53	559	12.1	9.28e+03
Yin2008-F3-00	I=0.02	A	diagonal-crossing-time	18000:2;-20	condensed, k_slow=1e-05, k_fast=0.1	2.6	636	12.4	9.56e+03
Yin2008-F3-00	I=0.01	A	diagonal-crossing-time	18000:2;-20	condensed, k_slow=1e-05, k_fast=0.1	2.66	684	12.5	9.7e+03
Yin2008-F3-00	I=20	A	completion-time	-10	condensed, k_slow=1e-05, k_fast=0.1	-10	28	-10	45.5
Yin2008-F3-00	I=6	A	completion-time	-10	condensed, k_slow=1e-05, k_fast=0.1	-10	96	-10	1.07e+03
Yin2008-F3-00	I=2	A	completion-time	-10	condensed, k_slow=1e-05, k_fast=0.1	-10	183	-10	3.49e+03
Yin2008-F3-00	I=1	A	completion-time	-10	condensed, k_slow=1e-05, k_fast=0.1	-10	236	-10	5.32e+03
Yin2008-F3-00	I=0.6	A	completion-time	-10	condensed, k_slow=1e-05, k_fast=0.1	-10	275	-10	6.87e+03
Yin2008-F3-00	I=0.4	A	completion-time	-10	condensed, k_slow=1e-05, k_fast=0.1	-10	305	-10	7.7e+03
Yin2008-F3-00	I=0.2	A	completion-time	-10	condensed, k_slow=1e-05, k_fast=0.1	-10	356	-10	8.85e+03
Yin2008-F3-00	I=0.1	A	completion-time	-10	condensed, k_slow=1e-05, k_fast=0.1	-10	406	-10	9.73e+03
Yin2008-F3-00	I=0.06	A	completion-time	-10	condensed, k_slow=1e-05, k_fast=0.1	-10	443	-10	1.02e+04
Yin2008-F3-00	I=0.02	A	completion-time	-10	condensed, k_slow=1e-05, k_fast=0.1	-10	521	-10	1.07e+04
Yin2008-F3-00	I=0.01	A	completion-time	-10	condensed, k_slow=1e-05, k_fast=0.1	-10	571	-10	1.09e+04

Table 7: Data for Fig. 11 Part 1/2.

Input Filename	Simulation	Reporter	Metric	Metric-values	Semantics	Concentration (simulation)	Time (simulation)	Concentration (experiment)	Time (experiment)
Zhang2009-F3-00	X=0.6	F	diagonal-crossing-time	7200:0.4	condensed	0.313	1.58e+03	0.31	1.26e+03
Zhang2009-F3-00	X=0.4	F	diagonal-crossing-time	7200:0.4	condensed	0.272	2.3e+03	0.27	1.87e+03
Zhang2009-F3-00	X=0.2	F	diagonal-crossing-time	7200:0.4	condensed	0.178	4.01e+03	0.17	3.5e+03
Zhang2009-F3-00	X=0.6	F	completion-time	0.3	condensed	0.3	1.48e+03	0.3	1.24e+03
Zhang2009-F3-00	X=0.4	F	completion-time	0.3	condensed	0.3	2.82e+03	0.3	2.43e+03
Zhang2009-F4-00	X=0.4	F	completion-time	0.2	condensed	0.2	564	0.2	528
Zhang2009-F4-01	X=0.4	F	completion-time	0.2	condensed	0.2	566	0.2	578
Zhang2009-F4-02	X=0.4	F	completion-time	0.2	condensed	0.2	590	0.2	983
Zhang2009-F4-03	X=0.4	F	completion-time	0.2	condensed	0.2	949	0.2	1.88e+03
Zhang2009-F4-00	X=0.4	F	diagonal-crossing-time	3600:0.4	condensed	0.292	974	0.28	785
Zhang2009-F4-01	X=0.4	F	diagonal-crossing-time	3600:0.4	condensed	0.292	976	0.27	823
Zhang2009-F4-02	X=0.4	F	diagonal-crossing-time	3600:0.4	condensed	0.289	1e+03	0.23	1.19e+03
Zhang2009-F4-03	X=0.4	F	diagonal-crossing-time	3600:0.4	condensed	0.251	1.34e+03	0.18	1.62e+03
Zhang2009-F5-00	X=0.4	F	diagonal-crossing-time	14400:10	condensed	8.07	2.78e+03	7.53	2.83e+03
Zhang2009-F5-01	X=0.4	F	diagonal-crossing-time	14400:10	condensed	4.69	7.65e+03	7.09	3.49e+03
Zhang2009-F5-02	X=0.4	F	diagonal-crossing-time	14400:10	condensed	7.73	3.27e+03	6.19	4.74e+03
Zhang2009-F5-03	X=0.4	F	diagonal-crossing-time	14400:10	condensed	1	1.3e+04	3.2	9.1e+03
Zhang2009-F5-04	X=0.4	F	diagonal-crossing-time	14400:10	condensed	3.29	9.67e+03	2.08	1.07e+04
Zhang2009-F5-05	X=0.4	F	diagonal-crossing-time	14400:10	condensed	1	1.3e+04	1.71	1.13e+04
Zhang2009-F5-06	X=0.4	F	diagonal-crossing-time	14400:10	condensed	0.332	1.39e+04	0.3	1.33e+04
Zhang2009-F5-07	X=0.4	F	diagonal-crossing-time	14400:10	condensed	0.0198	1.44e+04	0.08	1.37e+04
Zhang2009-F5-00	X=0.4	F	completion-time	5.01	condensed	5.01	1.13e+03	5.01	1.07e+03
Zhang2009-F5-01	X=0.4	F	completion-time	5.01	condensed	5.01	8.54e+03	5.01	1.47e+03
Zhang2009-F5-02	X=0.4	F	completion-time	5.01	condensed	5.01	1.54e+03	5.01	3.1e+03
Zhang2010-F3A-00	C=10	ROX	diagonal-crossing-time	21600:100	condensed	95.2	1.05e+03	88.1	2.56e+03
Zhang2010-F3A-00	C=1	ROX	diagonal-crossing-time	21600:100	condensed	71.6	6.14e+03	44.6	1.18e+04
Zhang2010-F3A-00	C=10	ROX	completion-time	50	condensed	50	404	50	612
Zhang2010-F3A-00	C=1	ROX	completion-time	50	condensed	50	3.91e+03	50	1.6e+04
Zhang2010-F3B-00	C=3	ROX	diagonal-crossing-time	21600:30	condensed	26.1	2.85e+03	24.1	4.2e+03
Zhang2010-F3B-00	C=0.9	ROX	diagonal-crossing-time	21600:30	condensed	20.8	6.6e+03	17.4	9.05e+03
Zhang2010-F3B-00	C=3	ROX	completion-time	15	condensed	15	1.35e+03	15	1.57e+03
Zhang2010-F3B-00	C=0.9	ROX	completion-time	15	condensed	15	4.37e+03	15	6.94e+03
Zhang2010-F3C-00	C=0.9	ROX	diagonal-crossing-time	43200:3	condensed	2.37	9.04e+03	2.3	1.02e+04
Zhang2010-F3C-00	C=0.3	ROX	diagonal-crossing-time	43200:3	condensed	1.82	1.7e+04	1.82	1.7e+04
Zhang2010-F3C-00	C=0.9	ROX	completion-time	1.5	condensed	1.5	4.85e+03	1.5	4.49e+03
Zhang2010-F3C-00	C=0.3	ROX	completion-time	1.5	condensed	1.5	1.34e+04	1.5	1.24e+04
Zhang2010-F3D-00	C=1	ROX	diagonal-crossing-time	86400:1	condensed	0.817	1.58e+04	0.83	1.44e+04
Zhang2010-F3D-00	C=0.1	ROX	diagonal-crossing-time	86400:1	condensed	0.516	4.18e+04	0.53	4.04e+04
Zhang2010-F3D-00	C=1	ROX	completion-time	0.5	condensed	0.5	6.46e+03	0.5	5.35e+03
Zhang2010-F3D-00	C=0.1	ROX	completion-time	0.5	condensed	0.5	4.03e+04	0.5	3.63e+04
Zhang2011-F3A-00	T1=18 T2=18	F	diagonal-crossing-time	1800:12	condensed, k_slow=0.001, k_fast=0.1	11.3	117	9.15	409
Zhang2011-F3A-00	T1=12 T2=12	F	diagonal-crossing-time	1800:8	condensed, k_slow=0.001, k_fast=0.1	7.26	169	6.14	426
Zhang2011-F3A-00	T1=6 T2=6	F	diagonal-crossing-time	1800:4	condensed, k_slow=0.001, k_fast=0.1	3.12	394	3.25	300
Zhang2011-F3A-00	T1=18 T2=18	F	completion-time	50%	condensed, k_slow=0.001, k_fast=0.1	9.02	82	9.02	381
Zhang2011-F3A-00	T1=12 T2=12	F	completion-time	50%	condensed, k_slow=0.001, k_fast=0.1	6.01	111	6.01	385
Zhang2011-F3A-00	T1=6 T2=6	F	completion-time	50%	condensed, k_slow=0.001, k_fast=0.1	3.02	362	3.02	264
Kotani2017-F2-00	C1=1	D	diagonal-crossing-time	32400:10	condensed, release_cutoff=7	8.36	5.32e+03	7.49	4.89e+03
Kotani2017-F2-00	C1=0.5	D	diagonal-crossing-time	32400:10	condensed, release_cutoff=7	7.55	7.95e+03	6.29	9.39e+03
Kotani2017-F2-00	C1=0.05	D	diagonal-crossing-time	32400:10	condensed, release_cutoff=7	3.26	2.18e+04	1.35	2.74e+04
Kotani2017-F2-00	C1=1	D	completion-time	5	condensed, release_cutoff=7	5	2.01e+03	5	555
Kotani2017-F2-00	C1=0.5	D	completion-time	5	condensed, release_cutoff=7	5	3.92e+03	5	4.51e+03
Kotani2017-F3-00	C1=0.1	D	diagonal-crossing-time	97200:10	condensed, release_cutoff=7	6.78	3.14e+04	7.78	2.14e+04
Kotani2017-F3-00	C1=0.01	D	diagonal-crossing-time	97200:10	condensed, release_cutoff=7	2.36	7.43e+04	3.17	6.61e+04
Kotani2017-F3-00	C1=0.001	D	diagonal-crossing-time	97200:10	condensed, release_cutoff=7	0.335	9.4e+04	0.74	9.07e+04
Kotani2017-F3-00	C1=0.1	D	completion-time	5	condensed, release_cutoff=7	5	1.92e+04	5	1.3e+04
Kotani2017-F4-00	C1=0.1	D	diagonal-crossing-time	18000:10	condensed, k_slow=0.0001, k_fast=0.001	6.55	6.22e+03	6.11	6.8e+03
Kotani2017-F4-00	C1=0.01	D	diagonal-crossing-time	18000:10	condensed, k_slow=0.0001, k_fast=0.001	6.07	7.08e+03	4.88	9.05e+03
Kotani2017-F4-00	C1=0.001	D	diagonal-crossing-time	18000:10	condensed, k_slow=0.0001, k_fast=0.001	5.56	7.99e+03	4.21	1.03e+04
Kotani2017-F4-00	C1=0	D	diagonal-crossing-time	18000:10	condensed, k_slow=0.0001, k_fast=0.001	0	1.8e+04	3.85	1.09e+04
Kotani2017-F4-00	C1=0.1	D	completion-time	5	condensed, k_slow=0.0001, k_fast=0.001	5	4.68e+03	5	6.13e+03
Kotani2017-F4-00	C1=0.01	D	completion-time	5	condensed, k_slow=0.0001, k_fast=0.001	5	6.08e+03	5	9.13e+03
Kotani2017-F4-00	C1=0.001	D	completion-time	5	condensed, k_slow=0.0001, k_fast=0.001	5	7.5e+03	5	1.08e+04
Kotani2017-F4-00	C1=0	D	completion-time	5	condensed, k_slow=0.0001, k_fast=0.001	5	nan	5	1.15e+04

Table 8: Data for Fig. 11 Part 2/2.

3 Seesaw semantics background

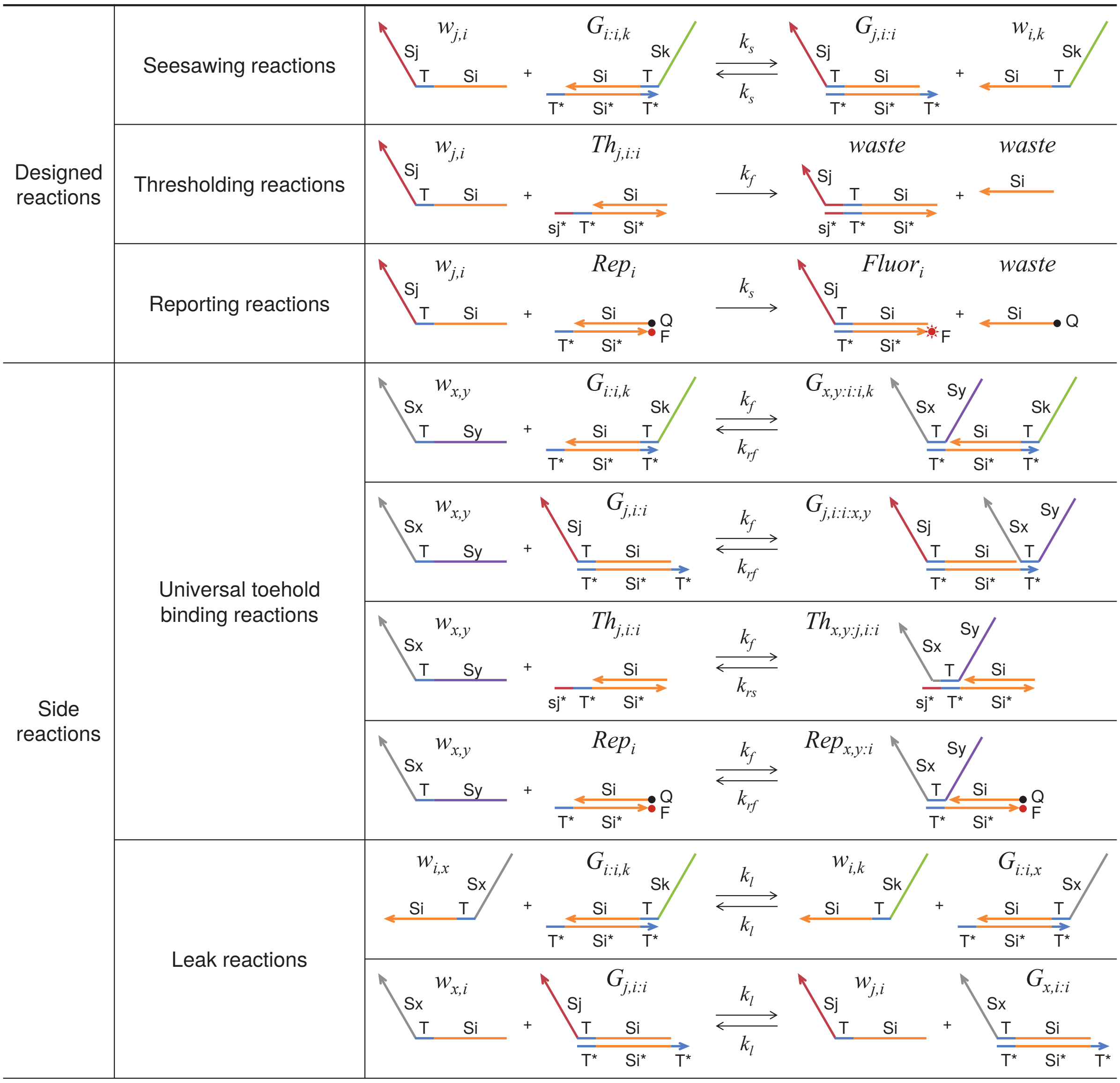

Fig. 1: Seesaw compiler reaction semantics. Note the reaction rate k_{rs} is indistinguishable from k_{rf} in Peppercorn's semantics. Figure taken from Qian & Winfree (2011) [4] supporting online material.

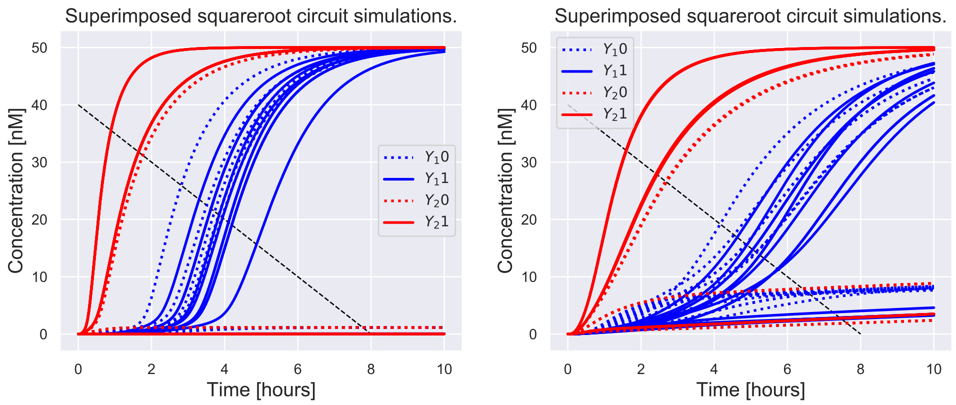

Temperature: 25 °C		
k_s	=	$5 \times 10^4\ \mathrm{M^{-1}s^{-1}}$
k_f	=	$2 \times 10^6\ \mathrm{M^{-1}s^{-1}}$
k_{rs}	=	$1.3\ \mathrm{s^{-1}}$
k_{rf}	=	$26\ \mathrm{s^{-1}}$
k_l	=	$10\ \mathrm{M^{-1}s^{-1}}$

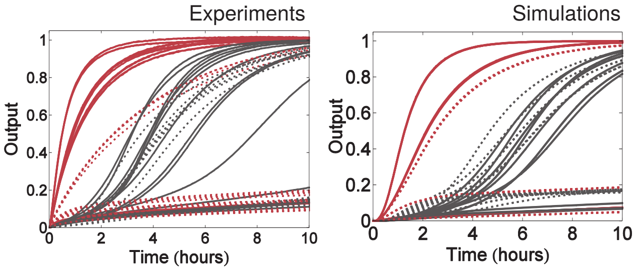

Fig. 2: Comparison of superimposed squareroot circuit simulations. **(top left)** Peppercorn model with adjusted toehold binding strength. **(top right)** Seesaw compiler model. **(bottom)** Original plots taken from Qian & Winfree (2011)[4] supporting online material.

Bibliography

[1] Robert M Dirks and Niles A Pierce. Triggered amplification by hybridization chain reaction. *Proceedings of the National Academy of Sciences*, 101:15275–15278, 2004.

[2] Suvir Venkataraman, Robert M Dirks, Paul Wilhelm Karl Rothemund, Erik Winfree, and Niles A Pierce. An autonomous polymerization motor powered by DNA hybridization. *Nature Nanotechnology*, 2:490–494, 2007.

[3] Robert Tarjan. Depth-first search and linear graph algorithms. *SIAM Journal on Computing*, 1:146–160, 1972.

[4] Lulu Qian and Erik Winfree. Scaling up digital circuit computation with DNA strand displacement cascades. *Science*, 332:1196–1201, 2011.
